# Measuring eHealth Literacy in the European Economic Area, Switzerland, and the United Kingdom: Scoping Review

**DOI:** 10.2196/87461

**Published:** 2026-05-22

**Authors:** Jonah Valentin Weist, Julia Nitsche, Jan Peter Ehlers, Theresa Sophie Busse

**Affiliations:** 1 Junior Professorship for Digital Health, Faculty of Health, Witten/Herdecke University, Alfred-Herrhausen-Straße 50, Witten, 58455, Germany, +49 2302 926-708; 2 Chair of Didactics and Educational Research in Healthcare, Faculty of Health, Witten/Herdecke University, Witten, Germany

**Keywords:** assessment, digital health, eHealth, electronic health literacy, health literacy, measurement instruments, operationalization, scoping review, telehealth, telemedicine

## Abstract

**Background:**

Digital tools continue to evolve and have the potential to improve health care delivery. However, they are associated with challenges, including accessibility issues and health misinformation. Individuals need eHealth literacy (eHL) to reliably use these tools, and providers require appropriate eHL measurement approaches to offer targeted solutions. For around 2 decades, researchers have been operationalizing and measuring eHL.

**Objective:**

This paper aims to provide an up-to-date overview of how eHL has been assessed in recent years in the European Economic Area, Switzerland, and the United Kingdom and which methodological limitations need to be considered.

**Methods:**

A scoping review was conducted. Records were searched via CINAHL, PubMed, and Google Scholar on January 31, 2025, and January 28, 2026. Peer-reviewed empirical papers published in German or English since 2020 that measured eHL in the European Economic Area, Switzerland, or the United Kingdom were included. The synthesis covered the publication trend, eHL measurement approaches and associated limitations reported in the included papers, eHL measurement frequency, countries and languages, and samples.

**Results:**

In the final analysis, 132 papers published between 2020 and 2025 were included. The number of publications per year showed an overall upward trend (2020: 11/132, 8.33%; 2025: 35/132, 26.52%). Nine self-report eHL measurement instruments were used, the eHealth Literacy Scale being the most frequent (94/132, 71.21%). All included papers (132/132, 100%) reported data collection via surveys, and digital surveys were common (71/132, 53.79%). Reported limitations included potential self-report biases (37/132, 28.03%), selection biases due to the data collection modes (26/132, 19.70%), and limitations specific to 5 eHL measurement instruments (18/132, 13.64%). Most included papers (121/132, 91.67%) reported eHL results from a single measurement per participant. Data were collected in 22 countries within the target regions. The distribution of eHL measurement instruments varied considerably between countries. Data were collected in 22 languages within the target regions. Patients or individuals with health problems were a frequent target group (64/132, 48.48%). Most papers (104/132, 78.79%) described studies covering broad adult age ranges. Sample sizes ranged from ≤50 (7/132, 5.30%) to >2000 participants (8/132, 6.06%).

**Conclusions:**

To our knowledge, this is the first scoping review synthesizing eHL measurement limitations reported in empirical papers from the European Economic Area, Switzerland, and the United Kingdom. The identified limitations reported in the included papers potentially lead to biased results. Therefore, health care providers and researchers should take various factors into account when selecting eHL measurement approaches, such as eHL measurement purpose, target population, and data collection setting. Future research should address these constraints by adapting and developing new or revised eHL measurement instruments, including translated and culturally adapted versions. Policymakers should encourage health care providers to conduct methodologically well-founded eHL measurements as a basis for targeted solutions.

## Introduction

### Rationale

The internet is used by many individuals in the context of health. In a 2024 survey conducted in the European Union, 58% of the participants aged between 16 and 74 years reported that they had used the internet to search for health-related information within the last 3 months [1]. The internet offers more than access to health-related information. Today, a variety of digital tools offer further use cases in the context of health. For example, users can generate health information via generative artificial intelligence (GenAI)–based chatbots [2] and share health information or interact with others via social media platforms [3]. In addition, dedicated health applications such as Apple Health [4] and Google Fit [5] help individuals track health goals via smartphones and smart watches. The World Health Organization provides an overview of digital interventions for individuals in the context of health that enable communication, tracking, reporting, financial transactions, and consent management [6].

However, digital tools are also associated with obstacles for individuals who lack access to hardware and internet or digital literacy [7]. Subgroups, such as older adults, are particularly at risk of digital exclusion [8,9]. Furthermore, users are confronted with misinformation on health topics. A review of studies that analyzed health-related posts on social media platforms such as Facebook, Instagram, and Twitter, now called X, indicates that health misinformation is widespread [10]. Similarly, studies show that GenAI models generate unreliable health information in some cases [11,12]. Users who follow incorrect advice generated by ChatGPT may be exposed to serious health risks [12].

As often argued in the literature, sufficient eHealth literacy (eHL) is required for the reliable use of digital tools in the context of health [13-16]. Norman and Skinner [13] proposed an early and influential definition of eHL in 2006, long before the GenAI era. They defined eHL as “the ability to seek, find, understand, and appraise health information from electronic sources and apply the knowledge gained to addressing or solving a health problem” ([13], p. 1). Information on eHL levels is relevant for providers of digital interventions to develop targeted solutions or physicians to suggest appropriate digital health interventions and to evaluate the level of support required by individual patients [17].

Different instruments have been developed to measure eHL and used to varying degrees in empirical research. The most common eHL measurement instrument is the eHealth Literacy Scale (eHEALS) [18-20]. The eHEALS is an 8-item self-report measure developed by Norman and Skinner [17] and published in 2006. It was initially tested among students aged 13 to 21 years [17] and has been used in a variety of studies, involving internet users and the general population. However, researchers pointed out limitations of the eHEALS, including its one-dimensionality [21,22], insufficient capture of actual eHL levels due to the risk of overestimation and underestimation [14,23], and lack of items related to shifting use cases, such as social media [14,24]. Researchers also argued that the underlying Lily model, named after its visual arrangement of 6 literacies around eHL—traditional literacy, information literacy, media literacy, computer literacy, scientific literacy, and health literacy [13,17]—does not cover important aspects of eHL, such as the ability to formulate health questions; know-how in using digital technologies; and contextual, cultural, and communicative competencies [15] or individual, situational, and environmental factors [25]. Another aspect missing in the Lily model is mentioned in the eHealth Literacy Framework (eHLF) by Norgaard et al ([26], p. 533): “safety and control,” particularly related to personal health data. In light of the conceptual discourse and shifting use cases, further eHL measurement instruments have been developed. Prominent examples are the multidimensional eHealth Literacy Questionnaire (eHLQ) [27], which is based on the eHLF [26], and the Digital Health Literacy Instrument (DHLI), which covers skills related to social media [14]. Similar to the eHEALS [17], these instruments provide eHL scores based on self-reports [14,27]. However, the DHLI is at least supplemented by a set of performance-based items [14]. A review indicates that performance-based approaches are less common in empirical research [18]. In March 2025, Norman, Skinner, and colleagues [28] published an updated definition of eHL, which takes “effectiveness, safety and helpfulness” into account and is as follows: “the ability to engage with digital technologies in effective, safe, and helpful ways to achieve health goals” ([28], p. 1). They also updated the Lily model, which now considers contextual factors surrounding eHL, and developed a revised 10-item version of the eHEALS based on the new Lily model, which has not yet been published [28].

Overall, the outlined historical development shows that the definition and operationalization of eHL has changed since the initial definition by Norman and Skinner [13] was published in 2006. Measuring eHL in a fast-paced digital environment is a challenging task. Researchers and health care providers not only need suitable eHL measurement instruments but also need to select appropriate data collection methods and modes, especially if they aim to assess the eHL of vulnerable subgroups. For example, the use of online surveys can lead to an underrepresentation of non-internet users [29]. To provide guidance for researchers, practitioners, and policymakers, there is a need for an up-to-date overview of current eHL measurement instruments, data collection methods and modes, and associated limitations. The latter is particularly important, as existing reviews in the field of eHL measurement [eg, 18-20] did not systematically analyze eHL measurement–related limitations reported in the included papers.

### Objectives

This paper has two central objectives: (1) to provide an overview of eHL measurement instruments as well as data collection methods and modes recently used in empirical research in the European Economic Area, Switzerland, and the United Kingdom (ie, in countries that are well comparable in terms of health care system standards, culture, socioeconomic aspects, and data protection) and (2) to examine the limitations of the eHL measurement instruments and data collection approaches reported in the included papers. In addition, it aims to analyze the overall publication trend, eHL assessment frequency per participant, countries of data collection, geographical distribution of eHL measurement instruments, data collection languages, target groups, and sample sizes.

The following research question served as the basis for this paper: how has eHL been assessed in the European Economic Area, Switzerland, and the United Kingdom in recent years, and which methodological limitations need to be considered?

## Methods

Given the objective of this paper, to provide a broad overview of empirical papers in the field of eHL measurement and associated limitations, a scoping review was conducted. The scoping review is reported according to the PRISMA-ScR (Preferred Reporting Items for Systematic reviews and Meta-Analyses Extension for Scoping Reviews) (Checklist 1)[30].

### Protocol and Registration

The study protocol was published on PROSPERO on February 07, 2025 (CRD42025642890). It was updated on November 06, 2025, as the scope and methods—including the eligibility criteria to reduce the number of relevant records—were refined, and again on March 05, 2026, following the updated searches conducted on January 28, 2026. As can be seen in the protocol, the study was initially planned as a rapid review but evolved into a scoping review, which maps existing eHL measurement approaches and associated limitations.

### Eligibility Criteria

Multimedia Appendix 1 provides an overview of the inclusion and exclusion criteria developed by TSB and JVW in consultation with JPE and JN. Papers that described empirical studies and were published in peer-reviewed academic journals were included. Papers had to be published in 2020 or later for two reasons: (1) rapid technological advances and associated new use cases in the context of health, for example, in the field of GenAI [2] and (2) regulatory changes. For example, Germany introduced the Digital Care Act (Digitale-Versorgung-Gesetz [DVG]) in 2019, which has regulated and enabled the reimbursement of prescribable digital health applications (Digitale Gesundheitsanwendungen [DiGA]) [31]. Regulatory measures regarding the regulation and reimbursement of digital health interventions have been taken in other European countries as well [32,33]. In addition, papers were included only if abstracts and full texts were available in either English or German. Papers had to describe studies with a clear geographic focus on the European Economic Area, Switzerland, and the United Kingdom. These countries are well comparable in terms of culture and socioeconomic aspects and have developed health care systems. Furthermore, data protection is strictly regulated: in the European Economic Area by the General Data Protection Regulation (GDPR) [34], in Switzerland by the Federal Act on Data Protection (FADP) [35], and in the United Kingdom by the UK GDPR and the Data Protection Act 2018 [36]. Papers had to report the use of all items or subscales of an original eHL measurement instrument intended for calculating eHL scores.

Papers were excluded if they were reviews. Study protocols, conference papers, gray literature, and book chapters were also excluded. Moreover, cross-country studies without a clear focus on the target regions were excluded. In addition, papers were excluded if they reported the use of a measurement instrument not capturing the skills described in Norman and Skinner’s [13] 2006 definition of eHL, the use of a measurement instrument solely for eHL subdimensions(eg, traditional literacy, health literacy, information literacy, scientific literacy, media literacy, and computer literacy [13]), or the use of a context-specific eHL measurement instrument (eg, COVID-19 or health conditions). Furthermore, studies that had a clear focus on adolescents or children but not on adults were excluded. Studies involving individuals with a professional perspective (eg, health professionals, IT specialists, teachers, and trainers, as well as students in health, IT, education, or communication study programs) were also excluded given the focus of this paper on eHL measurement instruments that are suitable for the general population, patients, parents, or informal caregivers.

It should be noted that it was not initially planned to exclude papers without a specific geographic focus on the European Economic Area, Switzerland, and the United Kingdom. However, after the title-abstract screening of the records from the initial searches, many records from a variety of countries with differences in culture, socioeconomic conditions, data protection regulation, and health care quality remained. To improve the comparability and facilitate the synthesis, the geographic focus eligibility criterion was added. The search strategy itself would not have changed if this criterion had been defined from the beginning.

### Search Process

The reporting of the search process adheres to the PRISMA-S (Preferred Reporting Items for Systematic Reviews and Meta-Analyses Literature Search Extension) checklist [37].

#### Information Sources

To identify a wide range of potentially relevant records from different subject areas, the databases PubMed and CINAHL were separately searched (ie, not simultaneously on a single platform). Supplementary searches were conducted via Google Scholar. Study registries, online or print sources, and cited references or citing references were not searched, browsed, or examined. No additional studies were retrieved through contacts or others, and no additional information sources or search methods were used.

#### Search Strategy

All authors collaboratively developed the search strategy. Broad search strings in English and German were used to identify a wide range of possibly relevant records via CINAHL and PubMed. Shorter search strings were used for the supplementary search via Google Scholar, as the search engine does not process long search queries [38]. The search strings were developed on the basis of the population, concept, and context (PCC) framework [39]. As the aim was to identify empirical papers that described studies involving individuals without a professional perspective (population), no subgroup-specific terms were included. Instead, the search strings contained terms and available MeSH terms in CINAHL and PubMed related to measurement instruments (concept) and eHL (context). The initial searches were conducted on January 31, 2025. In these initial searches, records published since 2020 were filtered. The searches were updated on January 28, 2026, to ensure that the review was up to date. The searches were identical, with the exception of the filters for the publication period, which were set to the period starting January 31, 2025 (PubMed), and January 2025 (CINAHL and Google Scholar), to avoid overlap with records that had already been identified in the initial searches. During both the initial and updated searches, filters were applied in CINAHL and PubMed to identify only English and German records with an available abstract. The “[a]pply equivalent subjects” expander was used in CINAHL to identify additional relevant records. Due to the large number of results retrieved in Google Scholar, the “[s]orted by relevance” function was applied, and the first 100 results retrieved via the English search string and the first 100 retrieved via the German search string were included at both search dates (ie, 400 records in total). Multimedia Appendix 2 presents the full search strategies for the CINAHL, PubMed, and Google Scholar (ie, search strings, filters, and expanders). The search strategies were not peer-reviewed.

### Selection of Sources of Evidence

The screening of the records identified via the initial searches and updated searches was carried out separately at 2 different points in time. The method was identical except for one difference. The geographical focus eligibility criterion was applied only during the full-text screening of the records identified via the initial searches and not during the title-abstract screening. During the screening of the records identified via the updated searches, this criterion was applied from the beginning.

In both cases, EndNote was used for automatic deduplication. Further duplicates were later removed manually. ASReview, an open-source machine learning tool, which ranks records based on the estimated relevance, was seen as a solution to handle the high number of records identified [40]. Van de Schoot et al ([40], p. 125) showed that ASReview “can yield far more efficient reviewing than manual reviewing while providing high quality.” The records were divided by language because ASReview may have difficulty processing different languages: (1) English titles and abstracts and (2) German titles or abstracts. As most records were in English, only records with English titles and abstracts were screened in ASReview, while the smaller number of records with German titles or abstracts were screened manually.

To train the ASReview model, an initial training set containing relevant and irrelevant records was required [40]. JVW imported all English records into Rayyan, a digital tool for collaborative screening [41], created a 5% sample using the sampling function, screened the titles and abstracts and identified relevant records: 33 from the sample of the initial searches, and 2 from the sample of the updated searches. To reduce potential bias in the training set, the same number of irrelevant records was randomly selected. TSB and JN reviewed and confirmed both sets of relevant and irrelevant records. The English records were imported into ASReview, and the training set was used to train the ASReview model. ASReview default settings were used (ie, feature extraction technique: term frequency-inverse document frequency (TF-IDF), classifier: naïve Bayes, query strategy: maximum, and balance strategy: dynamic resampling/double). JVW conducted the title-abstract screening in ASReview. As recommended, the records were screened until a predefined stopping criterion was reached [40]. In line with previous review processes [42-44], the ASReview stopping criterion was set at 100 consecutive irrelevant records. The remaining English records were excluded.

Records with German titles or abstracts identified via both searches were manually screened in Rayyan. In both cases, a 25% sample was selected using the sampling function. TSB and JVW independently screened the records in both samples. Conflicts were resolved through discussion between the two authors. JVW screened the remaining records.

English and German full-text reports were retrieved and imported into Rayyan. In both cases, a 25% sample was created using the sampling function; TSB and JVW independently screened these records. Most conflicts were resolved through discussion between both authors. Some conflicts were discussed in the whole research team. JVW screened the remaining full-text reports and consulted TSB in cases of uncertainty to ensure a consistent and accurate selection.

### Data Charting Process

All authors developed a standardized extraction form, tested it in advance, and modified it on the basis of a sample of 5 included papers [39] and reviewer feedback. JVW extracted data from the included papers using MAXQDA (version 24; VERBI Software Consult Sozialforschung GmbH) to manually code relevant text passages according to predefined categories. TSB then verified the correctness of all extracted data.

### Data Items

The final extraction form contained the following deductive categories: (1) author and publication year, (2) title, (3) full-text language, (4) main objective, (5) objective to validate an eHL measurement instrument, (6) data collection period, (7) eHL measurement frequency per participant, (8) (target) age groups, (9) specific participants’ perspectives, (10) country focus, (11) data collection language, (12) sample size in the central eHL measurement part(s), (13) eHL measurement instrument, (14) data collection method and mode, and (15) reported limitations regarding the eHL measurement instrument and data collection method and modes. Additional subcategories were developed inductively based on the material. Information that was not explicitly reported in the included papers was inferred from the context where reasonable, as indicated in Multimedia Appendix 3.

### Synthesis of Results

The synthesis focused on the following eight themes and questions:

1. Publication trend: how did the number of publications develop?
2. eHL measurement instruments: which eHL measurement instruments were used, and which versions were validated in the included papers?
3. Data collection methods and modes: which data collection methods and modes were used?
4. Reported limitations: which limitations regarding eHL measurement instruments and data collection methods and modes were reported?
5. eHL measurement frequency per participant: was eHL measured once or at multiple time points?
6. Countries and languages: in which countries and languages were data collected, how often were the eHL measurement instruments used per country, and in which languages were data collected?
7. Target groups: what were the target groups in terms of health-related perspective and age?
8. Sample sizes: how large were the samples in the central eHL measurement part(s)?

As the primary objective of this paper was to analyze how eHL was measured, the role of eHL as a variable in each included paper was not analyzed. Because the reporting was sometimes insufficiently transparent and traceable, it was not always possible to determine whether the included papers analyzed the same datasets as other included papers. Therefore, to ensure a consistent and objective approach, each included paper was considered in the synthesis, regardless of whether analyses were based on the same datasets.

## Results

### Selection of Sources of Evidence

**Figure 1.**
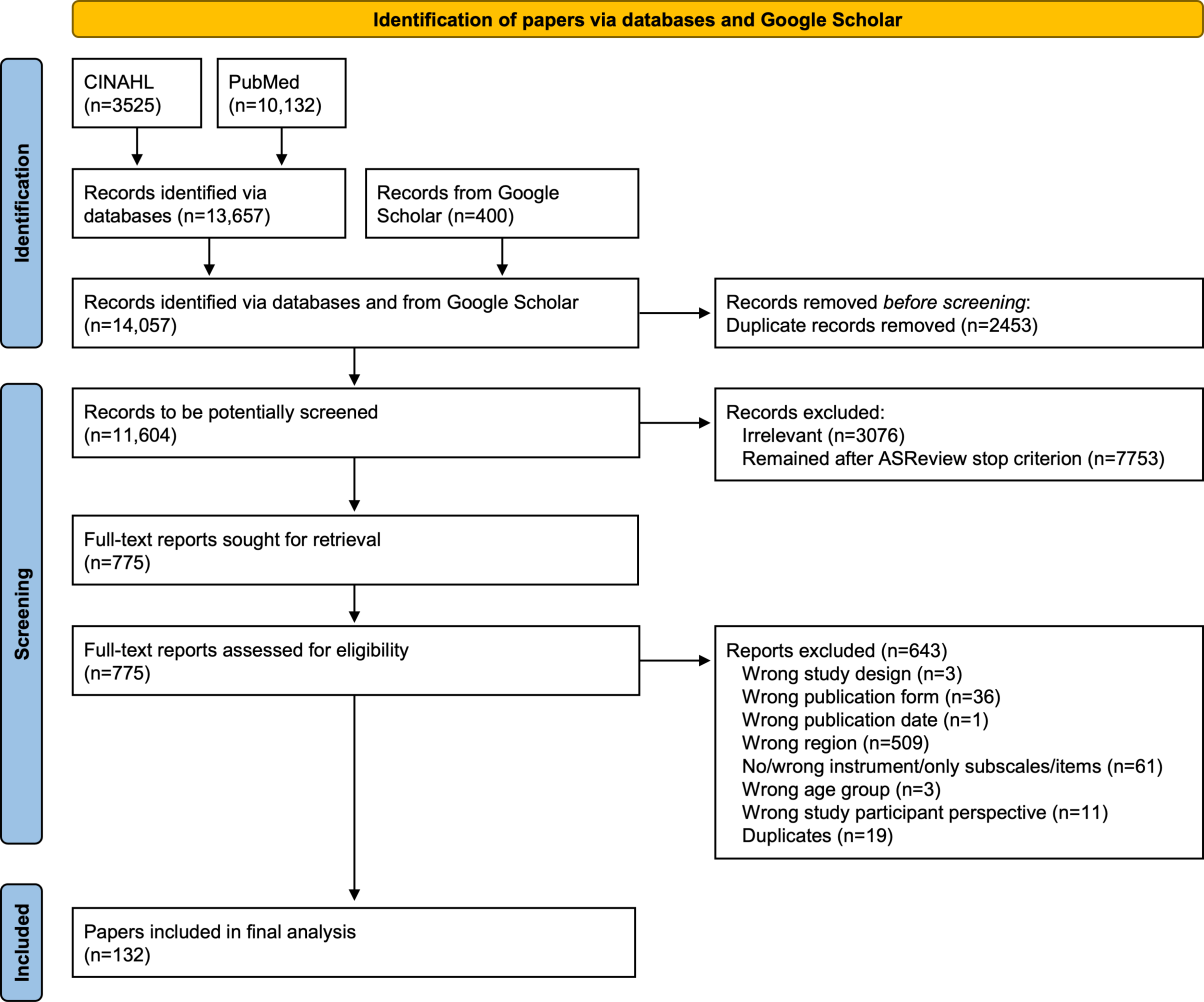
PRISMA (Preferred Reporting Items for Systematic reviews and Meta-Analyses) flow diagram of the paper selection showing the combined results of 2 selection time points (initial and updated searches) (adapted from Page et al [56], which is published under Creative Commons Attribution 4.0 International License [57]).

Through database searches, 13,657 records were identified (CINAHL: n=3525; PubMed: n=10,132). Including the 400 records from Google Scholar, the total number of records was 14,057. Of these, 10,256 records were identified during the initial searches (January 31, 2025) and 3801 records during the updated searches (January 28, 2026). Of the records from the initial searches, 1944 duplicates were automatically removed using EndNote, resulting in 8312 unique records. Of the records from the updated searches, 509 duplicates were removed, resulting in 3292 unique records. Therefore, the total number of records to be potentially screened was 11,604. While all unique German records from the initial searches (113/113, 100%) and the updated searches (100/100, 100%) were manually screened in Rayyan, 2523 of the 8199 unique English records from the initial searches (30.77%) and 1115 of the 3192 unique English records from the updated searches (34.93%) were screened in ASReview until the predefined stopping criterion was reached. In total, 3076 irrelevant records and 7753 that remained after the ASReview stopping criterion was reached were excluded. The full texts of the remaining 775 records were retrieved and assessed for eligibility. For various reasons, 643 full-text reports were excluded. A total of 132 papers were included in the final analysis. Figure 1 shows the combined results of both selection points in time (initial and updated searches).

Some papers might appear to be eligible for inclusion but were excluded. For instance, some papers [eg, 45-48] reported only the use of selected subscales or items. As inclusion in the final analysis of this study required the reported use of all items or subscales of a version of an eHL measurement instrument intended for calculating eHL scores, these papers were excluded. Other examples of excluded papers include those that reported the use of context-specific versions of eHL measurement instruments (eg, related to COVID-19 [eg, 49-54] or chronic pain [55]).

### Characteristics and Results of Sources of Evidence

Multimedia Appendix 3 presents the metadata extracted from the included papers [22,58-188].

### Synthesis of Results

#### Publication Trend

**Figure 2.**
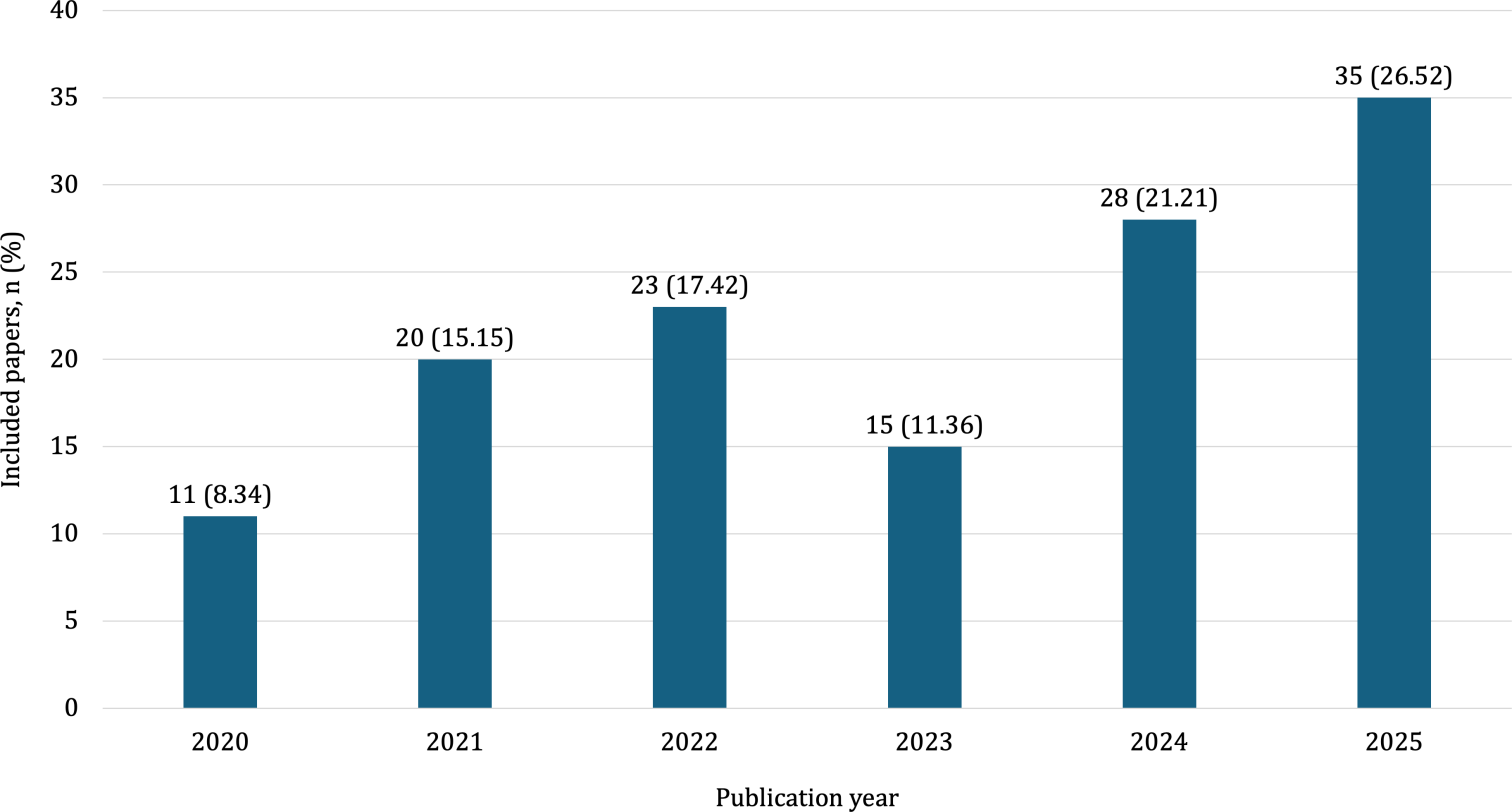
Distribution of the included papers by publication year (N=132).

The included papers were published between 2020 and 2025, with the average number of publications per year being 22 (SD 8.72). As shown in Figure 2, the number of publications per year showed an overall upward trend. Data collection took place between 2016 and 2025.

#### eHealth Literacy Measurement Approaches and Associated Limitations

##### Measurement Instruments

The included papers reported the use of all items or subscales intended for calculating eHL scores of versions of a total of 9 measurement instruments in various languages:

- the eHealth Literacy Scale (eHEALS),
- the eHealth Literacy Questionnaire (eHLQ),
- the digital health literacy module of the Health Literacy Survey 2019‐2021 (HLS_19_-DIGI),
- the Readiness and Enablement Index for Health Technology (READHY),
- the Digital Health Literacy Instrument (DHLI),
- the eHealth Literacy and Use Scale (eHLUS),
- the revised eHealth Literacy Scale-Extended (eHEALS-E),
- the eHealth Literacy Scale for Carers of People with Chronic Diseases (eHEALS-Carer), and
- the Transactional eHealth Literacy Instrument (TeHLI)

The eHEALS was by far the most common eHL measurement instrument (94/132, 71.21%). The eHLQ was a distant second (20/132, 15.15%), followed by the HLS_19_-DIGI (9/132, 6.82%) and the READHY (7/132, 5.30%). Of the included papers, 2 each reported the use of the DHLI (2/132, 1.52%), the eHLUS (2/132, 1.52%), and the revised eHEALS-E (2/132, 1.52%). One paper each reported the use of the eHEALS-Carer (1/132, 0.76%) and the TeHLI (1/132, 0.76%). Six of the 132 included papers(4.54%) reported the use of all items or subscales intended for calculating eHL scores of 2 of the identified eHL measurement instruments: the eHEALS and the eHLUS (2/132, 1.52%) [91,166], the eHEALS and the eHLQ (2/132, 1.52%) [97,98], the eHEALS and the revised eHEALS-E (1/132, 0.76%) [86], and the eHEALS and the TeHLI (1/132, 0.76%) [160]. Table 1 lists the original eHL measurement instruments, including the initial developer and the year of development and the underlying concept or instrument.

**Table 1.** eHealth literacy measurement instruments identified in the included papers (N=132).^a^

Instrument	Initial developer and year	Underlying concept or instrument	Papers, n (%)	Citations
eHEALS^b^	Norman and Skinner, 2006 [17]	Lily model [13]	94 (71.21)	[58-60,62,65-68,70-84,86,87,91-95,97-102,104-107,109-111,113-116,119-125,127-135,137-141,143,145,147,149,153,155,157,160,162,164-168,172,174,176,178,181-188]
eHLQ^c^	Kayser et al, 2018 [27]	eHLF^d^ [26]	20 (15.15)	[61,63,88-90,96-98,103,112,118,126,142,146,156,158,159,163,173,179]
HLS_19_-DIGI^e^	HLS_19_ Consortium of the WHO Action Network M-POHL, 2022 [189]	DHLI^f^ [14], eHEALS [17]	9 (6.82)	[69,108,117,136,150-152,171,177]
READHY^g^	Kayser et al, 2019 [190]	eHLQ [27], heiQ^h^ [191], HLQ^i^ [192]	7 (5.30)	[64,144,148,161,169,170,175]
DHLI	Van der Vaart et al, 2017 [14]	Skills identified in a qualitative bottom-up study [193]	2 (1.52)	[154,180]
eHLUS^j^	Stephan et al, 2025 [166]	Theoretical framework developed based on literature search, German eHEALS [194]	2 (1.52)	[91,166]
Revised eHEALS-E^k^	Petrič and Atanasova, 2024 [22]	Initial eHEALS-E [21], eHEALS [17], Norman and Skinner’s [13] definition of eHL	2 (1.52)	[22,86]
eHEALS-Carer^l^	Efthymiou et al, 2019 [195]	eHEALS [17]	1 (0.76)	[85]
TeHLI^m^	Paige et al, 2019 [196]	TMeHL^n^ [197]	1 (0.76)	[160]

a Six included papers (6/132, 4.54%) appear twice in the table, as they reported the use of all items or subscales intended for calculating scores of versions of 2 eHL measurement instruments [86,91,97,98,160,166].

b eHEALS: eHealth Literacy Scale.

c eHLQ: eHealth Literacy Questionnaire.

d eHLF: eHealth Literacy Framework.

e HLS_19_-DIGI: digital health literacy module of the Health Literacy Survey 2019‐2021.

f DHLI: Digital Health Literacy Instrument.

g READHY: Readiness and Enablement Index for Health Technology.

h heiQ: Health Education Impact Questionnaire.

i HLQ: Health Literacy Questionnaire.

j eHLUS: eHealth Literacy and Use Scale.

k eHEALS-E: eHealth Literacy Scale-Extended.

l eHEALS-Carer: eHealth Literacy Scale for Carers of People with Chronic Diseases.

m TeHLI: Transactional eHealth Literacy Instrument.

n TMeHL: Transactional Model of eHealth Literacy.

Multimedia Appendix 4 summarizes the characteristics of the identified original eHL measurement instruments. Notably, all identified measurement instruments provide eHL scores based on self-reports. The DHLI is the only identified eHL measurement instrument, which contains supplementary performance-based items [14]. Both included papers reporting the use of the DHLI (2/132, 1.52%) did not report the use of these items [154,180].

Of the 132 included papers, 23 (17.42%) reported the objective to validate an eHL measurement instrument. These papers were grouped into two clusters:

1. Two of the 132 included papers (1.52%) reported the objective to validate a new eHL measurement instrument: the eHLUS (1/132, 0.76%) [166] and the revised eHEALS-E (1/132, 0.76%) [22]. Although an earlier version of the eHEALS-E was published in 2017 [21], the revised version validated in the included paper [22] is considered a new eHL measurement instrument in this scoping review because it contains different items.
2. Twenty-one of the 132 included papers (15.91%) reported the objective to validate a version of at least 1 already existing eHL measurement. These were versions of the eHEALS (15/132, 11.36%) [62,71-73,75,86,92,93,104,122-124,131,181,182], the eHLQ (4/132, 3.03%) [96,97,142,158], the revised eHEALS-E (1/132, 0.76%) [86], and the TeHLI (1/132, 0.76%) [160]. One included paper (1/132, 0.76%) reported the objective to validate such a version of 2 of the identified instruments: the eHEALS and the revised eHEALS-E [86]. In addition, 1 included paper (1/132, 0.76%) aimed to validate the HLS_19_-DIGI in various languages across 13 countries [117].

Multimedia Appendix 5 provides an overview of characteristics of the validation papers and the eHL measurement instruments.

Some translated or adapted versions used or validated in the included papers differ from the original instruments in key aspects, such as dimensionality (eg, versions of the eHEALS showed a 2-factor [62,73,75,86,92,123,194] or 3-factor structure [71] unlike the original eHEALS [17]) and number of items (eg, the Greek version of the revised eHEALS-E [86] comprises 30 items from the initial item pool of the revised eHEALS-E [22], and the Greek version of the eHEALS [86] showed a better fit with 6 items). Further examples of deviations include the reported use of the eHEALS with a different response scale—for example, the reported use of a “7-point Likert scale” ([139], p. 9) instead of the original 5-point Likert scale [17]—and 2 supplementary eHEALS items for calculating eHL scores [105,134].

### Data Collection Methods and Modes

All included papers (132/132, 100%) reported that data on eHL were collected via surveys. As shown in Table 2, most included papers (71/132, 53.79%) reported or implied that surveys were administered digitally (eg, online or on a tablet). Twenty-three of the 132 included papers (17.42%) reported or implied that 2 or 3 data collection modes were used. One of these (1/132, 0.76%) described a cross-country study in which different modes (1 or 2) were used depending on the country [117].

**Table 2.** Data collection modes identified in the included papers (N=132).^a^

Mode	Papers, n (%)	Citations
Digital	71 (53.79)	[22,59,60,62,63,70,72,73,79-84,86,91,92,97,98,101-105,107-109,112,115-121,123-127,129-133,136,138-143,153-156,160-162,166,167,172,173,175,178,183-188]
Paper-based	44 (33.33)	[22,61,63-67,71,75,85,87-90,93,95,96,99,100,104,106,111,113,114,126,127,132-135,146,149,158,159,161,162,164,167,170-172,178,183,184]
Face-to-face	16 (12.12)	[22,64,88-90,98,117,122,128,144,147,150-152,170,177]
Telephone-based	9 (6.82)	[69,76-78,117,118,137,144,162]
Not reported	18 (13.64)	[58,68,74,94,110,145,148,157,163,165,168,169,174,176,179-182]

a Of the 132 included papers, 23 (17.42%) are listed more than once as they reported or implied that 2 or 3 data collection modes were used [22,63,64,88-90,98,104,117,118,126,127,132,133,144,161,162,167,170,172,178,183,184].

### Reported Limitations

#### Clusters of Reported Limitations

By reviewing the *Limitations* sections or, if these were not available, the *Discussion* sections, it was identified that 65 of the 132 included papers (49.24%) reported at least 1 limitation related to the applied eHL measurement instruments and eHL-related data collection methods and modes [22,58,59,62,66,67,72,73,75-79,81,82,86,87,92,97,98,104,106-109,111,112,114-117,120,122-125,128-131,134,135,137,143,144,146,152,156-159,163,166,167,170-174,180,181,183,185-187]. Four clusters were identified: (1) self-report biases, (2) selection biases due to the data collection modes, (3) instrument-specific limitations, and (4) other limitations associated with the data collection methods and modes.

#### Self-Report Biases

Notably, 37 of the 132 included papers (28.03%) reported the risk of self-report biases associated with the use of self-report instruments [22,58,59,62,72,73,75-77,92,97,104,106,108,109,111,112,114,123-125,130,131,134,135,137,146,152,157,159,167,171-174,181,185]. Of these, some explicitly mentioned particular forms or causes, including overestimation or underestimation of actual skills (8/132, 6.06%) [73,77,111,157,159,171,172,181], inaccurate or socially desirable responses (6/132, 4.54%) [22,77,111,131,134,171], recall difficulties (3/132, 2.27%) [77,135,137], comprehension issues (1/132, 0.76%) [75], and perspective shifts (1/132, 0.76%) [104].

#### Selection Biases Due to the Data Collection Modes

Twenty-six of the 132 included papers (19.70%) reported potential selection biases due to the data collection modes [62,66,67,79,81,82,92,97,98,109,115-117,120,123,124,129,130,143,146,156,167,173,183,186,187]. Most of these (22/132, 16.67%) noted that data collection via online surveys potentially led to an overrepresentation or underrepresentation of subgroups with specific characteristics and provided specific examples of potentially underrerepresented subgroups [62,79,81,82,92,97,98,109,115-117,120,123,124,129,130,143,156,167,173,186,187], including low digital literacy, less digital experience, or lack of access to hardware or the internet (19/132, 14.39%) [62,79,81,82,92,97,98,109,115,116,120,123,124,129,130,143,167,173,187]; older age (6/132, 4.54%) [79,82,109,130,173,187]; low education or functional literacy (4/132, 3.03%) [130,156,173,186]; low eHL (2/132, 1.52%) [117,167]; low income or wealth (2/132, 1.52%) [186,187]; and residence in rural areas (1/132, 0.76%) [186] (if characteristics were reported only for potentially overrepresented subgroups, these were inverted). Two of the 132 included papers (1.52%) addressed the possibility of selection biases associated with the use of self-administered, paper-based questionnaires, mentioning the exclusion of individuals with a lower level of functional literacy [66,67]. One of the 132 included papers (0.76%) noted the possible overrepresentation or underrepresentation of individuals with low eHL “even though we used paper questionnaires” ([146], p. 682). One of the 132 included papers (0.76%) reported unspecified selection biases because of fewer responses to paper-based questionnaires than to online questionnaires [183].

#### Instrument-Specific Limitations

Eighteen of the 132 included papers (13.64%) reported at least 1 limitation specific to an eHL measurement instrument; however, limitations regarding the validation process of translated and adapted instruments were not analyzed in this scoping review. Eleven of the 132 papers (8.33%) reported eHEALS-specific limitations, including limited comprehensiveness in times of evolving digital use cases (8/132, 6.06%) [73,86,87,120,122,125,143,167] in areas such as “Health 2.0” ([73], p. 8), “Web 2.0” ([122], p. 15), “Web 2.0 and 3.0” ([86], p. 14; [87], p. 9), and “online communities, social media, and AI-based health applications” ([87], p. 9); insufficient coverage of skills related to evaluation of digital health information (1/132, 0.76%) [107]; insufficient explanation of the connection between the eHEALS and the underlying Lily model (1/132, 0.76%) [75]; lack of clarity whether “insufficient health literacy, digital literacy, or a combination hereof” leads to low eHL (1/132, 0.76%) ([75], p. 246); insufficient distinction between individuals with different levels of digital literacy (1/132, 0.76%) [75]; lack of “uniformly used cutoff scores reported in the literature” (1/132, 0.76%) ([128], p. 11); the potential “inability to capture true differences between participants achieving the highest possible score” due to a ceiling effect found in the study (1/132, 0.76%) ([167], p. 9); and the difficulty for participants to perceive “differences between the individual items” ([128], p. 11). Three of the 132 included papers (2.27%) reported eHLQ-specific limitations: the complexity in analyses due to the multidimensional structure (2/132, 1.52%) [163,173], “increasing the risk of serendipitous results” (1/132, 0.76%) ([163], p. 10), and the less extensive psychometric testing compared to the eHEALS (1/132, 0.76%) [146]. Two of the included papers (1.52%) mentioned *READHY*-specific limitations: one of these (1/132, 0.76%), which was published in 2023 and reported the use of an English version of the READHY, addressed the overall complexity and length, potentially leading to selection bias and limiting its practicality [144], whereas the other (1/132, 0.76%), which was published in 2020, mentioned a lack of validated translations into languages other than Danish at the time of the survey [170]. Two of the included papers (2/132, 1.52%) that reported the objective to validate a version of the revised eHEALS-E mentioned the insufficient distinction between 2 dimensions (ie, awareness of sources and recognition of quality and meaning) [22,86]. Further criticism included the following: potential presence of obsolete items and overall length, which could impair the practicality (1/132, 0.76%) [22]; reliance of the items in one dimension (“Being smart on the Net”) on the technological progress, potentially requiring ongoing updates (1/132, 0.76%) [22]; the need to integrate further items concerning “Web 3.0” (1/132, 0.76%) ([86], p. 13); lack of cognitive interviews for some Slovenian items and all English items aimed at improving the wording (1/132, 0.76%) [22]; the availability of validated versions in Slovenian and Greek only at the time of the publication of the included paper (1/132, 0.76%) [86]; and the requirement of further testing of items to avoid social desirability bias (1/132, 0.76%) [22]. One paper (1/132, 0.76%) that aimed to initially validate the eHLUS reported that it is based on eHEALS and pointed out limitations of the development and validation process: potentially limited diversity of experts who participated in the development and validation process; operational challenges during the expert interviews; and the context-specific validation, which may not be generally applicable [166].

#### Other Limitations Associated With the Data Collection Methods and Modes

Other limitations identified to be associated with the data collection methods and modes included smaller sample sizes due to the exclusive use of paper-based surveys (2/132, 1.52%) [158,159], less thoughtful responses due to the use of a telephone-based survey (1/132, 0.76%) [78], and unspecified “obvious inherent limitations” of surveys (1/132, 0.76%) ([180], p. 7). In addition, one included paper (1/132, 0.76%) reported the use of different modes to collect data in different countries, limiting comparability of the results between countries [117].

### Other Findings

#### eHealth Literacy Measurement Frequency Per Participant

Most included papers (121/132, 91.67%) reported eHL results from a single measurement per participant [22,58-67,69,70,72,74-102,104-130,132-159,161,162,164,165,167-175,177-180,183-188]. Seven of the 132 included papers (5.30%) aiming to validate a version of an eHL measurement instrument used a test-retest design [71,73,131,160,166,181,182]. Three of the 132 included papers (2.27%) reported eHL results from baseline eHL measurement and 1 follow-up (1/132, 0.76%) [68] or 2 follow-ups (2/132, 1.52%) [103,176], and 1 included paper (1/132, 0.76%) reported only the results from 2 follow-ups due to a technical error [163].

#### Countries and Languages

##### Countries

The described studies were conducted across 22 countries within the target regions of this study, most frequently in Germany (39/132, 29.54%). Five included papers (5/132, 3.79%) reported multicountry data collection, with 1 paper each reporting data collection in 2 countries (1/132, 0.76%) [85], 4 countries (1/132, 0.76%) [143], 6 countries and additional unspecified countries within the European Union or the European Economic Area (1/132, 0.76%) [59], 7 countries (1/132, 0.76%) [58], and 12 countries within the target regions of this study and 1 country (Israel) located outside the target region of this study (1/132, 0.76%) [117]. Table 3 lists the countries of data collection identified in the included papers.

**Table 3.** Countries of data collection identified in the included papers (N=132).^a^

Country	Papers, n (%)	Citations
Germany	39 (29.54)	[58,59,62,70,74,76,77,87,91,92,99,100,106,108,109,111,114,117,120,123-125,128,130,136,137,143,147,150-154,156,164,166,174,183,184]
Poland	15 (11.36)	[72,78-84,107,134,135,160,165,187,188]
Sweden	15 (11.36)	[65-67,93,94,112,143,157-159,167,168,176,181,182]
Norway	14 (10.61)	[61,64,71,75,96,103,117,118,126,132,133,145,146,172]
Hungary	9 (6.82)	[58,59,69,101,102,117,138,185,186]
United Kingdom	9 (6.82)	[115,116,121,127,129,143,144,162,173]
Denmark	8 (6.06)	[63,117,148,161,163,169,170,179]
Italy	8 (6.06)	[68,119,139,143,149,155,171,180]
Portugal	8 (6.06)	[58-60,90,117,122,131,177]
Spain	6 (4.54)	[58,59,88,89,97,98]
Greece	4 (3.03)	[85,86,105,110]
Cyprus	3 (2.27)	[58,59,85]
Czechia	3 (2.27)	[117,140,141]
Finland	3 (2.27)	[58,95,175]
Slovenia	3 (2.27)	[22,58,59]
Austria	2 (1.52)	[113,117]
France	2 (1.52)	[73,117]
Ireland	2 (1.52)	[117,178]
Switzerland	2 (1.52)	[104,117]
Belgium	1 (0.76)	[117]
Netherlands	1 (0.76)	[142]
Slovakia	1 (0.76)	[117]

a Five included papers (5/132, 3.79%) are listed 2 or 3 times because they reported multicountry data collection [58,59,85,117,143], with one of these describing that participants were also recruited from Israel, which lies outside the target region of this study and is therefore not listed [117]. One of the included papers (1/132, 0.76%) reported 6 countries of data collection and data collection in additional unspecified countries within the European Union or the European Economic Area [59].

##### Geographical Distribution of eHealth Literacy Measurement Instruments

**Figure 3.**
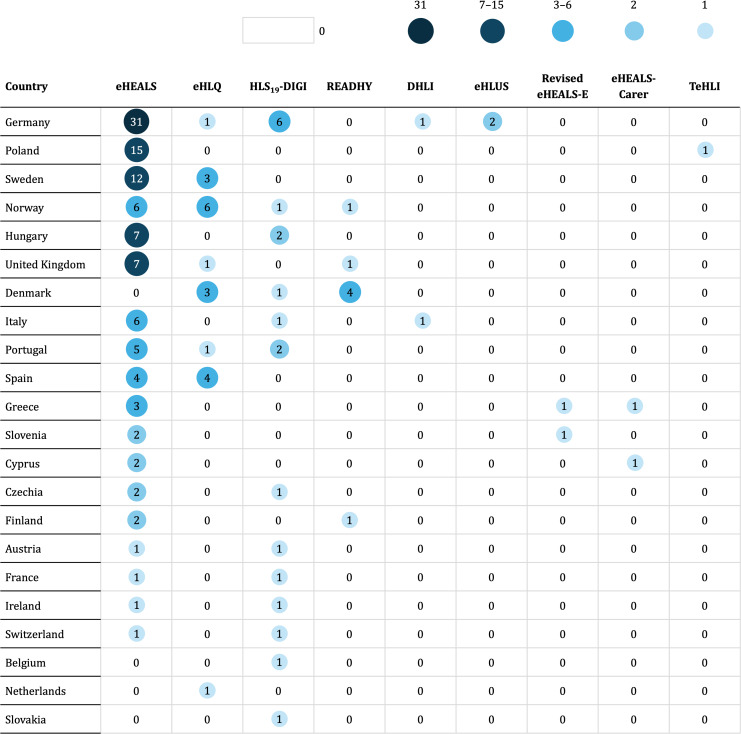
eHealth literacy measurement instruments by country of data collection identified in the included papers (N=132). One multicountry paper also included data from Israel, which lies outside the target region of this study and is therefore not listed [117]. Another paper (1/132, 0.76%) reported 6 countries of data collection and other unspecified countries in the European Union or the European Economic Area, which are therefore not listed [59]. DHLI, Digital Health Literacy Instrument; eHEALS, eHealth Literacy Scale; eHEALS-Carer, eHealth Literacy Scale for Carers of People with Chronic Diseases; eHEALS-E, eHealth Literacy Scale-Extended; eHLQ, eHealth Literacy Questionnaire; eHLUS, eHealth Literacy and Use Scale; HLS_19_-DIGI, digital health literacy module of the Health Literacy Survey 2019‐2021; READHY, Readiness and Enablement Index for Health Technology; TeHLI, Transactional eHealth Literacy Instrument.

The use of the eHL measurement instruments varied considerably by country. Versions of the eHEALS were used across 19 different countries within the target regions of this study. In contrast, the other identified eHL measurement instruments were used much less frequently. Apart from the eHEALS, only the eHLQ, the HLS_19_-DIGI, and the READHY were each used in more than 2 countries. The distribution of the eHL measurement instruments by country of data collection in the included papers is shown in Figure 3.

##### Languages

As shown in Table 4, the described studies collected data within the target regions of this paper in a total of 22 languages, most frequently in German (41/132, 31.06%). Two of the 132 included papers (1.52%) reported data collection exclusively in a language other than the official language of the country of data collection (Sweden), namely Arabic [66,182]. Ten of the 132 included papers (7.58%) described studies that collected data in 2 or more languages, including 1 paper (1/132, 0.76%) that reported data collection in both the country’s official language (Spanish) and a regional official language (Catalan) [97], 2 papers (2/132, 1.52%) that described multicountry studies in which data were collected exclusively in the official language(s) of the respective countries within the target regions of this study [117,143] (one of which also reported data collection in nonofficial languages in Israel [117], which lies outside the predefined target regions of this study), and 7 papers (7/132, 5.30%) that reported data collection not only in the official language(s) of the respective countries within the target regions of this study but also in other languages—English (4/132, 3.03%) [58,59,153,179]; Arabic (1/132, 0.76%) [65]; Russian and Turkish (1/132, 0.76%) [152]; and Arabic, English, Russian, Turkish, and Twi (1/132, 0.76%) [128].

**Table 4.** Data collection languages identified in the included papers (N=132).^a^

Language	Papers, n (%)	Citations
German	41 (31.06)	[58,59,62,70,74,76,77,87,91,92,99,100,104,106,108,109,111,113,114,117,120,123-125,128,130,136,137,143,147,150-154,156,164,166,174,183,184]
English	16 (12.12)	[58,59,115-117,121,127-129,143,144,153,162,173,178,179]
Polish	15 (11.36)	[72,78-84,107,134,135,160,165,187,188]
Norwegian	14 (10.61)	[61,64,71,75,96,103,117,118,126,132,133,145,146,172]
Swedish	13 (9.85)	[65,67,93,94,112,143,157-159,167,168,176,181]
Hungarian	9 (6.82)	[58,59,69,101,102,117,138,185,186]
Italian	9 (6.82)	[68,117,119,139,143,149,155,171,180]
Danish	8 (6.06)	[63,117,148,161,163,169,170,179]
Portuguese	8 (6.06)	[58-60,90,117,122,131,177]
Greek	6 (4.54)	[58,59,85,86,105,110]
Spanish	6 (4.54)	[58,59,88,89,97,98]
Arabic	4 (3.03)	[65,66,128,182]
Czech	3 (2.27)	[117,140,141]
Finnish	3 (2.27)	[58,95,175]
Slovenian	3 (2.27)	[22,58,59]
Dutch	2 (1.52)	[117,142]
French	2 (1.52)	[73,117]
Russian	2 (1.52)	[128,152]
Turkish	2 (1.52)	[128,152]
Catalan	1 (0.76)	[97]
Slovak	1 (0.76)	[117]
Twi	1 (0.76)	[128]

a Ten included papers (10/132, 7.58%) are listed 2 or more times because they reported data collection in multiple languages [58,59,65,97,117,128,143,152,153,179]. For 1 paper describing a multicountry study that also included participants from Israel, only the data collection languages used within the predefined target regions of this study are shown [117].

### Samples

#### Health-Related Perspectives

Three major clusters were identified regarding the health-related perspectives of the target groups:

1. Sixty-four of the 132 included papers (48.48%) reported that patients or individuals with current or previous health conditions were the target group [61-64,70,71,74,75,87-89,91,93,95,96,99,100,103,106,108,110,111,114-116,118,120-122,124-127,129,130,136,144-149,153,156-159,161-165,167-171,174,177,179,180,183-185].
2. Eleven of the 132 included papers (8.33%) stated that parents or legal guardians of children or informal caregivers were the target group [85,101,104,112,113,132,133,135,158,172,176].
3. Fifty-nine of the 132 included papers (44.70%) described studies with other target groups (eg, general population or students) [22,58-60,65-69,72,73,76-84,86,90,92,94,97,98,102,105,107,109,117,119,123,128,131,134,137-143,150-152,154,155,160,166,173,175,178,181,182,184,186-188].

Two of the 132 included papers (1.52%) were each assigned to 2 of these clusters. Of these, one study (1/132, 0.76%) reported that patients (assigned to cluster 1) and parents of hospitalized children (assigned to cluster 2) were the target group [158], and the other (1/132, 0.76%) reported that users of symptom checker applications (assigned to cluster 1) and nonusers of symptom checker applications (assigned to cluster 3) were the target group [184]. One of the included papers (1/132, 0.76%) reported that, in addition to patients with hypertension (assigned to cluster 1), physicians were the target group [70]; no target group cluster 4 for physicians was created, as this study did not focus on individuals with a professional perspective.

#### Age Groups

In terms of age groups, the included papers were grouped into three clusters:

1. Of the 132 included papers, 119 (90.15%) reported that only adults were included. Of these papers, 104 (104/132, 78.79%) stated that adults in general aged ≥18 or >18 years or broad adult age ranges were included [22,58,59,61,62,65-67,69-71,75,77-79,81-90,92,93,95-101,103,105-108,110-121,123-131,133,136-139,142-153,155-160,162-167,170-174,177,178,180-185,187,188]. Furthermore, 10 of the 132 papers (7.58%) focused on older adults, namely adults aged ≥40 years (2/132, 1.52%) [102,186], >45 years (1/132, 0.76%) [63], ≥50 years (1/132, 0.76%) [68], 50 to 69 years (1/132, 0.76%) [161], ≥60 years (1/132, 0.76%) [74], and ≥65 years (4/132, 3.03%) [64,94,122,169]. Two papers (2/132, 1.52%) mentioned that younger adults were included, namely adults aged 18 to 29 years (1/132, 0.76%) [175] and 18 to 35 years (1/132, 0.76%) [80]; 2 papers (2/132, 1.52%) reported that middle-aged adults were included, namely adults aged 30 to 65 years (1/132, 0.76%) [168] and 34 to 64 years (1/132, 0.76%) [91]; and 1 paper (1/132, 0.76%) reported that both “younger adults” and “older adults” were included, without further specification ([134], p. 1739).
2. Five of the 132 included papers (3.79%) reported that minor and adult participants were included. Of these, 1 paper (1/132, 0.76%) mentioned that individuals with a verified social media account were the target group, regardless of their age, with the actual age range being 14 to 72 years [72]; 3 papers (3/132, 2.27%) specified that individuals aged ≥14 years (1/132, 0.76%) [76] and ≥16 years (2/132, 1.52%) [109,154] were included; and 1 paper (1/132, 0.76%) stated that individuals aged <35 years were included [73].
3. Eight of the 132 included papers (6.06%) did not provide a target age, a qualitative description regarding the age, an actual age range, or age groups [60,104,132,135,140,141,176,179].

#### Sample Sizes

As shown in Table 5, the included papers were divided into 7 clusters on the basis of the sample sizes reported for the central eHL measurement(s), or total sample if not available, ranging from <50 (7/132, 5.30%) to >2000 participants (8/132, 6.06%).

**Table 5.** Sample sizes reported for the central eHealth literacy measurement(s) or, if unavailable, the overall study sample sizes in the included papers (N=132).

Sample size, n	Papers, n (%)	Citations
<50	7 (5.30)	[74,91,95,112,113,121,169]
51‐100	12 (9.09)	[58,59,63,68,128,136,148,157,161,163,165,177]
101‐250	36 (27.27)	[64,70,75,85,87-90,99-101,103,106,108,110,111,114,118,131-133,144,147,156,158,159,162,164,166,168,170,171,174-176,184]
251‐500	30 (22.73)	[61,62,66,67,73,86,92,94,96,98,115,116,120,122-124,126,130,134,138,146,153,155,167,172,178-182]
501‐1000	20 (15.15)	[60,65,69,76-78,93,97,104,105,129,135,137,139-141,154,183,185,186]
1001‐2000	19 (14.39)	[22,71,72,79,80,83,84,102,107,109,119,125,127,142,145,149,151,152,160]
>2000	8 (6.06)	[81,82,117,143,150,173,187,188]

## Discussion

This paper aimed to provide an overview of how eHL has been assessed in the European Economic Area, Switzerland, and the United Kingdom in recent years and which methodological limitations need to be considered.

### Principal Findings

#### Summary of Evidence

A total of 132 empirical papers were included in the final analysis. The publication trend indicated growing research activity in the field of eHL measurement. Across the included papers, the use of versions of 9 eHL measurement instruments that provide eHL scores based on self-reports was reported, most frequently the use of the eHEALS (94/132, 71.21%). All included papers (132/132, 100%) reported the use of surveys to collect data on eHL, most of which were conducted digitally (71/132, 53.79%). Reported limitations include potential self-report biases associated with the use of self-report instruments (37/132, 28.03%), selection biases due to the data collection mode (26/132, 19.70%), and limitations specific to 5 of the 9 identified eHL measurement instruments (18/132, 13.64%). Most included papers (121/132, 91.67%) reported eHL results from a single measurement per participant. Data were collected across 22 countries within the target regions, most frequently in Germany (39/132, 29.54%). The use of the eHL measurement instruments varied considerably between the countries. In total, 22 different data collection languages were identified, with German being the most common (41/132, 31.06%). Patients or individuals with current or previous health problems were a commonly reported target group (64/132, 48.48%). Broad adult age ranges were very common (104/132, 78.79%). Sample sizes ranged from ≤50 (7/132, 5.30%) to >2000 (8/132, 6.06%) participants.

#### Self-Report eHealth Literacy Measurement Instruments

Self-report biases are a key limitation of self-report measurement instruments [198,199], as also noted in various papers included in this scoping review. A study, which was also cited in some included papers, found a weak relationship between the eHEALS—the most frequently used eHL measurement instrument—and performance-based task [14,23]. However, no performance-based eHL measurement instruments were identified via this scoping review. One reason may be that we did not consider instruments solely assessing only some of the skills described in the 2006 definition of eHL by Norman and Skinner [13] or focusing on specific subdimensions of eHL. Crocker et al ([18], p. 1) identified “29 unique performance-based eHealth literacy measurement tools,” using less strict criteria. One of these was used in a study also included in this scoping review: Schulz et al [155] reported the use of the eHEALS alongside additional tasks. Specifically, participants rated the quality of 2 simulated websites (1 with high quality and 1 with low quality) using a 7-point semantic differential scale (eg, “accurate” vs “inaccurate”) ([155], p. 4); furthermore, they were shown the right treatment options (eg, “seeking help from a doctor”) ([155], p. 4]) and wrong treatment options (eg, “treating depression with St John’s wort or vitamins or yoga without any mention of antidepressant medications or psychotherapy”) ([155], p. 4]) and had to make a selection. While Crocker et al [18] considered this approach an eHL measurement instrument, it does not capture the relevant aspects of the eHL definition by Norman and Skinner [13] (eg, searching health information) and was therefore not considered an eHL measurement instrument in this paper. Another instrument, identified by Crocker et al [18] and via this scoping review, is the DHLI [14]. It comprises 21 self-report items intended for calculating eHL scores and 7 supplementary performance-based items, which did not demonstrate acceptable validity in the validation study [14]. Thus, the DHLI [14] is a self-report eHL measurement instrument with supplementary performance-based items rather than a performance-based eHL measurement instrument. Nevertheless, Crocker et al [18] found that performance-based eHL measurement instruments are rarely used and highlighted that several were not completely published. They also mention other challenges of performance-based eHL measurement instruments, such as the requirement of regular updates considering new scientific evidence, varying topic relevance for different target groups, and the time demand for researchers and participants [18].

#### Exclusion of Subgroups in Online Surveys

Selection biases were mentioned in the included papers, particularly in the context of frequent use of online surveys. Online surveys potentially introduce self-selection biases and lead to an undercoverage of subgroups, such as individuals with limited or no access to the internet [200]. Despite growing internet adoption, 6% of households in the European Union still had no internet access in 2024 [201]. Hardware and internet access, in particular, are prerequisites for using digital tools [202]. However, even individuals who do not yet use the internet may benefit from digital health interventions if they are equipped with the necessary hardware and an internet connection. In addition, digital interventions need to reflect eHL-related needs [203]. Therefore, it would be important to ensure inclusion of subgroups who are not yet using the internet, and other potentially underrepresented subgroups, which were mentioned in the included papers. Selection biases can be addressed through methodological strategies, such as probability sampling [200]. Another approach is mixed-mode designs (ie, different modes are offered to different target groups)—which, however, introduces further (selection) biases [204]—as well as sequential mixed-mode designs (ie, different survey modes are offered to the same target group one after another) [205].

#### Alignment With Technological Innovations

This study found that some included papers criticized eHL measurement instruments for not fully aligning with technological developments and shifting use cases. As the internet has been getting more interactive since the eHEALS was published in 2006, skills related to social media, where users potentially encounter health misinformation [10], are considered relevant in the context of eHL [23,24]. Although social media is not a new phenomenon, only some identified eHL measurement instruments contain items explicitly related to interactive digital tools: the revised eHEALS-E [22], the TeHLI [196], and the DHLI [14]. Also, the HLS_19_-DIGI [189], which is based on the DHLI [14], contains such items, although not in the main part intended for calculating eHL scores (HL-DIGI). eHL can also be considered essential in the context of health information provided by GenAI, as studies [11,12] show that health misinformation is a serious issue. Yet, GenAI has been established in recent years, and this may explain why it is not explicitly mentioned in older eHL measurement instruments. However, not even the revised eHEALS-E [22] and the eHLUS [166] published in the period after the launch of ChatGPT in 2022 mention GenAI tools explicitly. However, the developers in the revised eHEALS-E took GenAI into account, pointing out “that the Being smart on the Net dimension is particularly important in the context of the rapidly evolving internet technologies based on artificial intelligence algorithms that require critical awareness, distance, and understanding” ([22], p. 8). Dedicated measurement instruments were developed in recent years to measure artificial intelligence literacy in general [206,207]. Furthermore, as outlined, newer theoretical models such as the eHLF [26] and the new Lily model [28] also include the aspect “safety.” However, safety in terms of data protection is only considered in the DHLI [14], the eHLF-based eHLQ [27], and the eHLUS [166]. Overall, the findings indicate the need to continuously review and update eHL measurement instruments to ensure alignment with technological innovations. Developing an eHL measurement instrument that does not become obsolete in a rapidly changing digital environment can be described as a key challenge for future research.

#### Complexity of eHealth Literacy Measurement Instruments

In terms of complexity or length, the eHEALS-E [22], the eHLQ [27], and the READHY [190], which contains the eHLQ [27], were criticized in the included papers. Similar to the DHLI [14], the TeHLI [196], and the eHLUS [166], these eHL measurement instruments capture a broader range of dimensions of eHL. Efthymiou et al [86], who validated the Greek versions of the eHEALS and the revised eHEALS-E, provided guidance in selecting the appropriate eHL measurement instrument. Specifically, they noted that “[t]he eHeals could be used as an additional tool when eHealth Literacy is not the core concept measured and the revised eHeals-Extended can be used when researchers wish to measure eHealth Literacy concept more thoroughly” ([86], p. 2). Following this argumentation, the use of eHL measurement instruments with a broader range of dimensions should be considered when the aim is to generate deeper insights, including specific needs of individuals. However, it is debatable whether health professionals will follow this advice. For example, Norman ([24], p. 2) noted that the eHEALS was developed as a short scale, as “health professionals […] said they would not use a long instrument in practice.”

#### eHealth Literacy Measurement Instruments for Older Adults

In addition, we want to highlight that none of the eHL measurement instruments identified in this paper was specifically developed for older adults. Also, Wang et al [208] noted a lack of such eHL measurement instruments. The researchers recently introduced the Digital Health Literacy Questionnaire for Older Adults “designed to align with the realities of older adults’ daily lives and health care experiences, focusing on potential challenges they may face when using digital technologies to access, process, communicate, and understand health information and services“ ([208], p. 12). The instrument is not included in the final analysis of this scoping review, as it was used to assess eHL of older adults in China [208]. Older age groups differ from younger individuals in certain characteristics (eg, physical and cognitive conditions, literacy levels, technology usage, and privacy preferences), requiring special methodological considerations, for example, in terms of item complexity [209]. As older adults are particularly at risk of digital exclusion [9,210], there is a need to evaluate the appropriateness of eHL measurement instruments for them.

### Limitations

The review process used is subject to some limitations. First, it needs to be noted that, due to the use of ASReview to facilitate the title and abstract screening of the English records, 30.77% (2523/8199) of the English records from the initial searches and 34.93% (1115/3192) of the English records from the updated searches were screened. The remaining English records remained after the ASReview stopping criterion (ie, 100 consecutive irrelevant records) was reached in both cases. Thus, despite the demonstrated performance of ASReview [40], there is a risk that relevant papers were not screened and thus not included in the final analysis.

Second, the exclusion of papers without a clear focus on the target regions resulted in the omission of papers and eHL measurement instruments. As German and English papers were included, there may be an overrepresentation of papers from German- and English-speaking regions and an underrepresentation of papers from non–German-speaking and non–English-speaking regions within the target regions.

Third, the analysis covered limitations reported in the *Limitations* section or, if a *Limitations* section was unavailable, the *Discussion* section of the included papers. Therefore, limitations reported elsewhere in the included papers were not analyzed. Moreover, the analysis was limited to reported limitations regarding eHL measurement instruments as well as eHL-related data collection methods and modes. Other limitations reported in the included papers (eg, those related to cross-sectional study designs or sampling or recruitment strategies and limitations specific to translated and culturally adapted versions of eHL measurement instruments) were not extracted and analyzed in this study.

### Implications for Practice, Future Research, and Policy

This paper provides health care practitioners and researchers with a structured overview of limitations related to eHL measurement instruments and data collection methods and modes. The identified eHL measurement instruments differ in key aspects, including length, complexity, and alignment with rapidly evolving digital use cases. Particularly, online surveys might lead to an exclusion of subgroups. Thus, it clearly shows that selecting both a suitable eHL measurement instrument and suitable data collection methods and modes is a key decision, which should be based on factors such as the purpose of the planned eHL measurement, the target population, and the data collection setting.

The overview could encourage researchers to adopt eHL measurements that are less common or were not used in specific countries yet. This potentially requires translation, cultural adaptation, and validation efforts. Future research should also focus on revising existing eHL measurement instruments and developing new eHL measurement instruments to capture the skills required for an ever-growing range of digital tools in the context of health, including social media and GenAI. This may require new underlying operationalization approaches that manage to avoid becoming obsolete. Use case–specific eHL measurement instruments may also be relevant for health care providers and researchers who only want to assess specific aspects (eg, skills that are necessary when using a specific digital health application) considering setting and time constraints (eg, in health care facilities). The fact that only self-report eHL measurement instruments were identified and the associated risk of self-report biases reported in the included papers indicate a need for performance-based eHL measurement approaches. Furthermore, revised or new eHL measurement instruments tailored to older adults are highly relevant for gaining a better understanding of the specific eHL-related needs.

Policymakers should encourage health care providers to conduct eHL measurements using carefully selected eHL measurement instruments and data collection methods and modes to provide targeted solutions based on individual eHL-related needs.

### Conclusions

To our knowledge, this is the first scoping review synthesizing eHL measurement limitations reported in empirical papers from the European Economic Area, Switzerland, and the United Kingdom. The analysis of 132 empirical papers showed that eHL was assessed using 9 eHL measurement instruments in various languages, especially the eHEALS, and that digital surveys were a common data collection approach. The identified eHL measurement–related limitations, such as self-reported eHL measurement, exclusion of subgroups, and lack of alignment of technological developments and changing use cases in the context of health, potentially lead to biased results. Therefore, selecting suitable eHL measurement approaches is a critical decision. Health care professionals and researchers should consider a variety of factors, such as measurement purpose, target population, and data collection setting. There is a need for translated and culturally adapted as well as revised or newly developed eHL measurement instruments that keep pace with technological change, cover diverse use cases or focus on specific use cases, and contain performance-based components. Policymakers should encourage health care providers to thoughtfully select appropriate eHL measurement approaches and use eHL-related insights as a basis for targeted solutions.

## Supplementary material

10.2196/87461 Multimedia Appendix 1 Inclusion and exclusion criteria.

10.2196/87461 Multimedia Appendix 2 Search strings, filters, and expanders.

10.2196/87461 Multimedia Appendix 3 Metadata of the included papers.

10.2196/87461 Multimedia Appendix 4 Characteristics of the identified original eHealth literacy measurement instruments.

10.2196/87461 Multimedia Appendix 5 Characteristics of the eHealth literacy measurement instruments validated in the included papers.

10.2196/87461 Checklist 1 Preferred Reporting Items for Systematic reviews and Meta-Analyses Extension for Scoping Reviews (PRISMA-ScR) Checklist.

## Abbreviations

DHLI: Digital Health Literacy Instrument
DiGA: Digital Health Applications (Digitale Gesundheitsanwendungen)
DVG: Digital Care Act (Digitale-Versorgung-Gesetz)
eHEALS: eHealth Literacy Scale
eHEALS-Carer: eHealth Literacy Scale for Carers of People with Chronic Diseases
eHEALS-E: eHealth Literacy Scale-Extended
eHL: eHealth literacy
eHLF: eHealth Literacy Framework
eHLQ: eHealth Literacy Questionnaire
eHLUS: eHealth Literacy and Use Scale
GDPR: General Data Protection Regulation
GenAI: generative artificial intelligence
HLS_19_-DIGI: digital health literacy module of the Health Literacy Survey 2019‐2021
PCC: population, concept, and context framework
PRISMA-S: Preferred Reporting Items for Systematic Reviews and Meta-Analyses Literature Search Extension
PRISMA-ScR: Preferred Reporting Items for Systematic Reviews and Meta-Analyses Extension for Scoping Reviews
READHY: Readiness and Enablement Index for Health Technology
TeHLI: Transactional eHealth Literacy Instrument
TF-IDF: term frequency-inverse document frequency

## References

[1] (2025). Individuals using the internet for seeking health-related information. https://ec.europa.eu/eurostat/databrowser/view/tin00101/default/table.

[2] Bautista JR, Herbert D, Farmer M, De Torres RQ, Soriano GP, Ronquillo CE (2025). Health consumers' use and perceptions of health information from generative artificial intelligence chatbots: a scoping review. Appl Clin Inform.

[3] Chen J, Wang Y (2021). Social media use for health purposes: systematic review. J Med Internet Res.

[4] (2025). Apple Health: meaningful insights: backed by science. Apple.

[5] (2025). Google Fit: coaching you to a healthier and more active life. Google.

[6] (2023). Classification of Digital Interventions, Services and Applications in Health: A Shared Language to Describe the Uses of Digital Technology for Health.

[7] Livieri G, Mangina E, Protopapadakis ED, Panayiotou AG (2025). The gaps and challenges in digital health technology use as perceived by patients: a scoping review and narrative meta-synthesis. Front Digit Health.

[8] Friemel TN (2016). The digital divide has grown old: determinants of a digital divide among seniors. New Media Soc.

[9] Ge H, Li J, Hu H, Feng T, Wu X (2025). Digital exclusion in older adults: a scoping review. Int J Nurs Stud.

[10] Suarez-Lledo V, Alvarez-Galvez J (2021). Prevalence of health misinformation on social media: systematic review. J Med Internet Res.

[11] Ayers JW, Zhu Z, Poliak A (2023). Evaluating artificial intelligence responses to public health questions. JAMA Netw Open.

[12] Andrikyan W, Sametinger SM, Kosfeld F (2025). Artificial intelligence-powered chatbots in search engines: a cross-sectional study on the quality and risks of drug information for patients. BMJ Qual Saf.

[13] Norman CD, Skinner HA (2006). eHealth literacy: essential skills for consumer health in a networked world. J Med Internet Res.

[14] van der Vaart R, Drossaert C (2017). Development of the digital health literacy instrument: measuring a broad spectrum of Health 1.0 and Health 2.0 skills. J Med Internet Res.

[15] Gilstad H (2014). Proceedings of the 2nd European Workshop on Practical Aspects of Health Informatics (PAHI 2014).

[16] Griebel L, Enwald H, Gilstad H, Pohl AL, Moreland J, Sedlmayr M (2018). eHealth literacy research: quo vadis?. Inform Health Soc Care.

[17] Norman CD, Skinner HA (2006). eHEALS: the eHealth Literacy Scale. J Med Internet Res.

[18] Crocker B, Feng O, Duncan LR (2023). Performance-based measurement of eHealth literacy: systematic scoping review. J Med Internet Res.

[19] Karnoe A, Kayser L (2015). How is eHealth literacy measured and what do the measurements tell us? A systematic review. Knowl Manag E-Learn.

[20] Wang C, Chang L, Chen X, Kong J, Qi H (2025). eHealth literacy assessment instruments: scoping review. J Med Internet Res.

[21] Petrič G, Atanasova S, Kamin T (2017). Ill literates or illiterates? Investigating the eHealth literacy of users of online health communities. J Med Internet Res.

[22] Petrič G, Atanasova S (2024). Validation of the extended e-health literacy scale: structural validity, construct validity and measurement invariance. BMC Public Health.

[23] van der Vaart R, van Deursen AJ, Drossaert CH, Taal E, van Dijk JA, van de Laar MA (2011). Does the eHealth Literacy Scale (eHEALS) measure what it intends to measure? Validation of a Dutch version of the eHEALS in two adult populations. J Med Internet Res.

[24] Norman C (2011). eHealth literacy 2.0: problems and opportunities with an evolving concept. J Med Internet Res.

[25] Levin-Zamir D, Bertschi I (2018). Media health literacy, eHealth literacy, and the role of the social environment in context. Int J Environ Res Public Health.

[26] Norgaard O, Furstrand D, Klokker L (2015). The e-health literacy framework: a conceptual framework for characterizing e-health users and their interaction with e-health systems. Knowl Manag E-Learn.

[27] Kayser L, Karnoe A, Furstrand D (2018). A multidimensional tool based on the eHealth literacy framework: development and initial validity testing of the eHealth Literacy Questionnaire (eHLQ). J Med Internet Res.

[28] Milanti A, Norman C, Chan DNS, So WKW, Skinner H (2025). eHealth literacy 3.0: updating the Norman and Skinner 2006 model. J Med Internet Res.

[29] Hsia J, Zhao G, Town M (2020). Estimating undercoverage bias of internet users. Prev Chronic Dis.

[30] Tricco AC, Lillie E, Zarin W (2018). PRISMA extension for scoping reviews (PRISMA-ScR): checklist and explanation. Ann Intern Med.

[31] (2019). Gesetz für eine bessere Versorgung durch Digitalisierung und Innovation [Article in German]. Bundesgesetzblatt.

[32] van Kessel R, Srivastava D, Kyriopoulos I (2023). Digital health reimbursement strategies of 8 European countries and Israel: scoping review and policy mapping. JMIR Mhealth Uhealth.

[33] Tarricone R, Petracca F, Weller HM (2024). Towards harmonizing assessment and reimbursement of digital medical devices in the EU through mutual learning. NPJ Digit Med.

[34] Legal framework of EU data protection. European Commission.

[35] (2020). Federal Act on Data Protection (Data Protection Act, FADP). Fedlex: the publication platform for federal law.

[36] (2026). Data protection. GOV.UK.

[37] Rethlefsen ML, Kirtley S, Waffenschmidt S (2021). PRISMA-S: an extension to the PRISMA Statement for Reporting Literature Searches in Systematic Reviews. Syst Rev.

[38] Gusenbauer M, Haddaway NR (2020). Which academic search systems are suitable for systematic reviews or meta-analyses? Evaluating retrieval qualities of Google Scholar, PubMed, and 26 other resources. Res Synth Methods.

[39] Pollock D, Peters MDJ, Khalil H (2023). Recommendations for the extraction, analysis, and presentation of results in scoping reviews. JBI Evid Synth.

[40] van de Schoot R, de Bruin J, Schram R (2021). An open source machine learning framework for efficient and transparent systematic reviews. Nat Mach Intell.

[41] Ouzzani M, Hammady H, Fedorowicz Z, Elmagarmid A (2016). Rayyan: a web and mobile app for systematic reviews. Syst Rev.

[42] Adamse I, Eichelsheim V, Blokland A, Schoonmade L (2024). The risk and protective factors for entering organized crime groups and their association with different entering mechanisms: a systematic review using ASReview. Eur J Criminol.

[43] van den Berg RL, van der Landen SM, Keijzer MJ (2025). Smartphone- and tablet-based tools to assess cognition in individuals with preclinical Alzheimer disease and mild cognitive impairment: scoping review. J Med Internet Res.

[44] van Dijk SHB, Brusse-Keizer MGJ, Bucsán CC, van der Palen J, Doggen CJM, Lenferink A (2023). Artificial intelligence in systematic reviews: promising when appropriately used. BMJ Open.

[45] Álvarez-Pérez Y, Perestelo-Pérez L, Rivero-Santanta A (2022). Co-creation of massive open online courses to improve digital health literacy in pregnant and lactating women. Int J Environ Res Public Health.

[46] Martens L, Brand T, Zeeb H, Graff H, Wiersing R, Muellmann S (2025). Digitale Gesundheitsinformationen und -angebote: Eine Fokusgruppenstudie zu Nutzungsbarrieren mit digital exkludierten Bevölkerungsgruppen [Article in German]. Präv Gesundheitsf.

[47] Schulz PJ, Rothenfluh F (2020). Influence of health literacy on effects of patient rating websites: survey study using a hypothetical situation and fictitious doctors. J Med Internet Res.

[48] Zangger G, Mortensen SR, Tang LH, Thygesen LC, Skou ST (2024). Association between digital health literacy and physical activity levels among individuals with and without long-term health conditions: data from a cross-sectional survey of 19,231 individuals. Digit Health.

[49] Bak CK, Krammer JØ, Dadaczynski K (2022). Digital health literacy and information-seeking behavior among university college students during the COVID-19 pandemic: a cross-sectional study from Denmark. Int J Environ Res Public Health.

[50] Dadaczynski K, Okan O, Messer M (2021). Digital health literacy and web-based information-seeking behaviors of university students in Germany during the COVID-19 pandemic: cross-sectional survey study. J Med Internet Res.

[51] Lorini C, Velasco V, Bonaccorsi G (2022). Validation of the COVID-19 digital health literacy instrument in the Italian language: a cross-sectional study of Italian university students. Int J Environ Res Public Health.

[52] Martins S, Augusto C, Martins MRO (2022). Adaptation and validation of the Digital Health Literacy Instrument for Portuguese university students. Health Promot J Austr.

[53] Rosário R, Fronteira I, Martins MRO (2022). Infodemic preparedness and COVID-19: searching about public health and social measures is associated with digital health literacy in university students. Int J Environ Res Public Health.

[54] Bonaccorsi G, Gallinoro V, Guida A (2023). Digital health literacy and information-seeking in the era of COVID-19: gender differences emerged from a Florentine University experience. Int J Environ Res Public Health.

[55] Castarlenas E, Sánchez-Rodríguez E, Roy R (2021). Electronic health literacy in individuals with chronic pain and its association with psychological function. Int J Environ Res Public Health.

[56] Page MJ, McKenzie JE, Bossuyt PM (2021). The PRISMA 2020 statement: an updated guideline for reporting systematic reviews. BMJ.

[57] Attribution 4.0 International (CC BY 4.0). Creative Commons.

[58] Ahmed F, Romero Saletti S, D’Souza E (2025). Assessing the user experience of the EU mobile app for cancer prevention: mixed methods study. JMIR Form Res.

[59] Ahmed F, Romero Saletti SM, D’Souza E (2025). Piloting a cancer awareness app across six European countries: a pre-post study. Front Public Health.

[60] Almeida S, Pinto E, Correia M, Veiga N, Almeida A (2024). Evaluating e-Health literacy, knowledge, attitude, and health online information in Portuguese university students: a cross-sectional study. Int J Environ Res Public Health.

[61] Andersen MH, Hermansen Å, Dahl KG (2024). Profiles of health literacy and digital health literacy in clusters of hospitalised patients: a single-centre, cross-sectional study. BMJ Open.

[62] Bäuerle A, Marsall M, Jahre LM (2023). Psychometric properties of the German revised version of the eHealth literacy scale in individuals with cardiac diseases: validation and test of measurement invariance. Digit Health.

[63] Bendtsen MG, Schönwandt BMT, Rubæk M, Hitz MF (2024). Evaluation of an mHealth app on self-management of osteoporosis: prospective survey study. Interact J Med Res.

[64] Bergh S, Benth JŠ, Høgset LD, Rydjord B, Kayser L (2025). Assessment of technology readiness in Norwegian older adults with long-term health conditions receiving home care services: cross-sectional questionnaire study. JMIR Aging.

[65] Bergman L, Nilsson U, Dahlberg K, Jaensson M, Wångdahl J (2021). Health literacy and e-Health literacy among Arabic-speaking migrants in Sweden: a cross-sectional study. BMC Public Health.

[66] Bergman L, Nilsson U, Dahlberg K, Jaensson M, Wångdahl J (2023). Validity and reliability of the Arabic version of the HLS-EU-Q16 and HLS-EU-Q6 questionnaires. BMC Public Health.

[67] Bergman L, Nilsson U, Dahlberg K, Jaensson M, Wångdahl J (2023). Validity and reliability of the Swedish versions of the HLS-EU-Q16 and HLS-EU-Q6 questionnaires. BMC Public Health.

[68] Bevilacqua R, Strano S, Di Rosa M (2021). eHealth literacy: from theory to clinical application for digital health improvement: results from the ACCESS training experience. Int J Environ Res Public Health.

[69] Bíró É, Vincze F, Nagy-Pénzes G, Ádány R (2023). Investigation of the relationship of general and digital health literacy with various health-related outcomes. Front Public Health.

[70] Breil B, Salewski C, Apolinário-Hagen J (2022). Comparing the acceptance of mobile hypertension apps for disease management among patients versus clinical use among physicians: cross-sectional survey. JMIR Cardio.

[71] Brørs G, Wentzel-Larsen T, Dalen H (2020). Psychometric properties of the Norwegian version of the electronic Health Literacy Scale (eHEALS) among patients after percutaneous coronary intervention: cross-sectional validation study. J Med Internet Res.

[72] Burzyńska J, Rękas M, Januszewicz P (2022). Evaluating the psychometric properties of the eHealth Literacy Scale (eHEALS) among Polish social media users. Int J Environ Res Public Health.

[73] Chaniaud N, Sagnier C, Loup-Escande E (2022). Translation and validation study of the French version of the eHealth Literacy Scale: web-based survey on a student population. JMIR Form Res.

[74] Chatsatrian M, Kunde K, Bosompem J (2025). Usability evaluation of digital health applications for older people with depressive disorders: prospective observational study in a mixed methods design. JMIR Hum Factors.

[75] Dale JG, Lüthi A, Fundingsland Skaraas B, Rundereim T, Dale B (2020). Testing measurement properties of the Norwegian version of Electronic Health Literacy Scale (eHEALS) in a group of day surgery patients. J Multidiscip Healthc.

[76] De Santis KK, Jahnel T, Sina E, Wienert J, Zeeb H (2021). Digitization and health in Germany: cross-sectional nationwide survey. JMIR Public Health Surveill.

[77] De Santis KK, Muellmann S, Pan CC (2024). Digitisation and health: second nationwide survey of internet users in Germany. Digit Health.

[78] Duplaga M (2020). The acceptance of key public health interventions by the Polish population is related to health literacy, but not eHealth literacy. Int J Environ Res Public Health.

[79] Duplaga M (2020). The determinants of conspiracy beliefs related to the COVID-19 pandemic in a nationally representative sample of internet users. Int J Environ Res Public Health.

[80] Duplaga M (2020). The use of fitness influencers’ websites by young adult women: a cross-sectional study. Int J Environ Res Public Health.

[81] Duplaga M (2022). A nationwide natural experiment of e-health implementation during the COVID-19 pandemic in Poland: user satisfaction and the ease-of-use of remote physician’s visits. Int J Environ Res Public Health.

[82] Duplaga M (2022). The roles of health and e-health literacy, conspiracy beliefs and political sympathy in the adherence to preventive measures recommended during the pandemic. Int J Environ Res Public Health.

[83] Duplaga M, Grysztar M (2021). The association between future anxiety, health literacy and the perception of the COVID-19 pandemic: a cross-sectional study. Healthcare (Basel).

[84] Duplaga M, Turosz N (2022). User satisfaction and the readiness-to-use e-health applications in the future in Polish society in the early phase of the COVID-19 pandemic: A cross-sectional study. Int J Med Inform.

[85] Efthymiou A, Middleton N, Charalambous A, Papastavrou E (2022). Health literacy and eHealth literacy and their association with other caring concepts among carers of people with dementia: a descriptive correlational study. Health Soc Care Community.

[86] Efthymiou A, Kalaitzaki A, Rovithis M, Petrič G (2025). Validation of the eHealth literacy scales: comparison between the shorter and longer versions. Inform Health Soc Care.

[87] Fugmann D, Holsteg S, Schäfer R, Niegisch G, Dinger U, Karger A (2025). Electronic health literacy, psychological distress, and quality of life in urological cancer patients: a longitudinal study during transition from inpatient to outpatient care. Curr Oncol.

[88] García-García D, Ajejas Bazán MJ, Pérez-Rivas FJ (2022). Factors influencing eHealth literacy among Spanish primary healthcare users: cross-sectional study. Int J Environ Res Public Health.

[89] García-García D, Bazán MJA, Pérez-Rivas FJ (2023). Correlation between health and eHealth literacy and a healthy lifestyle: a cross-sectional study of Spanish primary healthcare patients. Healthcare (Basel).

[90] Garcia M, Machado R, Serra I, João AL (2025). eHealth literacy in a migrant community and its association with chronic disease. Front Public Health.

[91] Gehrmann J, Stephan J, Dehner J, Stullich A, Richter M (2025). Pilot study of an app-supported psychosocial prevention intervention: a mixed-methods approach. Pilot Feasibility Stud.

[92] Geiger S, Esser AJ, Marsall M (2024). Association between eHealth literacy and health outcomes in German athletes using the GR-eHEALS questionnaire: a validation and outcome study. BMC Sports Sci Med Rehabil.

[93] Georgsson M, Odzakovic E, Björk M (2025). Validation of the eHealth Literacy Scale instrument in a restless legs syndrome population: classical test theory and Rasch analysis study. J Med Internet Res.

[94] Ghazi SN, Berner J, Anderberg P, Sanmartin Berglund J (2023). The prevalence of eHealth literacy and its relationship with perceived health status and psychological distress during Covid-19: a cross-sectional study of older adults in Blekinge, Sweden. BMC Geriatr.

[95] Giunti G, Yrttiaho T, Guardado-Medina S (2025). Feasibility and usability evaluation of a gamified fatigue management mobile application for persons with multiple sclerosis in everyday life. Mult Scler Relat Disord.

[96] Hermansen Å, Andersen MH, Borge CR (2023). Preliminary validity testing of the eHealth Literacy Questionnaire (eHLQ): a Confirmatory Factor Analysis (CFA) in Norwegian hospitalized patients. BMC Psychol.

[97] Hernández Encuentra E, Robles N, Angulo-Brunet A, Cullen D, Del Arco I (2024). Spanish and Catalan versions of the eHealth Literacy Questionnaire: translation, cross-cultural adaptation, and validation study. J Med Internet Res.

[98] Hernández Encuentra E, González Caballero JL, Montagni I, Fernández Gutiérrez M, Bas Sarmiento P (2025). Digital health literacy among the Spanish population: a descriptive and latent class analysis study. Eur J Public Health.

[99] Holderried M, Hoeper A, Holderried F (2021). Attitude and potential benefits of modern information and communication technology use and telemedicine in cross-sectoral solid organ transplant care. Sci Rep.

[100] Holderried TAW, Stasik I, Schmitz MT (2024). Unleashing the potential of eHealth in outpatient cancer care for patients undergoing immunotherapy: a quantitative study considering patients’ needs and current healthcare challenges. Front Digit Health.

[101] Hölgyesi Á, Luczay A, Tóth-Heyn P (2024). The impact of parental electronic health literacy on disease management and outcomes in pediatric type 1 diabetes mellitus: cross-sectional clinical study. JMIR Pediatr Parent.

[102] Hölgyesi Á, Zrubka Z, Gulácsi L (2024). Robot-assisted surgery and artificial intelligence-based tumour diagnostics: social preferences with a representative cross-sectional survey. BMC Med Inform Decis Mak.

[103] Holmen H, Holm AM, Falk RS (2025). A digital outpatient service with a mobile app for tailored care and health literacy in adults with long-term health service needs: multicenter nonrandomized controlled trial. J Med Internet Res.

[104] Juvalta S, Kerry MJ, Jaks R, Baumann I, Dratva J (2020). Electronic health literacy in Swiss-German parents: cross-sectional study of eHealth literacy scale unidimensionality. J Med Internet Res.

[105] Katsaliaki K (2025). Factors influencing use of eHealth services during and after the COVID-19 pandemic. Health Serv Manage Res.

[106] Knitza J, Simon D, Lambrecht A (2020). Mobile health usage, preferences, barriers, and eHealth literacy in rheumatology: patient survey study. JMIR Mhealth Uhealth.

[107] Kobryn M, Duplaga M (2024). Does health literacy protect against cyberchondria: a cross-sectional study?. Telemed J E Health.

[108] Kokwaro L, Krüger H, Stratmann D (2025). Digital health literacy among people with bipolar disorder in Germany: a cross-sectional survey. Front Psychiatry.

[109] König L, Kuhlmey A, Suhr R (2024). Digital health literacy of the population in Germany and its association with physical health, mental health, life satisfaction, and health behaviors: nationally representative survey study. JMIR Public Health Surveill.

[110] Kostagiolas P, Koumbouli M, Theodorou P, Stamou S (2021). Investigation of the information-seeking behavior of hospitalized patients at the general hospital of Corfu. J Hosp Librariansh.

[111] Kretzschmar C, Knitza J, Pietschner R, Atreya R, Neurath MF, Orlemann T (2025). mHealth use, preferences, barriers, and eHealth literacy among patients with inflammatory bowel disease: survey study. JMIR Hum Factors.

[112] Kristjánsdóttir Ó, Welander Tärneberg A, Stenström P, Castor C, Kristensson Hallström I (2023). eHealth literacy and socioeconomic and demographic characteristics of parents of children needing paediatric surgery in Sweden. Nurs Open.

[113] Kubb C, Foran HM (2022). Online health information seeking for self and child: an experimental study of parental symptom search. JMIR Pediatr Parent.

[114] Lambrecht A, Vuillerme N, Raab C (2021). Quality of a supporting mobile app for rheumatic patients: patient-based assessment using the user version of the Mobile Application Scale (uMARS). Front Med (Lausanne).

[115] Lear R, Freise L, Kybert M, Darzi A, Neves AL, Mayer EK (2022). Patients’ willingness and ability to identify and respond to errors in their personal health records: mixed methods analysis of cross-sectional survey data. J Med Internet Res.

[116] Lear R, Freise L, Kybert M, Darzi A, Neves AL, Mayer EK (2022). Perceptions of quality of care among users of a web-based patient portal: cross-sectional survey analysis. J Med Internet Res.

[117] Levin-Zamir D, Van den Broucke S, Bíró É (2025). Measuring digital health literacy and its associations with determinants and health outcomes in 13 countries. Front Public Health.

[118] Linnestad AM, Skogestad IJ, Gay CL (2025). Exploring digital health literacy clusters in a Norwegian stroke survivor population: a cross-sectional study (NORFAST). Digit Health.

[119] Lo Moro G, Scaioli G, Bert F, Zacchero AL, Minutiello E, Siliquini R (2022). Exploring the the relationship between COVID-19 vaccine refusal and belief in fake news and conspiracy theories: a nationwide cross-sectional study in Italy. Int J Environ Res Public Health.

[120] Lortz J, Rassaf T, Johannsen L (2025). Patient acceptance of video consultations in cardiology. Eur Heart J Digit Health.

[121] Lowe C, Browne M, Marsh W, Morrissey D (2022). Usability testing of a digital assessment routing tool for musculoskeletal disorders: iterative, convergent mixed methods study. J Med Internet Res.

[122] Luz S, Nogueira P, Costa A, Henriques A (2025). Psychometric analysis of the eHealth Literacy Scale in Portuguese older adults (eHEALS-PT24): instrument development and validation. J Med Internet Res.

[123] Marsall M, Engelmann G, Skoda EM, Teufel M, Bäuerle A (2022). Measuring electronic health literacy: development, validation, and test of measurement invariance of a revised German version of the eHealth Literacy Scale. J Med Internet Res.

[124] Marsall M, Dinse H, Schröder J, Skoda EM, Teufel M, Bäuerle A (2024). Assessing electronic health literacy in individuals with the post-COVID-19 condition using the German revised eHealth Literacy Scale: validation study. JMIR Form Res.

[125] Marsall M, Weigl M, Lüttel D, Müller H (2025). Digital health literacy: a cross-sectional survey study among patients after hospitalization in Germany. Z Evid Fortbild Qual Gesundhwes.

[126] Maurud S, Lunde L, Moen A, Opheim R (2025). Mapping conditional health literacy and digital health literacy in patients with inflammatory bowel disease to optimise availability of digital health information: a cross-sectional study. Scand J Gastroenterol.

[127] Moon Z, Zuchowski M, Moss-Morris R, Hunter MS, Norton S, Hughes LD (2022). Disparities in access to mobile devices and e-health literacy among breast cancer survivors. Support Care Cancer.

[128] Muellmann S, Wiersing R, Zeeb H, Brand T (2025). Digital health literacy in adults with low reading and writing skills living in Germany: mixed methods study. JMIR Hum Factors.

[129] Neves AL, Smalley KR, Freise L, Harrison P, Darzi A, Mayer EK (2021). Determinants of use of the care information exchange portal: cross-sectional study. J Med Internet Res.

[130] Nurtsch A, Teufel M, Jahre LM (2024). Drivers and barriers of patients’ acceptance of video consultation in cancer care. Digit Health.

[131] Oliveira L, Zandonadi RP, Nakano EY (2024). From validation to assessment of e-health literacy: a study among higher education students in Portugal. Healthcare (Basel).

[132] Olsbø S, Kiserud SG, Hermansen Å, Hamilton Larsen M, Bjørnland K (2024). Health literacy in parents of children with Hirschsprung disease: a novel study. Pediatr Surg Int.

[133] Olsbø S, Larsen MH, Kiserud SG, Hagen TS, Hermansen Å, Bjørnland K (2025). Parental health literacy in anorectal malformation: needs and challenges. Pediatr Surg Int.

[134] Olszewski R, Watros KM, Brzeziński J (2025). COVID-19 health communication strategies for older adults: chatbots and traditional media. Adv Clin Exp Med.

[135] Pacut A, Duplaga M, Więcek S (2025). The relationship between stress, anxiety, and health literacy in parents of children with chronic gastroenterological diseases: a multi-center cross-sectional study. BMC Public Health.

[136] Palisi A (2024). Digitale Gesundheitskompetenz bei chronischen, nicht-spezifischen Rückenschmerzen [Article in German]. MSK-Muskuloskel Phys.

[137] Pan CC, De Santis KK, Muellmann S (2025). Sociodemographics and digital health literacy in using wearables for health promotion and disease prevention: cross-sectional nationwide survey in Germany. J Prev (2022).

[138] Papp-Zipernovszky O, Horváth MD, Schulz PJ, Csabai M (2021). Generation gaps in digital health literacy and their impact on health information seeking behavior and health empowerment in Hungary. Front Public Health.

[139] Piper L, De Cosmo LM, Benvenuto M, Viola C (2024). How do the determinants of collaborative consumption influence its use in healthcare? A managerial perspective. Int J Health Policy Manag.

[140] Pisl V, Volavka J, Chvojkova E, Cechova K, Kavalirova G, Vevera J (2021). Dissociation, cognitive reflection and health literacy have a modest effect on belief in conspiracy theories about COVID-19. Int J Environ Res Public Health.

[141] Pisl V, Volavka J, Chvojkova E, Cechova K, Kavalirova G, Vevera J (2021). Willingness to vaccinate against COVID-19: the role of health locus of control and conspiracy theories. Front Psychol.

[142] Poot CC, Meijer E, Fokkema M, Chavannes NH, Osborne RH, Kayser L (2023). Translation, cultural adaptation and validity assessment of the Dutch version of the eHealth Literacy Questionnaire: a mixed-method approach. BMC Public Health.

[143] Qiu CS, Lunova T, Greenfield G (2025). Determinants of digital health literacy: International cross-sectional study. J Med Internet Res.

[144] Ramjee S, Mohamedthani H, Patel AU (2023). The effect of remote digital services on health care inequalities among people under long-term dermatology follow-up: cross-sectional questionnaire study. JMIR Dermatol.

[145] Ramstad KJ, Brørs G, Pettersen TR (2023). eHealth technology use and eHealth literacy after percutaneous coronary intervention. Eur J Cardiovasc Nurs.

[146] Rognsvåg T, Nordmo IK, Bergvad IB (2024). Digital health literacy in Norwegian patients with hip and knee arthroplasty: normative data from a cross-sectional study. Acta Orthop.

[147] Rokohl AC, Pine NS, Adler W (2023). Health literacy in patients wearing prosthetic eyes: a prospective cross-sectional study. Curr Eye Res.

[148] Rosenmeier N, Busk D, Dichman C, Nielsen KM, Kayser L, Wagner MK (2025). Technology readiness level and self-reported health in recipients of an implantable cardioverter defibrillator: cross-sectional study. JMIR Cardio.

[149] Scacchi A, Lo Moro G, Giacomini G (2024). Trust levels toward health care and government: insights from TrustMe, an Italian cross-sectional study. J Prev Med Hyg.

[150] Schaeffer D, Klinger J, Berens EM (2021). Health literacy in Germany before and during the COVID-19 pandemic. Gesundheitswesen.

[151] Schaeffer D, Gille S, Berens EM (2023). Digitale Gesundheitskompetenz der Bevölkerung in Deutschland: Ergebnisse des HLS-GER 2 [Article in German]. Gesundheitswesen.

[152] Schaeffer D, Klinger J, Berens EM (2024). Digitale Gesundheitskompetenz von Personen mit und ohne Migrationserfahrung – Ein Vergleich von zwei Querschnittbefragungen [Article in German]. Präv Gesundheitsf.

[153] Schmieding ML, Kopka M, Bolanaki M (2025). Impact of a symptom checker app on patient-physician interaction among self-referred walk-in patients in the emergency department: multicenter, parallel-group, randomized, controlled trial. J Med Internet Res.

[154] Schomakers EM, Lidynia C, Vervier LS, Calero Valdez A, Ziefle M (2022). Applying an extended UTAUT2 model to explain user acceptance of lifestyle and therapy mobile health apps: survey study. JMIR Mhealth Uhealth.

[155] Schulz PJ, Pessina A, Hartung U, Petrocchi S (2021). Effects of objective and subjective health literacy on patients’ accurate judgment of health information and decision-making ability: survey study. J Med Internet Res.

[156] Sippel A, Riemann-Lorenz K, Pöttgen J (2022). Validation of the German eHealth impact questionnaire for online health information users affected by multiple sclerosis. BMC Med Inform Decis Mak.

[157] Sjöström A, Hajdarevic S, Hörnsten Å, Öberg U, Isaksson U (2021). Experiences of online COVID-19 information acquisition among persons with type 2 diabetes and varying eHealth literacy. Int J Environ Res Public Health.

[158] Sjöström AE, Hajdarevic S, Hörnsten Å, Kristjánsdóttir Ó, Castor C, Isaksson U (2023). The Swedish version of the eHealth Literacy Questionnaire: translation, cultural adaptation, and validation study. J Med Internet Res.

[159] Sjöström A, Hajdarevic S, Hörnsten Å, Isaksson U (2024). eHealth literacy and health-related internet use among Swedish primary health care visitors: cross-sectional questionnaire study. JMIR Form Res.

[160] Smoła P, Zwierczyk U, Duplaga M (2024). Transactional e-health literacy and its association with e-health services use in Polish adults: a cross-sectional study. Front Digit Health.

[161] Sollie M, Hansen M, Thomsen JB (2023). Health technology readiness amongst patients with suspected breast cancer using the READHY-tool: a cross-sectional study. J Med Syst.

[162] Spanakis P, Lorimer B, Newbronner E (2023). Digital health literacy and digital engagement for people with severe mental ill health across the course of the COVID-19 pandemic in England. BMC Med Inform Decis Mak.

[163] Spindler H, Dyrvig AK, Schacksen CS (2022). Increased motivation for and use of digital services in heart failure patients participating in a telerehabilitation program: a randomized controlled trial. Mhealth.

[164] Springer F, Hambsch PK, Mehnert-Theuerkauf A, Nicolay NH (2025). Digital support and artificial intelligence in cancer patients undergoing radiation therapy: patient utilization, acceptance and attitudes. Front Oncol.

[165] Stajszczyk M, Świerkowska G, Smolik K, Domysławska I, Charkiewicz K, Samborski W (2023). The perspective of Polish patients with rheumatoid arthritis: treatment expectations, patient-reported outcomes, and digital literacy (the SENSE study). Reumatologia.

[166] Stephan J, Gehrmann J, Dehner JC, Stullich A, Richter M (2025). Development and validation of the eHealth Literacy and Use Scale (eHLUS) to measure medical app literacy. Public Health.

[167] Stephen DA, Nordin A, Johansson UB, Nilsson J (2025). eHealth literacy and its association with demographic factors, disease-specific factors, and well-being among adults with type 1 diabetes: cross-sectional survey study. JMIR Diabetes.

[168] Sylwander C, Wahl AK, Andersson MLE, Haglund E, Larsson I (2023). Health literacy in individuals with knee pain: a mixed methods study. BMC Public Health.

[169] Terp R, Kayser L, Lindhardt T (2021). Older patients' competence, preferences, and attitudes toward digital technology use: explorative study. JMIR Hum Factors.

[170] Thorsen IK, Rossen S, Glümer C, Midtgaard J, Ried-Larsen M, Kayser L (2020). Health technology readiness profiles among Danish individuals with type 2 diabetes: cross-sectional study. J Med Internet Res.

[171] Totaro M, Cicolini G, Bianconi A (2026). Digital health literacy in patients with hypertension: a cross-sectional study. J Clin Nurs.

[172] Tschamper MK, Wahl AK, Hermansen Å, Jakobsen R, Larsen MH (2022). Parents of children with epilepsy: characteristics associated with high and low levels of health literacy. Epilepsy Behav.

[173] Turnbull J, Prichard J, MacLellan J, Pope C (2024). eHealth literacy and the use of NHS 111 online urgent care service in England: cross-sectional survey. J Med Internet Res.

[174] Ullrich G, Bäuerle A, Vogt H (2025). Digital health literacy and attitudes toward eHealth technologies among patients with cardiovascular disease and their implications for secondary prevention: survey study. JMIR Form Res.

[175] Vahteristo A, Jylhä V, Kuusisto H (2025). The use and readiness for eHealth and eWelfare among young adults. Health Informatics J.

[176] Valan L, Isaksson U, Hörnsten A, Carlsund A (2025). Evaluating the impact of digital support on parental stress in Swedish child health care: results from an intervention study. Int J Pediatr.

[177] Valentim P, Arriaga M, Nogueira P, Costa A (2025). Digital and navigational health literacy in surgical patients: vulnerabilities in the transition to post-discharge care. Healthcare (Basel).

[178] Van Rhoon L, McSharry J, Byrne M (2022). Development and testing of a digital health acceptability model to explain the intention to use a digital diabetes prevention programme. Br J Health Psychol.

[179] Villadsen SF, Hadi H, Ismail I, Osborne RH, Ekstrøm CT, Kayser L (2020). eHealth literacy and health literacy among immigrants and their descendants compared with women of Danish origin: a cross-sectional study using a multidimensional approach among pregnant women. BMJ Open.

[180] Vitolo M, Ziveri V, Gozzi G (2022). DIGItal health literacy after COVID-19 outbreak among frail and non-frail cardiology patients: the DIGI-COVID study. J Pers Med.

[181] Wångdahl J, Jaensson M, Dahlberg K, Nilsson U (2020). The Swedish version of the Electronic Health Literacy Scale: prospective psychometric evaluation study including thresholds levels. JMIR Mhealth Uhealth.

[182] Wångdahl J, Dahlberg K, Jaensson M, Nilsson U (2021). Arabic version of the Electronic Health Literacy Scale in Arabic-speaking individuals in Sweden: prospective psychometric evaluation study. J Med Internet Res.

[183] Wecker H, Höllerl L, Schick TS, Biedermann T, Zink A, Ziehfreund S (2024). Patient journey and disease-related digital media usage: a cross-sectional study among dermatology patients across Germany. J Dtsch Dermatol Ges.

[184] Wetzel AJ, Klemmt M, Müller R, Rieger MA, Joos S, Koch R (2024). Only the anxious ones? Identifying characteristics of symptom checker app users: a cross-sectional survey. BMC Med Inform Decis Mak.

[185] Zrubka Z, Brito Fernandes Ó, Baji P (2020). Exploring eHealth literacy and patient-reported experiences with outpatient care in the Hungarian general adult population: cross-sectional study. J Med Internet Res.

[186] Zrubka Z, Vékás P, Németh P (2022). Validation of the PAM-13 instrument in the Hungarian general population 40 years old and above. Eur J Health Econ.

[187] Zwierczyk U, Sowada C, Duplaga M (2022). Eating choices-the roles of motivation and health literacy: a cross-sectional study. Nutrients.

[188] Zwierczyk U, Kobryn M, Duplaga M (2023). The awareness of the role of commercial determinants of health and the readiness to accept restrictions on unhealthy food advertising in Polish society. Nutrients.

[189] The HLS19 Consortium of the WHO Action Network M-POHL (2022). The HLS19-DIGI Instrument to Measure Digital Health Literacy.

[190] Kayser L, Rossen S, Karnoe A (2019). Development of the multidimensional Readiness and Enablement Index for Health Technology (READHY) tool to measure individuals’ health technology readiness: initial testing in a cancer rehabilitation setting. J Med Internet Res.

[191] Osborne RH, Elsworth GR, Whitfield K (2007). The Health Education Impact Questionnaire (heiQ): an outcomes and evaluation measure for patient education and self-management interventions for people with chronic conditions. Patient Educ Couns.

[192] Osborne RH, Batterham RW, Elsworth GR, Hawkins M, Buchbinder R (2013). The grounded psychometric development and initial validation of the Health Literacy Questionnaire (HLQ). BMC Public Health.

[193] van der Vaart R, Drossaert CHC, de Heus M, Taal E, van de Laar M (2013). Measuring actual eHealth literacy among patients with rheumatic diseases: a qualitative analysis of problems encountered using Health 1.0 and Health 2.0 applications. J Med Internet Res.

[194] Soellner R, Huber S, Reder M (2014). The concept of eHealth literacy and its measurement: German translation of the eHEALS. J Media Psychol.

[195] Efthymiou A, Middleton N, Charalambous A, Papastavrou E (2019). Adapting the eHealth literacy scale for carers of people with chronic diseases (eHeals-Carer) in a sample of Greek and Cypriot carers of people with dementia: reliability and validation study. J Med Internet Res.

[196] Paige SR, Stellefson M, Krieger JL, Miller MD, Cheong J, Anderson-Lewis C (2019). Transactional eHealth literacy: developing and testing a multi-dimensional instrument. J Health Commun.

[197] Paige SR, Stellefson M, Krieger JL, Anderson-Lewis C, Cheong J, Stopka C (2018). Proposing a transactional model of eHealth literacy: concept analysis. J Med Internet Res.

[198] Paulhus DL, Vazire S (2007). Handbook of Research Methods in Personality Psychology.

[199] Podsakoff PM, MacKenzie SB, Lee JY, Podsakoff NP (2003). Common method biases in behavioral research: a critical review of the literature and recommended remedies. J Appl Psychol.

[200] Bethlehem J (2010). Selection bias in web surveys. Int Statistical Rev.

[201] (2025). Digital economy and society statistics - households and individuals. Eurostat.

[202] Medero K, Merrill K, Ross MQ (2022). access across the digital divide for intersectional groups seeking web-based health information: national survey. J Med Internet Res.

[203] Cheng C, Beauchamp A, Elsworth GR, Osborne RH (2020). Applying the electronic health literacy lens: systematic review of electronic health interventions targeted at socially disadvantaged groups. J Med Internet Res.

[204] Vannieuwenhuyze J, Loosveldt G, Molenberghs G (2010). A method for evaluating mode effects in mixed-mode surveys. Public Opin Q.

[205] Tran AD, White AE, Torok MR, Jervis RH, Albanese BA, Walter EJS (2024). Lessons learned from a sequential mixed-mode survey design to recruit and collect data from case-control study participants: formative evaluation. JMIR Form Res.

[206] Koch MJ, Wienrich C, Straka S, Latoschik ME, Carolus A (2024). Overview and confirmatory and exploratory factor analysis of AI literacy scale. Comput Educ Artif Intell.

[207] Lintner T (2024). A systematic review of AI literacy scales. NPJ Sci Learn.

[208] Wang X, Zhang C, Qi Y (2025). Digital health literacy questionnaire for older adults: instrument development and validation study. J Med Internet Res.

[209] Quinn K (2010). Methodological considerations in surveys of older adults: technology matters. Int J Emerg Technol Soc.

[210] Kebede AS, Ozolins LL, Holst H, Galvin K (2022). Digital engagement of older adults: scoping review. J Med Internet Res.

