# Supplementary material for: Measuring eHealth Literacy in the European Economic Area, Switzerland, and the United Kingdom: Scoping Review
Source: J Med Internet Res. 2026 May 22;28:e87461. doi: 10.2196/87461 (PMC13200168; doi:10.2196/87461)
Supplement: Multimedia Appendix 1 [file jmir-v28-e87461-s001.docx]

**Multimedia Appendix 1: Inclusion and Exclusion Criteria**

| **Category** | **Inclusion criteria** | **Exclusion criteria** |
| --- | --- | --- |
| **Study design** | - Empirical study | - Review |
| **Publication form** | - Paper published in a peer-reviewed scientific journal | - Study protocol - Conference paper - Gray literature - Book chapters |
| **Publication date** | - 2020 or later |  |
| **Availability** | - Abstract and full text |  |
| **Language** | - English - German |  |
| **Geographic focus** | - European Economic Area - Switzerland - United Kingdom | - Cross-country studies without clear focus on the target regions |
| **Measurement instrument** | - All items or subscales of an original, translated or adapted eHL measurement instrument intended for calculating eHL scores | - Measurement instrument   not capturing the skills described in Norman and Skinner’s [10, p. 1] 2006 definition of eHL (ie, “the ability to seek, find, understand, and appraise health information from electronic sources and apply the knowledge gained to addressing or solving a health problem”)   - Measurement instrument solely for subdimensions of eHL (eg, traditional literacy, health literacy, information literacy, scientific literacy, media literacy, and computer literacy [10]) - Context-specific eHL measurement instrument (eg, COVID-19, health conditions) |
| **Age group** | - Focus on adults | - Focus on children or adolescents |
| **Perspective** |  | - Exclusively individuals with a professional perspective (eg, health professionals, IT specialists, teachers, trainers, as well as students in health, IT, education or communication study programs) |
