# Supplementary material for: Measuring eHealth Literacy in the European Economic Area, Switzerland, and the United Kingdom: Scoping Review
Source: J Med Internet Res. 2026 May 22;28:e87461. doi: 10.2196/87461 (PMC13200168; doi:10.2196/87461)
Supplement: Multimedia Appendix 2 [file jmir-v28-e87461-s002.docx]

**Multimedia Appendix 2: Search strings, Filters and Expanders.^a^**

| **Information source** | **Language** | **Search string** | **Filter** | **Expander** |
| --- | --- | --- | --- | --- |
| CINAHL | English | (TI (“assess*” OR “measur*” OR “framework” OR “questionnaire” OR “survey” OR “scale” OR “screen*” OR “tool” OR “instrument” OR “test” OR “evaluat*” OR “psychometric” OR “performance”) OR AB (“assess*” OR “measur*” OR “framework” OR “questionnaire” OR “survey” OR “scale” OR “screen*” OR “tool” OR “instrument” OR “test” OR “evaluat*” OR “psychometric” OR “performance”)) AND (TI (“digital health” OR “e-health” OR “eHealth” OR “electronic health” OR “digital medicine” OR “mHealth” OR “mobile health” OR “health information” OR “telehealth”) OR AB (“digital health” OR “e-health” OR “eHealth” OR “electronic health” OR “digital medicine” OR “mHealth” OR “mobile health” OR “health information” OR “telehealth”) OR MH (“digital health” OR “telemedicine” OR “telehealth”)) AND (TI (“literacy” OR “competenc*” OR “skill*” OR “capabilit*”) OR AB (“literacy” OR “competenc*” OR “skill*” OR “capabilit*”) OR MH (“health literacy”)) | Abstract available; English, German; 2020/01/01–2025/12/31 [initial search]; 01/2025–Today (01/2026) [updated search] | Apply equivalent subjects |
| CINAHL | German | (TI (“Bewert*” OR “Mess*” OR “Erfass*” OR “Framework” OR “Fragebogen” OR “Umfrage” OR “Skala” OR “Messinstrument” OR “Test” OR “psychometrisch*” OR “Leistung*”) OR AB (“Bewert*” OR “Mess*” OR “Erfass*” OR “Framework” OR “Fragebogen” OR “Umfrage” OR “Skala” OR “Messinstrument” OR “Test” OR “psychometrisch*” OR “Leistung*”)) AND (TI (“digital*”) OR AB (“digital*”) OR MH (“digital health” OR “telemedicine” OR “Telehealth”)) AND (TI (“Gesundheitskompetenz”) OR AB (“Gesundheitskompetenz”) OR MH (“health literacy”)) | Abstract available; Englisch, German; 2020/01/01–2025/12/31 [initial search]; 01/2025–Today (01/2026 [updated search] | Apply equivalent subjects |
| PubMed | English | ((“assess*”[tiab] OR “measur*”[tiab] OR “framework”[tiab] OR “questionnaire”[tiab] OR “survey”[tiab] OR “scale”[tiab] OR “screen*”[tiab] OR “tool”[tiab] OR “instrument”[tiab] OR “test”[tiab] OR “evaluat*”[tiab] OR “psychometric”[tiab] OR “performance”[tiab]) AND (“digital health”[tiab] OR “e-health”[tiab] OR “eHealth”[tiab] OR “electronic health”[tiab] OR “digital medicine”[tiab] OR “mHealth”[tiab] OR “mobile health”[tiab] OR “health information”[tiab] OR “telehealth”[tiab] OR “digital health”[MeSH Terms] OR “telemedicine”[MeSH Terms]) AND (“literacy”[tiab] OR “competenc*”[tiab] OR “skill*”[tiab] OR “capabilit*”[tiab] OR “health literacy”[MeSH Terms])) | Abstract; English, German; 2020/1/1–2025/12/31 [initial search]; 2025/01/31–2026/12/31 [updated search] | – |
| PubMed | German | ((“Bewert*”[tiab] OR “Mess*”[tiab] OR “Erfass*”[tiab] OR “Framework”[tiab] OR “Fragebogen”[tiab] OR “Umfrage”[tiab] OR “Skala”[tiab] OR “Messinstrument”[tiab] OR “Test”[tiab] OR “psychometrisch*”[tiab] OR “Leistung*”[tiab]) AND (“digital*”[tiab] OR “Digital Health”[MeSH Terms] OR “telemedicine”[MeSH Terms]) AND (“Gesundheitskompetenz”[tiab] OR “Health Literacy”[MeSH Terms])) | Abstract; English, German; 2020/1/1–2025/12/31 [initial search]; 2025/01/31–2026/12/31 [updated search] | – |
| Google Scholar | English | “assessment” OR “measurement” OR “framework” OR “questionnaire” OR “survey” OR “scale” OR “screening” OR “tool” OR “instrument” OR “test” OR “evaluation” OR “psychometric” OR “performance” AND “digital health” OR “eHealth” AND “literacy” OR “competence” | 2020–2025 [initial search]; 2025–2026 [updated search] | – |
| Google Scholar | German | “Bewertung” OR “Messung” OR “Erfassung” OR “Framework” OR “Fragebogen” OR “Umfrage” OR “Skala” OR “Messinstrument” OR “Test” OR “psychometrische” OR “psychometrisch” OR “Leistung” AND “digital” OR “digitale” AND “Gesundheitskompetenz” | 2020–2025 [initial search]; 2025–2026 [updated search] | – |

^a^Searches were conducted on January 31, 2025 (initial searches) and January 28, 2026 (updated searches). The search strings were developed based on the Population/Concept/Context (PCC) framework [38]. As the aim was to identify empirical papers that described studies involving individuals in general (population), no subgroup-specific terms were included. Instead, the search string contains terms and available MeSH terms in CINAHL and PubMed related to measurement instruments (concept) and eHL (context).
