# Supplementary material for: Measuring eHealth Literacy in the European Economic Area, Switzerland, and the United Kingdom: Scoping Review
Source: J Med Internet Res. 2026 May 22;28:e87461. doi: 10.2196/87461 (PMC13200168; doi:10.2196/87461)
Supplement: Multimedia Appendix 3 [file jmir-v28-e87461-s003.docx]

## **Multimedia Appendix 3: Meta Data of the Included Papers**

| **Author, publication year** | **Title** | **Full text language** | **Main objective** | **Objective to validate an eHealth literacy instrument^a^** | **Data collection period** | **eHealth literacy measurement frequency per participant** | **Target age or, if not available, qualitative age description, actual age range, or age groups** | **Specific participant’s perspective** | **Country focus** | **Data collection language** | **Sample size reported for central eHL assessment(s) or, if unavailable, total sample** | **eHealth literacy instrument^b^** | **Data collection method, mode** | **Limitations regarding eHL instrument, eHL-related data collection method and mode, as reported in the limitation section or, if not available, the discussion section** |
| --- | --- | --- | --- | --- | --- | --- | --- | --- | --- | --- | --- | --- | --- | --- |
| Ahmed et al., 2025 | Assessing the User Experience of the EU Mobile App for Cancer Prevention: Mixed Methods Study | English | “[T]o identify enablers, barriers, and user requirements for the use and maintenance of the English version of the EU Mobile App for Cancer Prevention, focusing on how usability varied across individuals with different levels of digital health literacy and diverse sociodemographic backgrounds. In addition, user feedback on mock wireframes—visual representations of the app’s interface and functionality—was gathered to evaluate usability and ease of use, providing insights for tailoring the app design to a broader population” | – | 2023–2024 | 1 | ≥18 | Individuals with an internet-enabled smartphone or tablet | Cyprus, Finland, Germany, Hungary, Portugal, Slovenia, Spain | English or the local language: Greek, Finnish, German, Hungarian, Portuguese, Slovenian, Spanish | n=76 (7–13 participants per country) | eHEALS^c^ | Survey, – | Self-report bias:   - Self-reported eHL, no objective assessment |
| Ahmed et al., 2025 | Piloting a cancer awareness app across six European countries: a pre-post study | English | “[E]valuated the usability of the EU Mobile App for Cancer Prevention and changes in cancer awareness associated with use across six European countries (Cyprus, Germany, Hungary, Portugal, Slovenia, Spain), focusing on variations by DHL and sociodemographic factors” | – | 2024 | 1 | ≥18 | Individuals with access to an internet-enabled smartphone | Cyprus, Germany, Hungary, Portugal, Slovenia, Spain, other EU/EEA countries [not explicitly reported] | English or the local language: German, Hungarian, Portuguese, Slovenian, Spanish, Greek | n=77 [post-usage survey participants for whom eHL scores were reported and analyzed, 3–19 participants per country, 2 from other EU/EEA countries] | eHEALS | Survey, online | Self-report bias:   - Self-reported eHL, “potential biases” |
| Almeida et al., 2024 | Evaluating E-Health Literacy, Knowledge, Attitude, and Health Online Information in Portuguese University Students: A Cross-Sectional Study | English | “[T]o assess e-health literacy in a sample of Portuguese university students and its association with the level of knowledge and seeking for COVID-19-related information” | – | 2022–2023 | 1 | – | University students | Portugal | Portuguese | n=534 | eHEALS | Survey, online | – |
| Andersen et al., 2024 | Profiles of health literacy and digital health literacy in clusters of hospitalised patients: a single-centre, cross-sectional study | English | “To investigate health literacy (HL) and digital health literacy (eHL) among patients hospitalised in surgical and medical wards using a cluster analysis approach” | – | 2021 | 1 | ≥18 | Hospitalized patients | Norway | Norwegian | n=254 [participants for whom eHL scores were calculated] | eHLQ^d^ | Survey, paper-based | – |
| Bäuerle et al., 2023 | Psychometric properties of the German revised version of the eHealth literacy scale in individuals with cardiac diseases: Validation and test of measurement invariance | English | “[E]valuation of the psychometric properties of the German revised version of the eHealth literacy scale (GR-eHEALS) in individuals with coronary artery disease (CAD) and congestive heart failure (CHF)” | ✓ | 2021–2022 | 1 | >18 | Individuals with a diagnosis of a cardiac disease (at least CAD or CHF) and internet access | Germany | German | n=315 [participants who completed all eHL items] | eHEALS | Survey, online | Self-report bias:   - “[S]elf-assessment” of eHL; no comparison with “actual behaviors or use of behavior-based measurement methods”   Selection bias:   - Potential overrepresentation of “individuals who are comfortable using the internet and digital devices” and under-representation of individuals “who are less familiar with the web” due to online data collection |
| Bendtsen et al., 2024 | Evaluation of an mHealth App on Self-Management of Osteoporosis: Prospective Survey Study | English | “[T]o investigate the eHealth literacy of Danish patients with osteoporosis, as well as the usability and acceptability of the app “My Bones”” | – | 2020–2023 | 1 | >45 | Postmenopausal patients with diagnosed osteoporosis, access to, and the ability to use a digital device | Denmark | Danish | n=90 [completed the eHL section of the Q0 questionnaire] | eHLQ | Survey, online, paper-based | – |
| Bergh et al., 2025 | Assessment of Technology Readiness in Norwegian Older Adults With Long-Term Health Conditions Receiving Home Care Services: Cross-Sectional Questionnaire Study | English | “[T]o understand the technology readiness level of a group of older adults who were provided home care services in order to address the present and future needs of this group in relation to the implementation of digital health care services” | – | 2021–2023 | 1 | ≥65 | Individuals living at home and receiving home care services with long-term health conditions | Norway | Norwegian | n=147–149 [depending on the eHL sub-scales] | READHY^e^ | Survey, face-to-face, paper-based, [inferred; “either self-reported by themselves or in an interview with the health care staff. The participants were approached in a face-to-face setting where they were informed about the study, signed the consent form, and completed the questionnaires”] | – |
| Bergman et al., 2021 | Health literacy and e-health literacy among Arabic-speaking migrants in Sweden: a cross-sectional study | English | “[T]o explore comprehensive health literacy (CHL) and electronic health literacy (eHL) among Arabic-speaking migrants in Sweden” | – | 2019 | 1 | ≥18 | – | Sweden | Arabic, Swedish | n=622 [participants with eHL scores] | eHEALS | Survey, paper-based [“[p]articipants were asked to complete the questionnaire at the study site. Completed questionnaires were then collected by the researcher or the key stakeholder”] | – |
| Bergman et al., 2023 | Validity and reliability of the arabic version of the HLS-EU-Q16 and HLS-EU-Q6 questionnaires | English | “[T]o psychometrically examine the Arabic versions of HLS-EU-Q16 and HLS-EU-Q6 and their response patterns among Arabic-speaking persons in Sweden” | – | 2019 | 1 [eHL assessment results not reported for retest analysis] | ≥18 | – | Sweden | Arabic | n=289 [participants with eHL scores] | eHEALS | Survey, paper-based | Selection bias:   - Inclusion of individuals with functional literacy, exclusion of individuals with “limited functional literacy” due to self-administered paper-based data collection |
| Bergman et al., 2023 | Validity and reliability of the swedish versions of the HLS-EU-Q16 and HLS-EU-Q6 questionnaires | English | “[T]o evaluate the psychometrics of the Swedish versions of the HLS-EU-Q16 and HLS-EU-Q6, instruments that aims to assess health literacy” | – | 2019 | 1 [eHL results not reported for retest analysis ] | ≥18 | – | Sweden | Swedish | n=314 [participants with eHL scores] | eHEALS | Survey, paper-based [inferred from the context, eg, “participants marked their questionnaires with a code comprising the first three letters of their mother’s name and the year she was born”) | Selection bias:   - Inclusion of individuals with functional literacy due to use of self-administered questionnaires |
| Bevilacqua et al., 2021 | eHealth Literacy: From Theory to Clinical Application for Digital Health Improvement. Results from the ACCESS Training Experience | English | “[R]eports the results of an Italian innovative eHealth training for the European project ACCESS [...] The ACCESS eHealth training has the ambitious purpose of advancing toward a clearer definition and measurement of eHealth literacy in practical scenarios, improving the overall eHealth literacy and thus supporting the positive use of technology” | – | 2020 | 2 [baseline + follow-up] | ≥50 [inferred from the mention of "older adults” and the age groups: 50–>75] | Individuals with access to the internet and digital devices | Italy | Italian | n=58 | eHEALS | Survey, – | – |
| Bíró et al., 2023 | Investigation of the relationship of general and digital health literacy with various health-related outcomes | English | “[T]o examine the impacts of general and digital health literacy on health behaviour, confidence in vaccination, self-perceived health, and health care utilization” | – | 2020 | 1 | 18–64 [sample was restricted to this age group; in the survey, older adults participated as well] | – | Hungary | Hungarian | n=830 [participants included in analyses] | HLS_19_-DIGI^f^ | Survey, telephone-based [computer-assisted telephone interviews] | – |
| Breil et al., 2022 | Comparing the Acceptance of Mobile Hypertension Apps for Disease Management Among Patients Versus Clinical Use Among Physicians: Cross-sectional Survey | English | “[T]o investigate the determinants of the acceptance of health apps (in terms of intention to use) among patients for personal use and physicians for clinical use in German-speaking countries. Moreover, we assessed patients’ preferences regarding different delivery modes for self-care service (face-to-face services, apps, etc)” | – | 2019 | 1 | ≥18 | Individuals who were a patient with self-reported hypertension or a practicing physician | Germany | German | n=209 [patients; n=163; physicians: n=46] | eHEALS | Survey, online | – |
| Brørs et al., 2020 | Psychometric Properties of the Norwegian Version of the Electronic Health Literacy Scale (eHEALS) Among Patients After Percutaneous Coronary Intervention: Cross-Sectional Validation Study | English | “[T]o translate and adapt the eHealth Literacy Scale (eHEALS) to conditions in Norway, and to determine its psychometric properties” | ✓ | 2017–2018 | 2 [test-retest] | ≥18 | Patients undergoing percutaneous coronary intervention, living at home, with access to electronic equipment and the internet | Norway | Norwegian | n=1,659 [participants with eHL scores] | eHEALS | Survey, paper-based | – |
| Burzyńska et al., 2022 | Evaluating the Psychometric Properties of the eHealth Literacy Scale (eHEALS) among Polish Social Media Users | English | “[T]o evaluate the psychometric aspects of the Polish version of this instrument (eHEALS-Pl) among social media users” | ✓ | 2019 | 1 | Not limited [“the age of the respondents was not limited”, actual age range: 14–72 years] | Individuals with a verified account on at least one social platform | Poland | Polish | n=1,527 | eHEALS | Survey, online [computer-assisted web interviews] | Self-report bias   - Unspecified “response bias” due to online data collection |
| Chaniaud et al., 2022 | Translation and Validation Study of the French Version of the eHealth Literacy Scale: Web-Based Survey on a Student Population | English | “[T]o translate eHEALS to French and validate the French version of eHEALS (F-eHEALS)” | ✓ | 2019–2020 | 2 [test-retest] | <35 included in the analysis, actual age range: 16–33 | University students | France | French | n=328 | eHEALS | Survey, online | Self-report bias:   - Potential self-assessment bias due to risk of eHL over- or underestimation   eHEALS:   - Insufficient consideration of skills related to “new forms of interaction” (“Health 2.0”); need “to integrate new items related to these new forms of interaction into eHEALS […] to measure the variability of eHealth literacy encompassing competencies from Health 2.0” |
| Chatsatrian et al., 2025 | Usability Evaluation of Digital Health Applications for Older People With Depressive Disorders: Prospective Observational Study in a Mixed Methods Design | English | “[A]ssessed the usability of DiGA deprexis and Selfapy for adults aged ≥60 years with mild to moderate depression” | – | 2024 | 1 | ≥60 | Individuals with a diagnosis of mild or moderate depressive disorder owning a digital device that can be used to run digital health applications | Germany | German | n=18 | eHEALS | Survey, – | – |
| Dale et al., 2020 | Testing Measurement Properties of the Norwegian Version of Electronic Health Literacy Scale (eHEALS) in a Group of Day Surgery Patients | English | “[T]o examine the measurement properties of the Norwegian version of the eHEALS, as it was used in a group of patients undergoing day surgery” | ✓ | 2019 | 1 | ≥18 | Patients scheduled for day surgery | Norway | Norwegian | n=109 | eHEALS | Survey, paper-based | Self-report bias:   - “[U]ncertainty about the respondent’s intended meaning” due to potential comprehension issues   eHEALS:   - Not clear “whether inadequate eHealth literacy is a result of insufficient health literacy, digital literacy, or a combination hereof” - “[W]eak correlation between the eHEALS and people’s internet use” due to missing differentiation “between those with high and low internet skills”; need for a “broader perspective on health literacy” and an instrument that covers “operational, formal, informational skills as well as strategic internet skills” - Insufficient explanation regarding the theoretical anchoring of eHEALS in the Lily model |
| De Santis et al., 2021 | Digitization and Health in Germany: Cross-sectional Nationwide Survey | English | “[T]o investigate the attitudes toward and the use of digital technologies for health-related purposes using a nationwide survey” | – | 2020 | 1 | ≥14 | Individuals using the internet | Germany | German | n=928 [participants who completed all eHL items] | eHEALS | Survey, telephone-based [computer-assisted telephone interviews] | Self-report bias:   - Unspecified potential self-report bias |
| De Santis et al., 2024 | Digitisation and health: Second nationwide survey of internet users in Germany | English | “[T]o investigate in more detail the internet use in health context and digital technology use for health promotion and disease prevention in Germany” | – | 2022 | 1 | ≥18 | Individuals using the internet | Germany | German | n=932 [participants who completed all eHL items] | eHEALS | Survey, telephone-based [computer-assisted telephone interviews] | Self-report bias:   - Potential over- or underestimation of eHL “due to recall and social desirability bias” - Potential “general biases associated with any surveys (e.g. social desirability bias)” |
| Duplaga, 2020 | The Acceptance of Key Public Health Interventions by the Polish Population Is Related to Health Literacy, But Not eHealth Literacy | English | “[T]o assess the determinants of the attitudes of Polish society regarding the ST [sugar tax] and to vaccinations” | – | 2016 | 1 | “[A]dult sample” | – [eHL scores calculated only for internet users] | Poland | Polish | n=849 [internet users for whom eHL scores were calculated] | eHEALS | Survey, telephone-based [computer-assisted telephone interviews] | “[L]ess profound consideration” due to telephone-based data collection:   - “[L]ess profound consideration of the issues presented in the questionnaire” by the participants due to computer assisted telephone-based data collection |
| Duplaga, 2020 | The Determinants of Conspiracy Beliefs Related to the COVID-19 Pandemic in a Nationally Representative Sample of Internet Users | English | “[T]o assess the prevalence of conspiracy beliefs related to the COVID-19 pandemic in a representative sample of Poland’s Internet users. In addition, the relationships of supporting attitudes towards conspiracy theories and sociodemographic variables, HL and eHL were analysed” | – | 2020 | 1 | ≥18 | Individuals using the internet | Poland | Polish | n=1,002 | eHEALS | Survey, online [computer-assisted web-based interviews] | Selection bias:   - Exclusion of “non-users of the Internet” due to online data collection, especially older individuals, overrepresentation of “younger users” |
| Duplaga, 2020 | The Use of Fitness Influencers’ Websites by Young Adult Women: A Cross-Sectional Study | English | “[T]o make an assessment of the determinants of regular access to fitness influencers’ sites (FIS) and their relationship with the health behaviors of young adult women” | – | 2018 | 1 | Actual age range: 18–35 | Women | Poland | Polish | n=1,030 | eHEALS | Survey, online [computer-assisted web-based interviews] | – |
| Duplaga, 2022 | A Nationwide Natural Experiment of e-Health Implementation during the COVID-19 Pandemic in Poland: User Satisfaction and the Ease-of-Use of Remote Physician’s Visits | English | “[T]o assess the use of e-health services in the initial phase of the COVID-19 pandemic. Furthermore, the factors related to user satisfaction and positive assessment of the ease-of-use of RPVs were analyzed” | – | 2020 | 1 | “[A]dult” | Individuals using the internet | Poland | Polish | n=2,410 | eHEALS | Survey, online [computer-assisted web-based interviews] | Selection bias:   - Inclusion of “Internet users”, exclusion of “Internet non-users” due to online data collection |
| Duplaga, 2022 | The Roles of Health and e-Health Literacy, Conspiracy Beliefs and Political Sympathy in the Adherence to Preventive Measures Recommended during the Pandemic | English | “[T]o assess the roles of health (HL) and e-health literacy (eHL), conspiracy beliefs, political sympathy, and religious practices in the adherence to COVID-19 preventive measures after adjusting for sociodemographic factors” | – | 2020 | 1 | “[A]dult” | Individuals using the internet | Poland | Polish | n=2,410 | eHEALS | Survey, online [computer-assisted web-based interviews] | Selection bias:   - Inclusion of “Internet users”, “finings apply only to the population of adult Internet users. Therefore, they cannot be extrapolated to the whole adult population in Poland, especially to older adults and the elderly” |
| Duplaga and Grysztar, 2021 | The Association between Future Anxiety, Health Literacy and the Perception of the COVID-19 Pandemic: A Cross-Sectional Study | English | ”[T]o establish the level and the determinants of future anxiety in Polish society related to the COVID-19 pandemic three months after the introduction of the state of epidemic” | – | 2020 | 1 | “[A]dults”, actual age range: 18–74 | Individuals using the internet | Poland | Polish | n=1,002 | eHEALS | Survey, online [computer-assisted web-based interviews] | – |
| Duplaga and Turosz, 2022 | User satisfaction and the readiness-to-use e-health applications in the future in Polish society in the early phase of the COVID-19 pandemic: A cross-sectional study | English | “[T]o assess the determinants of user satisfaction and the readiness-to-use e-health applications in Polish society” | – | 2020 | 1 | “[A]dult” | Individuals using the inhttps://pubmed.ncbi.nlm.nih.gov/33956368/ternet | Poland | Polish | n=1,002 | eHEALS | Survey, online [computer-assisted web-based interviews] | – |
| Efthymiou et al., 2021 | Health literacy and eHealth literacy and their association with other caring concepts among carers of people with dementia: A descriptive correlational study | English | “[T]o identify the levels of HL and eHL among carers of PwD [people with dementia] in Greece and Cyprus and to search for the associations with other caring concepts” | – | 2017–2019 | 1 | >18 | Primary caregivers of PwD and secondary caregivers | Greece and Cyprus | Greek | n=202 | eHEALS-Carer^g^ | Survey, paper-based | – |
| Efthymiou et al., 2025 | Validation of the eHealth literacy scales: comparison between the shorter and longer versions | English | “[T]o validate and compare the two scales in Greek: the eHeals and the revised eHeals-Extended” | ✓ | 2022 | 1 | >18 | – | Greece | Greek | n=401 | eHEALS, eHEALS-E^h^ | Survey, online | eHEALS:   - “[L]acks items assessing basic aspects of Web 2.0 and 3.0”   eHEALS-E:   - “[T]wo factors (Awareness of sources and Recognizing quality and meaning) are similarly not well discriminated” - Need “to include more items related to Web 3.0, given the rapid pace of technological advancements” - Availability in “Slovenian and Greek” only |
| Fugmann et al., 2025 | Electronic Health Literacy, Psychological Distress, and Quality of Life in Urological Cancer Patients: A Longitudinal Study During Transition from Inpatient to Outpatient Care | English | “[E]xamined associations between eHL, psychological symptoms, and quality of life during transition from inpatient to outpatient care” | – | 2020–2021 | 1 | ≥18 | Hospitalized urological cancer patients with an expected survival time of >6 months, and a score of ≥5 on the Distress Thermometer and/or desire for support | Germany | German | n=108 | eHEALS | Survey, paper-based | eHEALS:   - Lacking reflection of “online communities, social media, and AI-based health applications”/” Web 2.0 and 3.0 competencies” |
| Garcia et al., 2025 | eHealth literacy in a migrant community and its association with chronic disease | English | “[T]o assess digital health literacy levels in a migrant population and to examine their relationship with sociodemographic characteristics and health-related variables” | – | 2022 | 1 | ≥18 | Migrants and their direct descendants | Portugal | Portuguese | n=101 | eHLQ | Survey, face-to-face, paper-based | – |
| García-García et al., 2022 | Factors Influencing eHealth Literacy among Spanish Primary Healthcare Users: Cross-Sectional Study | English | “[T]o assess the eHealth literacy of a population using primary care services in Madrid (Spain) and to investigate the association between this literacy and sociodemographic variables” | – | – | 1 | >18 | Patients using primary care nursing services | Spain | Spanish | n=166 | eHLQ | Survey, paper-based, face-to-face [if assistance was required] | – |
| García-García et al., 2023 | Correlation between Health and eHealth Literacy and a Healthy Lifestyle: A Cross-Sectional Study of Spanish Primary Healthcare Patients | English | “[T]o analyze the correlation between health and eHealth literacy and a healthy lifestyle in primary care patients of a healthcare center in Madrid (Spain), as well as to examine which sociodemographic and health and eHealth variables influence a healthy lifestyle” | – | – | 1 | >18 | Patients using primary care nursing services | Spain | Spanish | n=166 | eHLQ | Survey, paper-based, face-to-face [if assistance was required] | – |
| Gehrmann et al., 2025 | Pilot study of an app-supported psychosocial prevention intervention: a mixed-methods approach | English | “To pilot the new prevention intervention “RV Fit Mental Health” which combines an intensive inpatient phase with an app-supported digital outpatient phase” | – | 2023–2024 | 1 | Actual age range: 34–64 | Insured individuals with rejected medical psychosomatic rehabilitations applications and a diagnosis according to the International Classification of Diseases diagnoses (affective disorders, phobic disorders, any anxiety disorders, adoption disorder, somatoform disorders, burnout who took part in the inpatient phase of the RV Fit Mental Health | Germany | German | n=21 | eHEALS, eHLUS^i^ | Survey, online | – |
| Geiger et al., 2024 | Association between eHealth literacy and health outcomes in German athletes using the GR-eHEALS questionnaire: a validation and outcome study | English | “[F]irstly, to test the factorial structure of the GR-eHEALS in German athletes and assess its construct validity by examining both convergent and discriminant validity; and secondly, to explore the associations between eHealth literacy and health-related outcomes (i.e. substance use and injuries)” | ✓ | 2021–2022 | 1 | >18; 18–90 included in the analysis | Athletes with internet access | Germany | German | n=282 [participants with complete data on all eHL variables] | eHEALS | Survey, online | Self-report bias:   - Potential bias due to self-assessment of eHL; “may not provide an accurate representation of skills and competences”; lack of comparison of “self-assessment with actual behaviour”   Selection bias:   - Potential overrepresentation “of individuals who are comfortable using the internet and digital devices” and underrepresentation of individuals “who are less familiar with the internet” due to online data collection |
| Georgsson et al., 2025 | Validation of the eHealth Literacy Scale Instrument in a Restless Legs Syndrome Population: Classical Test Theory and Rasch Analysis Study | English | “[T]o investigate the psychometric properties of eHEALS in patients with RLS [restless legs syndrome] to determine its adequacy and potential utility” | ✓ | – [“[C]ollected during the COVID-19 pandemic”] | 1 | ≥18 | Individuals diagnosed and treated for RLS | Sweden | Swedish | n=754 [385 participants with low eHL, 369 with high eHL] | eHEALS | Survey, paper-based [“To participate, eligible members had to return […] the completed survey in a prestamped envelope”] | – |
| Ghazi et al., 2023 | The prevalence of eHealth literacy and its relationship with perceived health status and psychological distress during Covid-19: a cross-sectional study of older adults in Blekinge, Sweden | English | “[T]o analyze the prevalence of eHealth literacy in older adults living in Blekinge, Sweden, and assess the association of eHealth literacy, psychological distress and perceived health status. Our secondary aim is to measure eHealth literacy’s interactive effect (combined effect) and perceived health status on psychological distress” | – | 2021 | 1 | ≥65 | Participants who were part of the Swedish National Study on Aging and Care in Blekinge except those with severe cognitive disorder and those too frail to answer the questions [eHL assessment included internet users] | Sweden | Swedish | n=364 [participants with eHL scores] | eHEALS | Survey, – | – |
| Giunti et al., 2025 | Feasibility and usability evaluation of a gamified fatigue management mobile application for persons with multiple sclerosis in everyday life | English | “More Stamina, a gamified mHealth application, was developed to support MS [multiple sclerosis] patients by tracking energy expenditure and facilitating fatigue management” | – | 2023 | 1 | >18 | Individuals with a confirmed MS diagnosis for at least 1 year, and an Expanded Disability Status Scale score of <6.5 | Finland | Finnish | n=20 | eHEALS | Survey, paper-based [inferred from the mention that “[p]aper questionnaires were digitized”] | – |
| Hermansen et al., 2023 | Preliminary validity testing of the eHealth Literacy Questionnaire (eHLQ): a Confirmatory Factor Analysis (CFA) in Norwegian hospitalized patients | English | “To perform the first psychometric analysis of the Norwegian version of the eHLQ using confirmative factor analysis procedures in a population of patients admitted to hospital using a cross-sectional design” | ✓ | 2021 | 1 | ≥18 | Hospitalized Patients | Norway | Norwegian | n=260 | eHLQ | Survey, paper-based | – |
| Hernández Encuentra et al., 2024 | Spanish and Catalan Versions of the eHealth Literacy Questionnaire: Translation, Cross-Cultural Adaptation, and Validation Study | English | “[T]o adapt the eHLQ and gather evidence of its psychometric quality in 2 of Spain’s official languages: Spanish and Catalan” | ✓ | 2021–2022 | 1 | ≥18 | – | Spain | Catalan, Spanish | n=800 | eHLQ; eHEALS | Survey, online/electronic [computer-aided web interviews or–with optional support provided by recruiters–on a tablet] | Selection bias:   - Inclusion of individuals with a “basic level of digital skills” and potential exclusion of individuals “with no internet access or no basic skills” due to online data collection   Self-report bias:   - Unspecified “voluntary reporting and self-reporting biases” |
| Hernández Encuentra et al. 2025 | Digital health literacy among the Spanish population: a descriptive and latent class analysis study | English | “To identify different profiles of people by analysing their digital health literacy, with the ultimate goal of providing healthcare organizations with indications to improve the relationship between people and the healthcare system” | – | 2021–2022 | 1 | ≥18 | – | Spain | Spanish | n=396 [participants with eHL scores] | eHLQ, eHEALS | Survey, online, face-to-face | Selection bias:   - Inclusion of individuals with “basic digital skills” due to online data collection; “sample was balanced with 100 face-to-face interviews” |
| Holderried et al., 2021 | Attitude and potential benefits of modern information and communication technology use and telemedicine in cross‑sectoral solid organ transplant care | English | “[Q]uantitatively evaluate use and potential of modern information and communication technology (ICT) in solid organ transplant (SOT) recipients” | – | – | 1 | ≥18 | Patients from the post-transplant follow-up care after kidney, liver, pancreas, or combined organ transplantation | Germany | German [inferred from the country focus] | n=234 | eHEALS | Survey, paper-based [inferred from the mention that “the questionnaire was sent to patients by mail”] | – |
| Holderried et al., 2024 | Unleashing the potential of eHealth in outpatient cancer care for patients undergoing immunotherapy–a quantitative study considering patients’ needs and current healthcare challenges | English | “[E]xplores cancer patients’ perspectives on eHealth and demonstrates how eHealth applications, from the patients’ point of view, can contribute to further improving outpatient immunotherapy” | – | 2019–2021, paused March 2020–August 2020 due to Covid-19 pandemic | 1 | ≥18 | Individuals receiving outpatient therapy with immune checkpoint inhibitors (ICI) or outpatient therapy with ICI in combination with chemotherapy due to cancer and were in good cognitive condition | Germany | German [inferred from the country focus] | n=164 | eHEALS | Survey, paper-based | – |
| Hölgyesi et al., 2024 | The Impact of Parental Electronic Health Literacy on Disease Management and Outcomes in Pediatric Type 1 Diabetes Mellitus: Cross-Sectional Clinical Study | English | “[T]o assess the electronic health literacy of parents caring for children with T1DM [Type 1 Diabetes Mellitus] and investigate its associations with disease management and children’s outcomes” | – | 2021–2022 | 1 | ≥18 | Parents or caregivers of children aged 8-14 years with T1DM diagnosis for at least 3 month who attended routine diabetology care | Hungary | Hungarian | n=150 | eHEALS | Survey, online | – |
| Hölgyesi et al., 2024 | Robot-assisted surgery and artificial intelligence-based tumour diagnostics: social preferences with a representative cross-sectional survey | English | “[T]o assess social preferences for two different advanced digital health technologies and investigate the contextual dependency of the preferences” | – | 2021 | 1 | ≥40 | – | Hungary | Hungarian | n=1,400 | eHEALS | Survey, online | – |
| Holmen et al., 2025 | A Digital Outpatient Service With a Mobile App for Tailored Care and Health Literacy in Adults With Long-Term Health Service Needs: Multicenter Nonrandomized Controlled Trial | English | “[T]o evaluate whether digital outpatient care for 6 months improved health literacy, health-related quality of life (HRQoL), digital/eHealth literacy, and the use of health care services compared with usual care” | – | 2021–2023 | 3 (baseline + 2 follow-ups) [“longitudinal design with 3 assessment points”] | ≥18 | Patients receiving outpatient care in pain, lung, neurology or cancer departments and living at home | Norway | Norwegian | n=162 [baseline] | eHLQ | Survey, online | – |
| Juvalta et al., 2020 | Electronic Health Literacy in Swiss-German Parents: Cross-Sectional Study of eHealth Literacy Scale Unidimensionality | English | “[T]o determine the factor structure of the German eHEALS measure in a sample of parents by adopting classic and modern psychometric approaches. In particular, this study sought to identify the eHEALS validity as a unidimensional index as well as the viability for potential subscales” | ✓ | 2018 | 1 | – | Parents of children aged 1– 24 months | Switzerland | German | n=703 | eHEALS | Survey, online, paper-based | Self-report bias:   - Potential bias with regard to the assessed eHL if parents responded to “eHEALS items from the perspective of child health” rather than their own |
| Katsaliaki, 2024 | Factors influencing use of eHealth services during and after the COVID-19 pandemic | English | “[T]o explore the usage, perceptions, and knowledge of eHealth interventions during and after the COVID-19 pandemic” | – | 2021, 2023 | 1 [data collection at 2 time points (during and after the COVID-19 pandemic] | “[A]dult population”, age groups: 18–>50 years | – | Greece | Greek | n=638 (t1: n=277, t2: n=361) | eHEALS | Survey, online [inferred from the mention that both surveys “were promoted through social media”] | – |
| Knitza et al., 2020 | Mobile Health Usage, Preferences, Barriers, and eHealth Literacy in Rheumatology: Patient Survey Study | English | “[T]o explore mHealth usage, preferences, barriers, and eHealth literacy reported by German patients with rheumatic diseases” | – | 2018–2019 | 1 | ≥18 | Patients with rheumatoid arthritis, psoriatic arthritis, or spondylarthritis | Germany | German | n=193 | eHEALS | Survey, paper-based | Self-report bias:   - Unspecified potential self-report bias [no concrete examples with regard to eHL assessment] |
| Kobryn and Duplaga, 2024 | Does Health Literacy Protect Against Cyberchondria: A Cross-Sectional Study? | English | “[T]o examine the prevalence of cyberchondria among adult internet users in Poland. Furthermore, the study was focused on analyzing the determinants of cyberchondria, with special regard to health literacy (HL) and e-health literacy (eHL)” | – | 2022 | 1 | “[A]dult” | Individuals using the internet | Poland | Polish | n=1,613 | eHEALS | Survey, online [computer-assisted web-based interviews] | eHEALS:   - Potential better reflection of “the skills related to retrieving online information than its critical appraisal” provided by eHEALS |
| Kokwaro et al., 2025 | Digital health literacy among people with bipolar disorder in Germany – a cross-sectional survey | English | “[E]xamining overall patterns of DHL [digital health literacy] among individuals with BD [bipolar disorder] and employing SIMHL [Sørensen’s Integrated Model of Health Literacy] to investigate DHL the role of associated structural, situational/clinical, and technical access factors” | – | 2023–2024 | 1 | ≥18 | Individuals with a self-reported BD diagnosis | Germany | German | n=212 | HLS_19_-DIGI | Survey, online | Self-report bias:   - Self-reported eHL: “subjective and may not fully reflect functional literacy in real-world contexts” |
| König et al., 2024 | Digital Health Literacy of the Population in Germany and Its Association With Physical Health, Mental Health, Life Satisfaction, and Health Behaviors: Nationally Representative Survey Study | English | “[T]o representatively assess the digital health literacy of the population in Germany and relevant subgroups” | – | 2022 | 1 | Age groups: 16–≥65 | Individuals with internet access | Germany | German | n=1,996 [participants who answered all eHL items] | eHEALS | Survey, online | Selection bias:   - Inclusion of individuals with “basic technical skills and access to the internet” and risk of under-representation of “older people” due to online data collection   Self-report bias:   - Potential self-report bias [no concrete examples with regard to eHL measuring] |
| Kostagiolas et al., 2021 | Investigation of the Information-Seeking Behavior of Hospitalized Patients at the General Hospital of Corfu | English | “[T]o investigate the information-seeking behavior of hospitalized patients in a peripheral hospital in Greece, based on Wilson’s theoretical model” | – | 2018 | 1 | >18 | Hospitalized patients | Greece | Greek [inferred from the country focus] | n=144–147 [depending on the eHL items] | eHEALS | Survey, – | – |
| Kretzschmar et al., 2025 | mHealth Use, Preferences, Barriers, and eHealth Literacy Among Patients With Inflammatory Bowel Disease: Survey Study | English | “[T]o investigate the use of mHealth as well as the preferences, obstacles, and eHealth literacy reported by patients with IBD [inflammatory bowel disease] in Germany” | – | 2023 | 1 | ≥18 | Patients with a confirmed diagnosis of Crohn or ulcerative colitis | Germany | German | n=200 | eHEALS | Survey, paper-based | Self-report bias:   - Self-reported instruments, potential “social desirability bias”; potential overestimation of “digital competencies” |
| Kristjánsdóttir et al., 2022 | eHealth literacy and socioeconomic and demographic characteristics of parents of children needing paediatric surgery in Sweden | English | “[T]o describe different eHealth literacy domains among parents of children needing paediatric surgery in Sweden, and the correlation between these eHealth literacy domains and parents' socioeconomic factors and demographic characteristics” | – | 2020 | 1 | ≥18 | Legal guardians (parents) with a child below 4 years of age undergoing pediatric surgery | Sweden | Swedish | n=30 [completed the questionnaires] | eHLQ | Survey, online | Self-report bias:   - “[A]ssessment were self-reported”, no “objective measures” used |
| Kubb and Foran, 2022 | Online Health Information Seeking for Self and Child: An Experimental Study of Parental Symptom Search | English | “[T]o examine the features of web-based health-related search behaviors based on video-coded observational data, to investigate which psychological and relational factors are related to successful symptom search appraisal, and to examine the differences in search-related outcomes among self-seekers and by-proxy seekers” | – | – | 1 | ≥18 | Parents of a child aged between 0 and 6 years using the internet at least sometimes for health-related information seeking who did not have children with chronic illnesses | Austria | German | n=46 | eHEALS | Survey, paper-based | – |
| Lambrecht et al., 2021 | Quality of a Supporting Mobile App for Rheumatic Patients: Patient-Based Assessment Using the User Version of the Mobile Application Scale (uMARS) | English | “(1) to assess the quality of the self-management app Rheuma Auszeit using the validated uMARS (User Version of the Mobile App Rating Scale) app quality assessment tool and (2) to evaluate the association between uMARS scores and patients’ characteristics” | – | 2018–2019 | 1 | Age groups: 18–≥60 years | Patients with rheumatoid arthritis, psoriatic arthritis and spondyloarthritis | Germany | German | n=126 | eHEALS | Survey, paper-based | Self-report bias:   - “[O]nly patient-reported items were collected in the study” |
| Lear et al., 2022 | Patients’ Willingness and Ability to Identify and Respond to Errors in Their Personal Health Records: Mixed Methods Analysis of Cross-sectional Survey Data | English | “[T]o evaluate patients’ willingness and ability to identify and respond to errors in their PHRs [personal health records]” | – | 2018–2019 | 1 | ≥18 | Patients who had used personal health records | United Kingdom | English [inferred from the country focus] | n=445 | eHEALS | Survey, online | Selection bias:   - “Web-based survey”; inclusion of “digitally empowered patients” |
| Lear et al., 2022 | Perceptions of Quality of Care Among Users of a Web-Based Patient Portal: Cross-sectional Survey Analysis | English | “[T]o describe perceived changes in the quality of care among users of a web-based patient portal and to identify the characteristics of patients who perceive the greatest benefit of portal use” | – | 2018–2019 | 1 | ≥18 | Patients who had used personal health records | United Kingdom | English [inferred from the country focus] | n=445 | eHEALS | Survey, online | Selection bias:   - “[I]nclusion of patients who are digitally literate and more able to fully engage with patient portals” due to online data collection |
| Levin-Zamir et al., 2025 | Measuring digital health literacy and its associations with determinants and health outcomes in 13 countries | English | “[T]o validate the digital health literacy measure HLS_19_-DIGI, applied in the European Health Literacy Survey (2019–2021) of the WHO M-POHL network” | ✓ | 2019–2021 [depending on the country] | 1 [independent data collection in each country] | ≥18 | – | Austria, Belgium, Czechia, Denmark, France, Germany, Hungary, Ireland, Israel, Norway, Portugal, Slovakia, Switzerland | Austria, Germany: German, Belgium: Dutch, French, Czechia: Czech, Denmark: Danish, France: French, Hungary: Hungarian, Ireland: English, Israel: Hebrew, Arabic, Russian, Norway: Norwegian, Portugal: Portuguese, Slovakia: Slovak, Switzerland: French, German, Italian | n=28,057 (1,000–4,487 per country) | HLS_19_-DIGI | Survey, Austria: telephone-based, Belgium: online, Czechia: telephone-based, online, Denmark: online, France: online, Germany: face-to-face, Hungary: telephone-based, Ireland: telephone-based, Israel: telephone-based, online, Norway: telephone-based, Portugal: telephone-based, Slovakia: face-to-face, Switzerland: online | Selection bias:   - Potential overrepresentation of individuals with “high DHL” in countries, in which data collection was carried out online   Limited comparability of the results:   - “Differences in sampling and data collection limit the extent of country comparisons” |
| Linnestad et al., 2025 | Exploring digital health literacy clusters in a Norwegian stroke survivor population–A cross-sectional study (NORFAST) | English | “[T]o identify DHL-profiles [digital health literacy] among stroke survivors and possible associations with sociodemographic and clinical characteristics” | – | 2023–2024 | 1 | 18–80 | Patients registered in the Norwegian Stroke Registry who had experienced a first-ever stroke with a pre-stroke modified Rankin Scale score of 0-2 | Norway | Norwegian | n=177 | eHLQ | Survey, electronic, telephone-based [“ depending on the patient's preference“] | – |
| Lo Moro et al., 2022 | Exploring the Relationship between COVID-19 Vaccine Refusal and Belief in Fake News and Conspiracy Theories: A Nationwide Cross-Sectional Study in Italy | English | “[T]o assess COVID-19 VR [vaccine refusal] in Italy and its relationship with belief in FNs [fake news]/CTs [conspiracy theories]. Secondarily, it explored the conviction in FNs and CTs and associated variables” | – | 2021 | 1 | ≥18 | – | Italy | Italian | n=1,292 [n=755 with adequate and n=537 with inadequate eHL] | eHEALS | Survey, online [computer-assisted web interviews] | – |
| Lortz et al., 2025 | Patient acceptance of video consultations in cardiology | English | “[I]nvestigate[..] patient acceptance of video consultations in cardiovascular care using a survey-based approach, assessing key factors influencing their integration into routine practice” | – | 2024 | 1 | ≥18 | Individuals with internet access receiving outpatient treatment for cardiac and related diseases | Germany | German | n=337 | eHEALS | Survey, online | Selection bias:   - Potential underrepresentation of “individuals with limited internet access or lower digital literacy” due to online data collection   eHEALS:   - “[T]here are newer instrument”; need "to validate newer and more comprehensive instruments” |
| Lowe et al., 2022 | Usability Testing of a Digital Assessment Routing Tool for Musculoskeletal Disorders: Iterative, Convergent Mixed Methods Study | English | “[T]o assess and resolve all serious DART [Digital Assessment Routing Tool] usability issues to create a positive user experience and enhance system adoption before conducting randomized controlled trials for the integration of DART into musculoskeletal management pathways” | – | – | 1 | >18 | Individuals with experience of a musculoskeletal condition who accessed the internet at least once every three months and had access to a smartphone, tablet, or laptop | United Kingdom | English | n=20 [included in eHL-related analysis] | eHEALS | Survey, online | – |
| Luz et al., 2025 | Psychometric Analysis of the eHealth Literacy Scale in Portuguese Older Adults (eHEALS-PT24): Instrument Development and Validation | English | “[T]o describe the translation, adaptation, and validation process of the eHealth Literacy Scale (eHEALS) in Portuguese older adults” | ✓ | 2022, 2023 | 1 [independent data collection in study 1 and study 2: 1] | ≥65 | Patients from Health Family Units | Portugal | Portuguese | n=381 [study 1: n=80,  study 2: n=301] | eHEALS | Survey, face-to-face [“in-person questionnaires“] | eHEALS:   - “[M]easures a narrow scope of eHealth literacy” - Potentially not adequately reflecting “complex concept of eHealth literacy nowadays since it was developed before the social media era (Web 2.0)” |
| Marsall et al., 2022 | Measuring Electronic Health Literacy: Development, Validation, and Test of Measurement Invariance of a Revised German Version of the eHealth Literacy Scale | English | “[D]evelopment and validation of a revised German eHealth literacy scale. In particular, this study aimed to focus on high methodological and psychometric standards to provide a valid and reliable instrument for measuring eHealth literacy in the German language” | ✓ | 2020 | 1 | ≥18 | – | Germany | German | n=470 | eHEALS | Survey, online | Self-report bias:   - “[S]elf-assessment” of eHL; no comparison with “actual behaviors”, no ”behavior-based measurement”   Selection bias:   - Potential overrepresentation of individuals “familiar with the internet” and under-representation of individuals “who rarely use the internet” due to online data collection |
| Marsall et al., 2024 | Assessing Electronic Health Literacy in Individuals With the Post–COVID-19 Condition Using the German Revised eHealth Literacy Scale: Validation Study | English | “[T]o evaluate the validity and reliability of the German Revised eHealth Literacy Scale (GR-eHEALS) in individuals with the post–COVID-19 condition” | ✓ | 2022 | 1 | ≥18 | Individuals with internet access who had a confirmed COVID-19 infection in the past, and reported current post COVID-19 symptoms | Germany | German | n=330 | eHEALS | Survey, online | Selection bias:   - Potential overrepresentation of “individuals with higher affinity to the use of digital media“ due to online data collection   Self-report bias:   - Unspecified potential “response bias” due to use of “self-report instruments” |
| Marsall et al., 2025 | Digital health literacy: A cross-sectional survey study among patients after hospitalization in Germany | English | “[T]o assess eHL in a sample of patients after their hospital stay and to explore potential sociodemographic determinants” | – | 2023 | 1 | ≥18 | Individuals who were hospitalized in the last 24 months | Germany | German | n=1,000 | eHEALS | Survey, online | Self-report bias:   - Potential “uncertainty regarding the accuracy of reported eHL levels” as eHL was self-reported   eHEALS:   - “[C]overs a small part of digital health literacy”; “more up-to-date constructs for assessing eHL have been developed” |
| Maurud et al., 2025 | Mapping conditional health literacy and digital health literacy in patients with inflammatory bowel disease to optimise availability of digital health information: a cross-sectional study | English | “[E]xamines IBD [inflammatory bowel disease] patients’ health literacy and digital health literacy covariance with clinical, demographic and patient-reported outcomes” | – | 2023–2024 | 1 | ≥18 | Individuals with a verified diagnosis of IBD | Norway | Norwegian | n=380 | eHLQ | Survey, online, paper-based | – |
| Moon et al., 2021 | Disparities in access to mobile devices and e‑health literacy among breast cancer survivors | English | “[T]o explore e-health literacy rates and access to smartphones and tablets in a large sample of breast cancer survivors” | – | – | 1 | >18 | Women with primary breast cancer diagnosis and prescribed hormone therapy in the previous three years | United Kingdom | English | n=1,870 [completed the eHL items; n=1,860 included in analysis of eHL scores across different variables] | eHEALS | Survey, online, paper-based | – |
| Muellmann et al., 2025 | Digital Health Literacy in Adults With Low Reading and Writing Skills Living in Germany: Mixed Methods Study | English | “[T]o assess digital health literacy in adults with low reading and writing skills and to explore which digital health tools they use in daily life” | – | 2022–2023 | 1 | 18–64 | Individuals with limited reading and writing skills in German | Germany | German, Russian, Twi, Arabic, English, Turkish | n=96 [thereof, n=2 with “[u]nknown” eHL] | eHEALS | Survey, face-to-face | eHEALS:   - “[D]ifferences between the individual items were hardly recognizable for participants, even if the interview was conducted in their native language” - Lack of “uniformly used cutoff scores reported in the literature” |
| Neves et al., 2021 | Determinants of Use of the Care Information Exchange Portal: Cross-sectional Study | English | “[T]o identify the determinants of the use of the Care Information Exchange [CIE], a shared patient portal program in the United Kingdom” | – | 2018–2019 | 1 | ≥18 | Patients registered with the CIE portal, regardless of their actual use | United Kingdom | English [inferred from the country focus] | n=650 [individuals who had provided at least information regarding gender and age] | eHEALS | Survey, online | Selection bias:   - Exclusion of “less tech-savvy individuals, individuals with less digital literacy, with less consistent access to the internet” due to online data-collection” |
| Nurtsch et al., 2024 | Drivers and barriers of patients’ acceptance of video consultation in cancer care | English | “[T]o evaluate cancer patients’ acceptance and determine drivers and barriers of VC in cancer care as a supplement to personal visits to an oncologist in Germany” | – | 2022–2023 | 1 | Adults [“majority” as inclusion criterion] | Individuals with cancer diagnosis, cancer care (currently or in the past) and internet access | Germany | German | n=350 | eHEALS | Survey, online | Selection bias:   - Inclusion of “people with higher levels of digital confidence and less fear of the internet” and potential exclusion of “individuals who lack internet access or the necessary devices” due to online data collection, especially “elderly people and those with a lower level of education”   Self-report bias:   - Unspecified potential self-report bias |
| Oliveira et al., 2024 | From Validation to Assessment of e-Health Literacy: A Study among Higher Education Students in Portugal | English | “[T]o validate and assess the e-Health Literacy Scale among those in Portuguese higher education. In addition, this study focused on measuring their e-health literacy levels and investigating how these skills relate to different sociodemographic variables” | ✓ | 2023–2024 | 2 [test-retest] | ≥18 | Higher education students | Portugal | Portuguese | n=245 | eHEALS | Survey, online | Self-report bias   - “[S]et of challenges” including “inaccurate” or “socially desirable” answers due to the use of “self-reported data” |
| Olsbø et al., 2024 | Health literacy in parents of children with Hirschsprung disease: a novel study | English | “To explore health literacy (HL) among parents of children with Hirschsprung disease (HD)” | – | 2023–2024 | 1 | – | Parents or primary caregivers of children under 16 who had undergone HD surgery | Norway | Norwegian | n=128 [participants with eHL scores] | eHEALS | Survey, online, paper-based | – |
| Olsbø et al., 2025 | Parental health literacy inanorectal malformation: needs and challenges | English | “Explore health literacy (HL) among parents of children with anorectal malformation (ARM) and identify the predictors of HL” | – | 2023–2024 | 1 | 21–57 | Parents of children aged < 16 years with ARM | Norway | Norwegian | n=127 [participants with eHL scores] | eHEALS | Survey, online, paper-based | – |
| Olszewski et al., 2025 | COVID-19 health communication strategies for older adults: Chatbots and traditional media | English | “To identify areas of interest and preferred sources of information related to the COVID-19 pandemic among older adults and to verify their eHealth competencies” | – | 2022–2022 | 1 | “[Y]ounger adults”, “older adults” | Individuals studying at university, and listeners of University of the Third Age (U3A) | Poland | Polish | n=>399 [responses depending on the eHL items] | eHEALS | Survey, paper-based | Self-report bias:   - Potential “self-report bias, e.g. false or inaccurate answers” due to “self-administrated” data-collection, “although the researchers supervised the completion of the questionnaires and respondents answered questions about the questionnaires” |
| Pacut et al., 2025 | The relationship between stress, anxiety, and health literacy in parents of children with chronic gastroenterological diseases: a multi-center cross-sectional study | English | “[T]o assess the relationship between health literacy and both perceived stress and anxiety for parents of children with chronic gastrointestinal diseases” | – | 2023 | 1 | – | Parents of children being treated for chronic gastrointestinal conditions | Poland | Polish [inferred] | n=562 | eHEALS | Survey, paper-based | Self-report bias:   - Potential “recall or response biases” due to the use of self-report instruments [no concrete example with regard to eHL] |
| Palisi, 2024 | Digital Health Literacy for Chronic Non-Specific Back Pain. Questionnaire Survey | German | “[T]o determine how high the DHL [digital health literacy] of people with chronic non-specific back pain is and whether there is a connection with the specific procedure for online searches” | – | 2022–2023 | 1 | Adults [“Erwachsene”] | Individuals with non-specific pain in the lumbar spine for at least 3 months using the internet to search for health information | Germany | German | n=58 [participants for whom eHL scores were calculated] | HLS_19_-DIGI | Survey, online | – |
| Pan et al., 2024 | Sociodemographics and Digital Health Literacy in Using Wearables for Health Promotion and Disease Prevention: Cross-Sectional Nationwide Survey in Germany | English | “[I]nvestigate[…] differences in sociodemographic factors and digital health literacy between wearable users and non-users, whether the association with wearable use varies across age groups and its potential mediator” | – | 2022 | 1 | ≥18 | Individuals using the internet | Germany | German | n=932 [participants with complete data on eHL items] | eHEALS | Survey, telephone-based [computer-assisted telephone interviews] | Self-report bias:   - Potential “recall bias and subjectivity” due to use of “self-reported data” - Self-assessed eHL “does not constitute a comprehensive evaluation of [...] actual competencies” |
| Papp-Zipernovszky et al., 2021 | Generation Gaps in Digital Health Literacy and Their Impact on Health Information Seeking Behavior and Health Empowerment in Hungary | English | “[T]o explore [...] generational differences as related to self-perceived eHealth literacy and health care system utilization” | – | 2018–2020 | 1 | ≥18, age groups: 18–72 | – | Hungary | Hungarian | n=491 [participants with eHL scores] | eHEALS | Survey, online | – |
| Petrič and Atanasova, 2024 | Validation of the extended e-health literacy scale: structural validity, construct validity and measurement invariance | English | “[T]o validate the Extended e-health literacy scale (eHEALS-E) on general population and investigate its structural validity and internal consistency, construct validity in terms of convergent and discriminant validity, and examine its measurement invariance across gender, age, education and social status” | ✓ | 2020 | 1 | ≥18 | Individuals who at least occasionally used one internet service to gain health-related information | Slovenia | Slovenian | n=1,944 | eHEALS-E | Survey, online, face-to-face [computer-assisted personal interviews], paper-based | eHEALS-E:   - Cognitive interviews not carried out “for the whole set of items” in Slovenian and not for the translated English items - Limited practicality of eHEALS-E in research and practice due to its length; potential existence of “redundant items”; “small distinction between the dimensions Awareness of sources and Recognizing quality and meaning” - High dependency of the “Being smart on the Net dimension” on “advances in technology”, potential need for a “regular review”   Self-report bias/eHEALS-E:   - Potential “social desirability bias” due to some eHEALS-E “items requiring self-assessment of knowledge and skills”; “further testing” required |
| Piper et al., 2024 | How Do the Determinants of Collaborative Consumption Influence Its Use in Healthcare? A Managerial Perspective | English | “[T]o scrutinize the underlying motivations that may prompt those responsible for health to adopt models of collaborative consumption (CC) as business innovation. Furthermore, the study seeks to assess the congruence of determinants influencing the intention to utilize CC in healthcare, comparing perspectives between responsible for health and digital health consumers” | – | 2023, 2024 | 1 | Actual age range: 18–71 | – | Italy | Italian [inferred from the country focus] | n=752 | eHEALS | Survey, online | – |
| Pisl et al., 2021 | Dissociation, Cognitive Reflection and Health Literacy Have a Modest Effect on Belief in Conspiracy Theories about COVID-19 | English | “[I]nvestigate how psychological and cognitive characteristics influence general conspiracy mentality and COVID-related conspiracy theories” | – | 2021 | 1 | – | Individuals studying medicine, pedagogy, and law | Czech Republic | Czech | n=866 | eHEALS | Survey, online | – |
| Pisl et al., 2021 | Willingness to Vaccinate Against COVID-19: The Role of Health Locus of Control and Conspiracy Theories | English | “[I]nvestigate how the readiness to believe conspiracy theories and the three dimensions of health locus of control (HLOC) affect the attitude toward vaccination” | – | 2021 | 1 | – | Individuals studying medicine, pedagogy, and law | Czech Republic | Czech | n=866 | eHEALS | Survey, online | – |
| Poot et al., 2023 | Translation, cultural adaptation and validity assessment of the Dutch version of the eHealth Literacy Questionnaire: a mixed-method approach | English | “[T]o translate and culturally adapt the original eHealth Literacy Questionnaire (eHLQ) to Dutch and to collect initial validity evidence” | ✓ | – | 1 | ≥18 | Individuals with internet access | Netherlands | Dutch | n=1,650 | eHLQ | Survey, online | – |
| Qiu et al., 2025 | Determinants of Digital Health Literacy: International Cross-Sectional Study | English | [T]o assess the levels of digital health literacy in 4 countries (United Kingdom, Sweden, Italy, and Germany) and explore potential associations between digital health literacy and demographic characteristics and self-perceived health status” | – | 2020 | 1 [independent data collection in each country] | ≥18 | – | Germany, Italy, Sweden, United Kingdom | English, German, Italian, Swedish [official language of each country] | n=6,331  [1,015–2,161 per country] | eHEALS | Survey, online | Selection bias:   - Potential overrepresentation of “participants more familiar with online tools” due to online data collection; potential “underrepresentation of participants lacking the ability to access online tools”   eHEALS:   - Potentially inadequately capturing the “full extent of digital health literacy in the modern day, where health technology is advancing at pace and there is widespread use of social media” |
| Ramjee et al., 2023 | The Effect of Remote Digital Services on Health Care Inequalities Among People Under Long-Term Dermatology Follow-Up: Cross-Sectional Questionnaire Study | English | “[T]o inform the redesign of remote services to optimally support the ongoing needs of patients with chronic skin diseases, ensuring that the services are tailored to patients’ digital health literacy requirements” | – | 2021, 2022 | 1 | Age groups: 21–≥80 | Patients with chronic skin conditions requiring long-term dermatology follow-up | United Kingdom | English | n=123 [n=116 included in cluster analysis] | READHY | Survey, face-to-face, telephone-based [with assistance where required] | READHY:   - “[H]ighly comprehensive measure”, potentially led to selection bias; “streamlining the READHY question profile would likely improve its practicality in busy clinical settings” |
| Ramstad et al., 2022 | eHealth technology use and eHealth literacy after percutaneous coronary intervention | English | “[T]o determine the extent to which patients after [percutaneous coronary intervention] PCI are health-related digitally active at baseline, 2 and 6 months after PCI, and to determine the association between patients’ eHealth literacy and their health-related digital activity” | – | 2017–2020 | 1 | ≥18 | Patients  undergoing PCI during index hospitalization, and being community-dwelling | Norway | Norwegian | n=1,970 | eHEALS | Survey, – | – |
| Rognsvåg et al., 2024 | Digital health literacy in Norwegian patients with hip and knee arthroplasty: normative data from a cross-sectional study | English | “[T]o provide eHL [electronic health literacy] norms in a representative group of Norwegian patients, and secondarily to examine the relationships between eHL and health-related quality of life (QoL)” | – | 2022 | 1 | ≥18 | Patients who had received hip and knee arthroplasty 6 to 11 months prior | Norway | Norwegian | n=383 [participants with less than 50% missing values on the eHL instrument included in the analysis] | eHLQ | Survey, paper-based | eHLQ:   - Less “thoroughly tested for psychometric properties as the eHEALS”   Self-report bias:   - Self-reported eHL; potentially different than “actual competence”   Selection bias:   - Potential over- or underrepresentation of “patients with low digital health literacy”, despite paper-based data collection |
| Rokohl et al., 2022 | Health Literacy in Patients Wearing Prosthetic Eyes: A Prospective Cross-Sectional Study | English | “To investigate general and electronic health literacy (HL) levels in prosthetic eye wearing patients, to define factors associated with reduced HL, and to identify a potential healthcare gap” | – | – | 1 | ≥18 | Patients wearing cryolite glass prosthetic eyes | Germany | German | n=148 | eHEALS | Survey, face-to-face | – |
| Rosenmeier et al., 2025 | Technology Readiness Level and Self-Reported Health in Recipients of an Implantable Cardioverter Defibrillator: Cross-Sectional Study | English | “[T]o demonstrate how data from the Readiness for Health Technology Index (READHY), combined with sociodemographic characteristics and exploratory interviews, can be used to construct profiles of recipients of an ICD [implantable cardioverter defibrillators], describing their ability to manage their condition, their need for support, and their digital health literacy. This aims to enhance health care professionals’ understanding of different patient archetypes, serving as guidance in delivering personalized services tailored to the needs, resources, and capabilities of individual recipients of ICDs” | – | 2019–2022 | 1 | “[A]dults” | Recipients of an ICD with primary or secondary prophylactic indications attending a voluntary ICD rehabilitation meeting | Denmark | Danish [inferred] | n=79 | READHY | Survey, – | – |
| Scacchi et al., 2024 | Trust levels toward health care and government: insights from TrustMe, an Italian cross-sectional study | English | “[T]o describe the distrust level in the National Health Service (NHS) and in governmental management of the pandemic, one year after the start of the COVID-19 vaccination campaign” | – | 2022 | 1 | >18 | Individuals visiting healthcare hubs for COVID-19 vaccination | Italy | Italian | n=1,983 [participants with eHL scores] | eHEALS | Survey, paper-based | – |
| Schaeffer et al., 2021 | Health Literacy in Germany before and during the COVID-19 Pandemic | German [abstract also available in English] | “[T]o compare the general and digital health literacy (HL) of the German population before the outbreak of the COVID-19 pandemic and during its persistence and to investigate different changes in population groups” | – | 2019–2020 | 1 [data collection at two points in time (before and during the COVID-19 pandemic); independent samples] | ≥18 | – | Germany | German | n=2,683 (before the pandemic: n=2,151; during the pandemic: n=532) | HLS_19_-DIGI | Survey, face-to-face [paper-based personal interviews] | – |
| Schaeffer et al., 2021 | Digital Health Literacy of the Population in Germany: Results of the HLS-GER 2 | German [abstract also available in English] | “[T]o analyze the extent of DHL [digital health literacy] in the German population, key determinants, and consequences for the use of digital health information resources” | – | 2019–2020 | 1 | ≥18 | – | Germany | German | n=1,996 [participants with eHL score] | HLS_19_-DIGI | Survey, face-to-face [paper-based personal interviews] | – |
| Schaeffer et al., 2024 | Digital health literacy of persons with and without migration experience–a comparison of two cross-sectional surveys | German | “[T]o compare the DHL [digital health literacy] of people without and with personal and parental ME [migration experience]” | – | 2020 | 1 | ≥18 | Individuals without migration experience, individuals with personal or parental migration experience | Germany | German and German, Russian, Turkish | n=1,455 [participants with valid eHL scale values] | HLS_19_-DIGI | Survey, face-to-face [inferred from the mention of “persönlich befragt”] | Self-report bias:   - Self-assessment of eHL |
| Schmieding et al., 2025 | Impact of a Symptom Checker App on Patient-Physician Interaction Among Self-Referred Walk-In Patients in the Emergency Department: Multicenter, Parallel-Group, Randomized, Controlled Trial | English | “[T]o evaluate the effects of an SCA [symptom checker app] on satisfaction with the patient-physician interaction in acute care settings. Additionally [...] examin[ing] its influence on patients’ anxiety and trust in the treating physician” | – | 2022–2023 | 1 | ≥18 | Self-referred patients with a treatment urgency rating of yellow, green, or blue according to the Manchester Triage System | Germany | English, German | n=411 [with eHL scores] | eHEALS | Survey, electronic [“via a tablet computer, with study personnel providing instructions on its use”] | – |
| Schomakers et al., 2022 | Applying an Extended UTAUT2 Model to Explain User Acceptance of Lifestyle and Therapy Mobile Health Apps: Survey Study | English | “[T]o compare the factors influencing the acceptance of lifestyle and therapy apps to better understand what drives and hinders the use of mHealth apps” | – | 2019 | 1 | Actual age range: 16–89 years | Individuals attending a university seminar and their social contacts, both needed access to the internet and digital devices | Germany | German | n=707 | DHLI^j^ | Survey, online | – |
| Schulz et al., 2021 | Effects of Objective and Subjective Health Literacy on Patients’ Accurate Judgment of Health Information and Decision-Making Ability: Survey Study | English | “[T]o determine whether objective and subjective health literacy are independent concepts and to test which of the two was associated more strongly with accurate judgments of the quality of a medical website and with behavioral intentions beneficial to health” | – | – | 1 | 18–65 | Individuals with internet access | Italy | Italian | n=362 | eHEALS | Survey, online | – |
| Sippel et al., 2022 | Validation of the German eHealth impact questionnaire for online health information users affected by multiple sclerosis | English | “[T]o validate the psychometric properties of the German version of the eHealth Impact Questionnaire (eHIQ-G)” | – | 2019–2020 | 1 | ≥18 | Individuals with multiple sclerosis (MS) or with suspected MS and access to the Internet | Germany | German | n=152–158 [varying number of responses across the different domains of the eHL instrument] | eHLQ | Survey, online | Selection bias:   - Overrepresentation of “highly educated” individuals and underrepresentation of “less educated” individuals due to online data collection |
| Sjöström et al., 2021 | Experiences of Online COVID-19 Information Acquisition among Persons with Type 2 Diabetes and Varying eHealth Literacy | English | “[T]o explore online COVID-19 information acquisition experiences among persons with type 2 diabetes and varying eHealth literacy” | – | 2020 | 1 | ≥18 | Individuals diagnosed with type 2 diabetes within the last five years owning a smartphone | Sweden | Swedish | n=58 | eHEALS | Survey, – | Self-report bias:   - Risk of over- or underestimation bias due to self-assessment of eHL; no measurement of “actual demonstrated competencies using the Internet for health-related purposes” |
| Sjöström et al., 2023 | The Swedish Version of the eHealth Literacy Questionnaire: Translation, Cultural Adaptation, and Validation Study | English | “[T]o translate, culturally adapt, and evaluate the psychometric properties of the Swedish version of the eHLQ” | ✓ | 2021 | 1 | ≥18 | Patients visiting primary health care centers and parents of hospitalized children | Sweden | Swedish | n=236 | eHLQ | Survey, paper-based | Sample size:   - Lower sample size due to paper-based data collection instead of paper-based and online data collection |
| Sjöström et al., 2024 | eHealth Literacy and Health-Related Internet Use Among Swedish Primary Health Care Visitors: Cross-Sectional Questionnaire Study | English | “[I]nvestigated eHealth literacy and its association with health-related internet use and sociodemographic characteristics among primary health care visitors” | – | 2020 | 1 | ≥18 | Patients visiting primary health care centers | Sweden | Swedish | n=172 | eHLQ | Survey, paper-based | Self-report bias:   - Potential over- or underestimation bias due to self-assessment of eHL; “people’s perceptions rather than their actual digital competencies”   Sample size:   - Lower sample size due to exclusively paper-based data collection |
| Smoła et al., 2024 | Transactional e-health literacy and its association with e-health services use in Polish adults: a cross-sectional study | English | “[T]o culturally adapt the instrument assessing Transactional e-Health Literacy (TeHL) and examine the association between TeHL and the use of e-health services by Polish adult Internet users” | ✓ | 2023 | 2 [test-retest] | Actual age range: 18–75 | Individuals using the internet | Poland | Polish | n=1,661 | TeHLI^k^, eHEALS | Survey, online [computer-assisted web-based interviews] | – |
| Sollie et al., 2023 | Health Technology Readiness amongst Patients with Suspected Breast Cancer Using the READHY-tool - a Cross-sectional Study | English | “[T]o assess health technology readiness profiles amongst women with a suspected breast cancer diagnosis. Secondly, we wanted to investigate the potential differences between these profiles according to sociodemographic factors and the patients´ current use of technology” | – | 2021 | 1 | 50–69 [inferred from the mention that “[p]atients were referred via the official screening program for breast cancer in Denmark, where all women between 50 - and 69 years are offered a mammogram every two years”] | Female patients with suspected breast cancer | Denmark | Danish | n=92 | READHY | Survey, electronic, paper-based | – |
| Spanakis et al., 2023 | Digital health literacy and digital engagement for people with severe mental ill health across the course of the COVID-19 pandemic in England | English | “[T]o examine how the use of the Internet has changed during the pandemic for people with SMI [severe mental ill health], and explore digital exclusion, symptomatic/health related barriers to internet engagement, and digital health literacy” | – | 2020–2022 | 1 | ≥18 | Individuals with a documented diagnosis of schizophrenia or delusional/ psychotic illness or bipolar disorder | United Kingdom | English [inferred from the country focus] | n=170 [responded to all eHL items] | eHEALS | Survey, online, paper-based, telephone-based | – |
| Spindler et al., 2022 | Increased motivation for and use of digital services in heart failure patients participating in a telerehabilitation program: a randomized controlled trial | English | "[E]valuated changes over time in eHL for heart failure (HF) patients in a telerehabilitation program (the Future Patient Program) compared to a traditional rehabilitation program” | – | – | 2 [after 6 months, after 12 months; not at baseline] | ≥18 | Individuals hospitalized for heart failure within the past two weeks with internet access at home | Denmark | Danish | n=97 | eHLQ | Survey, – | eHLQ:   - “[R]isk of serendipitous results” due to “multidimensional nature” of eHLQ, requiring complex analyses |
| Springer et al., 2025 | Digital support and artificial intelligence in cancer patients undergoing radiation therapy: patient utilization, acceptance and attitudes | English | “[T]o investigate utilization, acceptance and attitudes towards digital support tools and AI [artificial intelligence] within the context of cancer treatment and to identify associated patient-related factors” | – | 2024 | 1 | ≥18 | Patients diagnosed with cancer diagnosis receiving radiotherapy | Germany | German | n=154 | eHEALS | Survey, paper-based | – |
| Stajszczyk et al., 2023 | The perspective of Polish patients with rheumatoid arthritis – treatment expectations, patient-reported outcomes, and digital literacy (the SENSE study) | English | “[T]o determine the satisfaction with treatment and the nature of therapeutic preferences and expectations of Polish patients with moderate to severe RA [rheumatoid arthritis]” | – | 2018–2019 | 1 | ≥18 | Patients with RA who were treated with disease modifying anti-rheumatic drugs at study entry and had suboptimal disease control | Poland | Polish [inferred from the country focus] | n=52 | eHEALS | Survey, – | – |
| Stephan et al., 2025 | Development and validation of the eHealth Literacy and Use Scale (eHLUS) to measure medical app literacy | English | “[T]o develop and validate the eHealth Literacy and Use Scale (eHLUS), a German assessment tool designed to measure health literacy in the context of using medical apps” | ✓ | 2023–2024 | 2 [retest] | 18–67 [reported in the trial registration] | Individuals insured with Deutsche Rentenversicherung Mittelastand [reported in the trial registration] | Germany | German | n=117 | eHLUS; eHEALS | Survey, online | eHLUS:   - Potentially limited “diversity of perspectives due to the expert selection process”; experts participated in the development and validation process - Operational challenges during the expert interviews (eg, “unstable internet connections”) - “[D]eveloped based on the established eHEALS in a context-specific manner”, potentially reducing “the generalizability of the results” |
| Stephen et al., 2025 | eHealth Literacy and Its Association With Demographic Factors, Disease-Specific Factors, and Well-Being Among Adults With Type 1 Diabetes: Cross-Sectional Survey Study | English | “[T]o explore associations between eHealth literacy and demographic factors, disease-specific factors, and well-being among adults with type 1 diabetes” | – | 2022 | 1 | ≥18 | Individuals with type 1 diabetes | Sweden | Swedish | n=301 | eHEALS | Survey, online, paper-based | Selection bias:   - Potential overrepresentation of individuals “with higher eHealth literacy” due to reliance on online data collection - Potential underrepresentation of individuals “who do not use social media”   Self-report bias:   - Assessment of “people’s perceived skills with eHealth; “indirect measure of eHealth literacy”   eHEALS:   - “[S]ingle-factor scale […] developed before the time of social media and mHealth”; inadequately capturing “the current dynamicity, interactivity, and multifaceted nature of the internet, social media, and mobile web”; need to use “newer measures that account for the dynamicity and evolving nature of eHealth literacy” - Found a “ceiling effect in the eHEALS score”; potential “inability to capture true differences between participants achieving the highest possible score”; “may also point toward the outdated content validity […] in the current digital era”; “ceiling effect has not been previously reported in other studies” |
| Sylwander et al., 2023 | Health literacy in individuals with knee pain–a mixed methods study | English | “(1) examined the level of health literacy and associations with lifestyle habits, health status, chronic pain, and radiographic knee osteoarthritis; and (2) explored experiences illuminating health literacy among individuals with knee pain” | – | 2019–2022 | 1 | 30–65 [reported in the trial registration] | Individuals with knee pain | Sweden | Swedish | n=216 [n=175 with sufficient eHL, n=41 with limited eHL] | eHEALS | Survey, – | – |
| Terp et al., 2021 | Older Patients’ Competence, Preferences, and Attitudes Toward Digital Technology Use: Explorative Study | English | “[T]o explore older patients’ readiness (ie, competence, preferences, and attitudes) toward the use of information and communication technology (ICT), and to identify the factors that may act as barriers or facilitators for their engagement with health technology” | – | 2017, 2 participants recruited in 2018 | 1 | ≥65 | Hospitalized patients from internal medicine units | Denmark | Danish | n=25 | READHY | Survey, – | – |
| Thorsen et al., 2020 | Health Technology Readiness Profiles Among Danish Individuals With Type 2 Diabetes: Cross-Sectional Study | English | “[T]o investigate readiness for health technology in relation to mental well-being, sociodemographic, and disease-related characteristics among individuals with T2D [type 2 diabetes]” | – | 2018 | 1 | ≥18 | Individuals with a T2D diagnosis | Denmark | Danish | n=155 | READHY | Survey, face-to-face, paper-based [“questionnaire was administered on-site using paper and pencil and was partly interviewer- and self-administered with the possibility of receiving assistance”] | READHY:   - Potential underrepresentation of vulnerable subgroups due to non-availability of validated versions of READHY in other languages than Danish “at the time of data collection” |
| Totaro et al., 2025 | Digital Health Literacy in Patients With Hypertension: A Cross-Sectional Study | English | “To assess Digital Health Literacy (DHL) levels among hypertensive patients” | – | 2024 | 1 | ≥18 | Patients diagnosed with primary hypertension treated with antihypertension therapy for at least 1 year | Italy | Italian | n=233 [participants with eHL scores] | HLS_19_-DIGI | Survey, paper-based [inferred from the mention that “the participant sealed the completed questionnaire in an envelope and placed it inside a designated collection box”] | Self-report bias:   - Potential “bias due to factors such as social desirability or response bias”; “risk of overestimation or underestimation” |
| Tschamper et al., 2022 | Parents of children with epilepsy: Characteristics associated with high and low levels of health literacy | English | “[T]o investigate characteristics associated with high and low levels of different dimensions of HL [health literacy] in parents” | – | 2020 | 1 | ≥18 | Parents of children aged <12 years diagnosed with epilepsy | Norway | Norwegian | n=252 [participants with eHL scores] | eHEALS | Survey, online, paper-based | Self-report bias   - Potential over- or underestimation bias due to “subjective” assessment of eHL |
| Turnbull et al., 2024 | eHealth Literacy and the Use of NHS 111 Online Urgent Care Service in England: Cross-Sectional Survey | English | “[T]o measure the association between eHealth literacy and the use of NHS (National Health Service) 111 online urgent care service” | – | 2020–2021 | 1 | ≥18 | Individuals who used or did not use NHS 111 online | United Kingdom | English [inferred from the country focus] | n=2,662–2,730 [participants with eHL scores depending on the dimension of the eHL instrument] | eHLQ | Survey, online/electronic [online or on a tablet with assistance by a research nurse, if required] | Self-report bias:   - Self-reported eHL   eHLQ:   - “[C]omplexity” in analyses due to “the requirement to report the 7 dimensions separately”; “single digital literacy score” provided by other instruments   Selection bias:   - “Bias toward digital literacy” and potential underrepresentation of “[s]ome population groups (such as older adults and people with very low educational attainment” due to online data collection |
| Ullrich et al., 2025 | Digital Health Literacy and Attitudes Toward eHealth Technologies Among Patients With Cardiovascular Disease and Their Implications for Secondary Prevention: Survey Study | English | “[T]o analyze eHealth literacy, digital use patterns, and general attitudes toward digital technologies in a collective of patients with cardiovascular disease to identify potential obstacles in implementing mobile health technologies in secondary preventive therapy” | – | 2022 | 1 | ≥18 | Patients with atherosclerotic cardiovascular disease owning a smartphone suitable for apps | Germany | German | n=236 | eHEALS | Survey, – | Self-report bias:   - Self-reported eHL, no comparison with “actual abilities to use the internet to find health information and implement it in daily life”; “actual eHealth literacy can differ” |
| Vahteristo et al., 2025 | The use and readiness for eHealth and eWelfare among young adults | English | “[T]o investigate young adults’ eHealth literacy levels, use, and readiness to use eHealth and eWelfare” | – | 2022 | 1 | 18–29 | Individuals living in one wellbeing services county | Finland | Finnish | n=110 | READHY | Survey, online | – |
| Valan et al., 2025 | Evaluating the Impact of Digital Support on Parental Stress in Swedish Child Health Care: Results From an Intervention Study | English | “[E]valuating a digital support intervention involving parents, child health nurses, and researchers” | – | 2022–2023 | 3 (baseline + 2 follow-ups) | – | Parents of children aged 0–5 years listed at child health care centers | Sweden | Swedish | n=124 | eHEALS | Survey, – | – |
| Valentim et al., 2025 | Digital and Navigational Health Literacy in Surgical Patients: Vulnerabilities in the Transition to Post-Discharge Care | English | “[T]o assess levels of digital and navigational health literacy and their associations in a sample of surgical patients” | – | 2025 | 1 | ≥18 | Individuals admitted for elective or emergency surgery | Portugal | Portuguese | n=82 | HLS_19_-DIGI | Survey, face-to-face | – |
| Van Rhoon et al., 2021 | Development and testing of a digital health acceptability model to explain the intention to use a digital diabetes prevention programme | English | “[T]o develop and test a digital health acceptability model of the factors influencing the intention of adults living in Ireland to use a digital DPP [diabetes prevention program]” | – | 2020–2021 | 1 | ≥18 | Individuals without previous diagnosis of type 1 or type 2 diabetes | Ireland | English | n=316 | eHEALS | Survey, online, paper-based | – |
| Villadsen et al., 2020 | ehealth literacy and health literacy among immigrants and their descendants compared with women of Danish origin: a cross-sectional study using a multidimensional approach among pregnant women | English | “To explore ehealth literacy, ability to actively engage with healthcare providers and health system navigation among pregnant immigrant women and their descendants compared with women of Danish origin” | – | 2016 | 1 | – | Pregnant women attending antenatal care | Denmark | Danish, English | n=405 | eHLQ | Survey, – | – |
| Vitolo et al., 2022 | DIGItal Health Literacy after COVID-19 Outbreak among Frail and Non-Frail Cardiology Patients: The DIGI-COVID Study | English | “[T]o explore the association between frailty, the use of digital tools, and patients’ digital health literacy” | – | 2022 | 1 | ≥18 | Patients referred to arrhythmia and cardiac implantable electronic device (CIED) outpatient clinics for routine follow-up | Italy | Italian [inferred from the country focus] | n=300 | DHLI | Survey, – | Limitations of surveys   - Unspecified “obvious inherent limitations” of questionnaire-based studies |
| Wångdahl et al., 2020 | The Swedish Version of the Electronic Health Literacy Scale: Prospective Psychometric Evaluation Study Including Thresholds Levels | English | “[T]o translate and adapt eHEALS into a Swedish version; evaluate convergent validity and psychometric properties; and determine threshold levels for inadequate, problematic, and sufficient eHealth literacy” | ✓ | 2019 | 2 [test-retest] | ≥18 | – | Sweden | Swedish | n=323 | eHEALS | Survey, – | Self-report bias:   - “Self-reported eHealth literacy”; potential over- or underestimation of eHL due to self-assessment (“depending on things like the person’s level of self-efficacy”) |
| Wångdahl et al., 2021 | Arabic Version of the Electronic Health Literacy Scale in Arabic-Speaking Individuals in Sweden: Prospective Psychometric Evaluation Study | English | “[T]o translate and adapt the original English eHEALS version into Arabic and to evaluate its psychometric properties” | ✓ | 2019 | 2 [test-retest] | ≥18 | – | Sweden | Arabic | n=298 | eHEALS | Survey, – | – |
| Wecker et al., 2024 | Patient journey and disease-related digital media usage: A cross-sectional study among dermatology patients across Germany | English | “[T]o fill the gap of evidence-based data by examining the impact of health-related digital media use’s impact on the patient-physician relationship and patient journey, with the goal of driving positive changes in office-based dermatological practice” | – | 2022 | 1 | ≥18 | Individuals with previous or current dermatological conditions | Germany | German | n=522 [digital media users for whom eHL was assessed] | eHEALS | Survey, online, paper-based | Selection bias:   - Unspecified “selection bias” (“[d]ue to the relatively low paper questionnaire response rate (36.3%) versus online responses and the voluntary nature of the study”) |
| Wetzel et al., 2024 | Only the anxious ones? Identifying characteristics of symptom checker app users: a cross-sectional survey | English | “[T]o identify meaningful predictors for SCA [symptom checker application] use considering user characteristics” | – | 2020–2021 | 1 | ≥18 [reported in the trial registration] | SCA users and non-users | Germany | German | n=134 [included in final analysis] | eHEALS | Survey, online, paper-based | – |
| Zrubka et al., 2020 | Exploring eHealth Literacy and Patient-Reported Experiences With Outpatient Care in the Hungarian General Adult Population: Cross-Sectional Study | English | “[T]o explore the relationship between eHealth literacy and patient-reported experience measures (PREMs) among users of outpatient care in Hungary” | – | 2019 | 1 | “[A]dult population”, age groups: 18–≥65 years | Individuals who had face-to-face appointment with a healthcare professional in the previous 12 months and reported whether or not they visited their regular healthcare professional | Hungary | Hungarian | n=666 | eHEALS | Survey, online | Self-report bias:   - “[L]ow correlation with objective measures”; “rather self-efficacy related to eHealth literacy than actual skills” |
| Zrubka et al., 2022 | Validation of the PAM‑13 instrument in the Hungarian general population 40 years old and above | English | “[A]dapted and validated the 13-item Patient Activation Measure (PAM-13) in the ≥40 years old Hungarian general population” | – | 2020 | 1 [eHL results not reported for retest analysis ] | ≥40 | – | Hungary | Hungarian | n=779 | eHEALS | Survey, online | Selection bias:   - Overrepresentation of “highly educated, affluent urban respondents” due online data collection |
| Zwierczyk et al., 2022 | Eating Choices–The Roles of Motivation and Health Literacy: A Cross-Sectional Study | English | “[A]nalysis of determinants of food choices” | – | 2022 | 1 | “[A]dult” | Individuals using the internet | Poland | Polish | n=2,008 | eHEALS | Survey, online [computer-based web interviews] | Selection bias:   - Inclusion of exclusively internet users due to “the survey technique”, underrepresentation of “[o]lder persons and those with lower income” |
| Zwierczyk et al., 2023 | The Awareness of the Role of Commercial Determinants of Health and the Readiness to Accept Restrictions on Unhealthy Food Advertising in Polish Society | English | “[A]ssessed the awareness of CDoH [commercial determinants of health] and the attitudes toward potential restrictions on advertising, as well as fiscal interventions targeting food products with harmful effects on health in Polish society” | – | 2022 | 1 | ≥18 | Individuals using the internet | Poland | Polish | n=2,008 | eHEALS | Survey, online [computer-assisted web interviews] | – |

^a^A check mark was set if the objective to validate an original, translated or adapted version of an eHealth literacy measurement instrument was reported in the abstract or introduction.

^b^If the use of all items or subscales intended for calculating eHL scores was reported, the name of the original eHealth literacy measurement was extracted.

^c^eHEALS: eHealth Literacy Scale.

^d^eHLQ: eHealth Literacy Questionnaire.

^e^READHY: Readiness and Enablement Index for Health Technology.

^f^HLS_19_-DIGI: Health Literacy Survey 2019–2021 DIGI.

^g^eHEALS-Carer: eHealth Literacy Scale for Carers of People with Chronic Diseases.

^h^eHEALS-E: Revised eHealth Literacy Scale-Extended.

^i^eHLUS: eHealth Literacy and Use Scale.

^j^DHLI: Digital Health Literacy Instrument.

^k^TeHLI: Transactional eHealth Literacy Instrument.
