# Supplementary material for: Measuring eHealth Literacy in the European Economic Area, Switzerland, and the United Kingdom: Scoping Review
Source: J Med Internet Res. 2026 May 22;28:e87461. doi: 10.2196/87461 (PMC13200168; doi:10.2196/87461)
Supplement: Multimedia Appendix 4 [file jmir-v28-e87461-s004.docx]

## **Multimedia Appendix 4: Characteristics of the Identified Original eHealth Literacy Measurement Instruments**

| **Instrument** | **Author, year** | **Title** | **Objective** | **Underlying concept/instrument** | **Dimensionality** | **Items** | **Measurement approach, response scales** |
| --- | --- | --- | --- | --- | --- | --- | --- |
| eHEALS^a^ | Norman and Skinner, 2006 | eHEALS: The eHealth Literacy Scale | “[M]easure consumers’ combined knowledge, comfort, and perceived skills at finding, evaluating, and applying electronic health information to health problems” | Lily model | Unidimensional; supplementary items | 8+2 | Self-report, 5-point Likert scale (1=“strongly disagree”, 5=“strongly agree”) |
| eHLQ^b^ | Kayser et al., 2018 | A Multidimensional Tool Based on the eHealth Literacy Framework: Development and Initial Validity Testing of the eHealth Literacy Questionnaire (eHLQ) | “[T]o understand and evaluate people’s interaction with digital health services” | eHLF^c^ | Multidimensional: (1) Using technology to process health information, (2) Understanding health concepts and languages, (3) Ability to actively engage with digital services, (4) Feel safe and in control, (5) Motivated to engage with digital services, (6) Access to digital services that work, (7) Digital services that suit individual needs | 35 | Self-report, 4-point scale (1= “strongly disagree”, 4=“strongly agree”) |
| HLS_19_-DIGI^d^ | HLS_19_ Consortium of the WHO Action Network M-POHL, 2022 | The HLS_19_-DIGI Instrument to measure Digital Health Literacy | “[M]easuring digital health literacy in general adult populations” | DHLI^e^, eHEALS | HL-DIGI (“Digital Health Literacy – dealing with digital health information”): unidimensional, no subscales; accompanied by two additional questionnaires: HL-DIGI-INT (“[i]nteraction with digital resources for health”), HL-DIGI-DD (“[u]se of digital devices for health”) | HL-DIGI: 8+HL-DIGI-INT: 2, HL-DIGI-DD: 6 | Self-report, 4-point Likert scale (1=“very difficult”, 4=“very easy”) (HLS-DIGI-INT: analogous to HL-DIGI, HL-DIGI-DD: frequency scale and “not relevant for me” option) |
| READHY^f^ | Kayser et al., 2019 | Development of the Multidimensional Readiness and Enablement Index for Health Technology (READHY) Tool to Measure Individuals’ Health Technology Readiness: Initial Testing in a Cancer Rehabilitation Setting | “[T]o assess the readiness of potential users of health technologies and digital health services, as well as their degree of enablement” | eHLQ, heiQ^g^, HLQ^h^ | Multidimensional: all 7 eHLQ scales (see above), 4 heiQ subscales: (heiQ3) self-monitoring and insight, (heiQ4) constructive attitudes and approaches, (heiQ 5) skill and technique acquisition, (heiQ 8) emotional distress, 2 HLQ subscales: (HLQ1) feeling understood and supported by health care providers, (HLQ4) social support for health | 65 | Self-report, 4-point Likert scale (1=“strongly disagree”, 4=“strongly agree”) |
| DHLI | Van der Vaart et al., 2017 | Development of the Digital Health Literacy Instrument: Measuring a Broad Spectrum of Health 1.0 and Health 2.0 Skills | “To measure the complete spectrum of Health 1.0 and Health 2.0 skills, including actual competencies” | Skills identified in a qualitative bottom-up study | Multidimensional: (1) operational skills, (2) navigation skills, (3) information searching, (4) evaluating reliability, (5) determining relevance, (6) adding content, (7) protecting privacy; supplementary set of performance-based items | 21+7 | Self-report, 4-point scale (1=“very difficult”, 4=“very easy”, 1=“never”, 4=“often”); supplementary set of performance-based items, multiple choice |
| eHLUS^i^ | Stephan et al., 2025 | Development and validation of the eHealth Literacy and Use Scale (eHLUS) to measure medical app literacy | “[T]o measure health literacy in the context of using medical apps” | Theoretical framework developed based on literature search, German eHEALS | Multidimensional: (1) autonomous use and technical access, (2) eHealth engagement, (3) eHealth literacy | 14 | Self-report, 5-point Likert scale (1=“I fully agree”, 5=“I disagree”) |
| Revised eHEALS-E^j^ | Petrič and Atanasova, 2024 | Validation of the extended e-health literacy scale: structural validity, construct validity and measurement invariance | “[R]evise[…] the original eHEALS-E scale to enhance its applicability to the general population and its relevance in contemporary e-health contexts” | Initial eHEALS-E, eHEALS, Norman and Skinner‘s 2006 definition of eHL | Multidimensional: (1) Awareness of Sources, (2) Validating Information, (3) Recognizing Quality, (4) Perceived Efficiency, (5) Smart on the Net, (6) Understanding Information | 26 | Self-report, 5-point Likert scale (1=“does not apply at all”, 5=“applies completely”) |
| eHEALS-Carer^k^ | Efthymiou et al., 2019 | Adapting the eHealth Literacy Scale for Carers of People With Chronic Diseases (eHeals-Carer) in a Sample of Greek and Cypriot Carers of People With Dementia: Reliability and Validation Study | “[A]ssessment of eHealth literacy level of carers” | eHEALS | Multidimensional: (1) information seeking, (2) evaluation | 8 | Self-report, 5-point Likert scale (1=“strongly disagree”, 5=“strongly agree”) |
| TeHLI^l^ | Paige et al., 2019 | Transactional eHealth Literacy: Developing and Testing a Multi-Dimensional Instrument | “[T]o measure patients’ perceived skills related to their capacity to understand, exchange, evaluate, and apply health information from diverse online sources and multimedia” | TMeHL^m^ | Multidimensional: (1) functional eHealth literacy, (2) communicative eHealth literacy, (3) critical eHealth literacy, (4) translational eHealth literacy | 18 | Self-report, 5-point Likert scale (1=“strongly disagree”, 5=“strongly agree”) |

^a^eHEALS: eHealth Literacy Scale.

^b^eHLQ: eHealth Literacy Questionnaire.

^c^eHLF: eHealth Literacy Framework.

^d^HLS_19_-DIGI: Health Literacy Survey 2019–2021 DIGI.

^e^DHLI: Digital Health Literacy Instrument.

^f^READHY: Readiness and Enablement Index for Health Technology.

^g^heiQ: Health Education Impact Questionnaire.

^h^HLQ: Health Literacy Questionnaire.

^i^eHLUS: eHealth Literacy and Use Scale.

^j^ eHEALS-Carer: Revised eHEALS-E: Revised eHealth Literacy Scale-Extended.

^k^eHealth Literacy Scale for Carers of People with Chronic Diseases.

^l^TeHLI: Transactional eHealth Literacy Instrument.

^m^TMeHL: Transactional Model of eHealth Literacy.
