# Supplementary material for: Measuring eHealth Literacy in the European Economic Area, Switzerland, and the United Kingdom: Scoping Review
Source: J Med Internet Res. 2026 May 22;28:e87461. doi: 10.2196/87461 (PMC13200168; doi:10.2196/87461)
Supplement: Multimedia Appendix 5 [file jmir-v28-e87461-s005.docx]

## **Multimedia Appendix 5: Characteristics of the eHealth Literacy Measurement Instruments Validated in Included Papers**

| **eHealth literacy measurement instrument** | **Author, year** | **Title** | **New eHealth literacy measurement instrument** | **Version of an existing eHealth literacy measurement instrument** | **Country** | **Language** | **Population** | **Participants** | **Factorial structure** | **Cronbach** $\boldsymbol{\alpha}$ | **McDonald** $\boldsymbol{\omega}$ |
| --- | --- | --- | --- | --- | --- | --- | --- | --- | --- | --- | --- |
| eHEALS^a^ | Bäuerle et al., 2023 | Psychometric properties of the German revised version of the eHealth literacy scale in individuals with cardiac diseases: Validation and test of measurement invariance | – | ✓ | Germany | German | Individuals with a diagnosis of a cardiac disease (at least coronary artery disease or congestive heart failure) and internet access | n=315 [participants who completed the eHEALS items] | 2: (1) Information seeking, (2) Information appraisal | .94, (1) .92, (2) .91 | – |
| eHEALS | Brørs et al., 2020 | Psychometric Properties of the Norwegian Version of the Electronic Health Literacy Scale (eHEALS) Among Patients After Percutaneous Coronary Intervention: Cross-Sectional Validation Study | – | ✓ | Norway | Norwegian | Patients undergoing percutaneous coronary intervention, living at home, with access to electronic equipment and the internet | n=1,659 [participants with eHEALS scores] | 3: (1) Awareness, (2) Skills, (3), Evaluate | >.99, 2-week retest .94 | – |
| eHEALS | Burzyńska et al., 2022 | Evaluating the Psychometric Properties of the eHealth Literacy Scale (eHEALS) among Polish Social Media Users | – | ✓ | Poland | Polish | Individuals with a verified account on at least one social platform | n=1,527 | 1 | .84 | – |
| eHEALS | Chaniaud et al., 2022 | Translation and Validation Study of the French Version of the eHealth Literacy Scale: Web-Based Survey on a Student Population | – | ✓ | France | French | University students | n=328 | 2: (1) Information seeking, (2) Information appraisal | .89 | – |
| eHEALS | Dale et al., 2020 | Testing Measurement Properties of the Norwegian  Version of Electronic Health Literacy Scale (eHEALS) in a Group of Day Surgery Patients | – | ✓ | Norway | Norwegian | Patients scheduled for day surgery | n=109 | 2: (1) Information awareness and seeking, (2) Ability to evaluate and act | .89 | – |
| eHEALS | Efthymiou et al., 2025 | Validation of the eHealth literacy scales: comparison between the shorter and longer versions | – | ✓ | Greece | Greek | Individuals | n=401 | 2: (1) Awareness of resources, (2) Evaluation | .91, (1): .88, (2) .78 | – |
| eHEALS | Geiger et al., 2024 | Association between eHealth literacy and health outcomes in German athletes using the GR-eHEALS questionnaire: a validation and outcome study | – | ✓ | Germany | German | Individuals being athletes with internet access | n=282 [participants with complete data on the eHEALS] | 2: (1) Information seeking, (2) Information appraisal | .93, (1) .89, (2) .86 | – |
| eHEALS | Georgsson et al., 2025 | Validation of the eHealth Literacy Scale Instrument in a Restless Legs Syndrome Population: Classical Test Theory and Rasch Analysis Study | – | ✓ | Sweden | Swedish | Individuals diagnosed and treated for restless legs syndrome | n=788 | 1 | .968 | .968 |
| eHEALS | Juvalta et al., 2020 | Electronic Health Literacy in Swiss-German Parents: Cross-Sectional Study of eHealth Literacy Scale Unidimensionality | – | ✓ | Switzerland | German | Parents of children aged 1– 24 months | n=703 | 1 | – | .89 (online sample: .90, paper-based sample: .89) |
| eHEALS | Luz et al., 2025 | Psychometric Analysis of the eHealth Literacy Scale in Portuguese Older Adults (eHEALS-PT24): Instrument Development and Validation | – | ✓ | Portugal | Portuguese | Primary care users | n=381 (study 1: n=80,  study 2: n=301) | 1 | .92 (study 1), .98 (study 2) | – |
| eHEALS | Marsall et al., 2022 | Measuring Electronic Health Literacy: Development, Validation, and Test of Measurement Invariance of a Revised German Version of the eHealth Literacy Scale | – | ✓ | Germany | German | Individuals | n=470 | 2: (1) Information seeking, (2) Information appraisal | (1) .92, (2) .83 | – |
| eHEALS | Marsall et al., 2024 | Assessing Electronic Health Literacy in Individuals With the Post–COVID-19 Condition Using the German Revised eHealth Literacy Scale: Validation Study | – | ✓ | Germany | German | Individuals with inter-net access who had a confirmed COVID-19 infection in the past, and re-ported current post COVID-19 symptoms | n=330 | 2: (1) Information seeking, (2) Information appraisal | .91, (1) .90, (2) .86 | – |
| eHEALS | Oliveira et al., 2024 | From Validation to Assessment of e-Health Literacy: A Study among Higher Education Students in Portugal | – | ✓ | Portugal | Portuguese | Higher education students | n=245 | 1 | .850 | – |
| eHEALS | Wångdahl et al., 2020 | The Swedish Version of the Electronic Health Literacy Scale: Prospective Psychometric Evaluation Study Including Thresholds Levels | – | ✓ | Sweden | Swedish | Individuals | n=323 | 1 | .94 | – |
| eHEALS | Wångdahl et al., 2021 | Arabic Version of the Electronic Health Literacy Scale in Arabic-Speaking Individuals in Sweden: Prospective Psychometric Evaluation Study | – | ✓ | Sweden | Arabic | Individuals | n=298 | 1 | .92 | – |
| eHLQ^b^ | Hermansen et al., 2023 | Preliminary validity testing of the eHealth Literacy Questionnaire (eHLQ): a Confirmatory Factor Analysis (CFA) in Norwegian hospitalized patients | – | ✓ | Norway | Norwegian | Hospitalized patients | n=260 | 7: (1) Using technology to process health information, (2) Understanding health concepts and language, (3) Ability to actively engage with digital services, (4) Feel safe and in control, (5) Motivated to engage with digital services, (6) Access to digital services, (7) Digital services that suit individual needs | (1) .85, (2) .73, (3) .90, (4) .83, (5) .84, (6) .76, (7) .84 | – |
| eHLQ | Hernández Encuentra et al., 2024 | Spanish and Catalan Versions of the eHealth Literacy Questionnaire: Translation, Cross-Cultural Adaptation, and Validation Study | – | ✓ | Spain | Catalan, Spanish | Individuals | n=800 | 7: (1) Using technology to process health information, (2) Understanding of health concepts and language, (3) Ability to actively engage with digital services, (4) Feel safe and in control, (5) Motivated to engage with digital services, (6) Access to digital services that work, (7) Digital services that suit individual needs | Spanish version: (1) .90, (2) .85, (3) .92, (4) .90, (5) .90, (6) .88, (7): .92; Catalan version: (1) .91, (2) .85, (3) .92, (4) .90, (5) .90, (6) .88 (7) .92 | Spanish version: (1) .92, (2) .87, (3) .92, (4) .90, (5) .92, (6) .89, (7) .90; Catalan version: (1) .91, (2) .85, (3) .92, (4) .90, (5) .91, (6) .89, (7) .91 |
| eHLQ | Poot et al., 2023 | Translation, cultural adaptation and validity assessment of the Dutch version of the eHealth Literacy Questionnaire: a mixed-method approach | – | ✓ | Netherlands | Dutch | Individuals with internet access | n=1,650 | 7: (1) Using technology to process health information, (2) Engagement in own health, (3) Ability to actively engage with digital services, (4) Feel safe and in control, (5) Motivated to engage with digital services, (6) Access to digital services that work, (7) Digital services that suit individual needs | (1) .67, (2) .68, (3) .79, (4) .80, (5) .67, (6) .71, (7) .74 | – |
| eHLQ | Sjöström et al., 2023 | The Swedish Version of the eHealth Literacy Questionnaire: Translation, Cultural Adaptation, and Validation Study | – | ✓ | Sweden | Swedish | Patients visiting primary health care centers and parents of hospitalized children | n=236 | 7: (1) Using technology to process health information, (2) Understanding of health concepts and language, (3) Ability to actively engage with digital services, (4) Feel safe and in control, (5) Motivated to engage with digital services, (6) Access to digital services that work, (7) Digital services that suit individual needs | (1) .88, (2) .82, (3) .92, (4) .83, (5) .87, (6) .82, (7) .90 | – |
| HLS19-DIGI^c^ | Levin-Zamir et al., 2025 | Measuring digital health literacy and its associations with determinants and health outcomes in 13 countries | – | ✓ | Austria, Belgium, Czechia, Denmark, France, Germany, Hungary, Ireland, Israel, Norway, Portugal, Slovakia, Switzerland | Austria, Germany: German; Belgium: Dutch, French; Czechia: Czech; Denmark: Danish; France: French; Hungary: Hungarian; Ireland: English; Israel: Hebrew, Arab, Russian; Norway: Norwegian; Portugal: Portuguese; Slovakia: Slovak; Switzerland: French, German, Italian | Individuals | n=28,057 (1,000–4,487 per country) | HLS19-DIGI-HI: 1 | Mean: .83, Austria: .81, Belgium: .86, Czechia: .82, Denmark: .86, France: .86, Germany: .83, Hungary: .79, Ireland: .79, Israel: .83, Norway: .77, Portugal: .83, Slovakia: .87, Switzerland: .85 | – |
| eHLUS^d^ | Stephan et al., 2025 | Development and validation of the eHealth Literacy and Use Scale (eHLUS) to measure medical app literacy | ✓ | – | Germany | German | Individuals with psychological burdens | n=117 | 3: (1) Autonomous Use and Technical Access, (2) eHealth Engagement; (3) eHealth Literacy | .913, (1) .855, (2) .925, (3) .839 | .911 |
| Revised eHEALS-e^e^ | Efthymiou et al., 2025 | Validation of the eHealth literacy scales: comparison between the shorter and longer versions | – | ✓ | Greece | Greek | Individuals | n=401 | 5: (1) Awareness resources and recognizing quality, (2) Understanding Information, (3) Smart on the Net, (4) Accessibility and Validity of Information, (5) Perceived Efficiency | .80, (1) .89, (2) .85, (3) .75, (4) .62, (5) .80 | – |
| Revised eHEALS-e | Petrič and Atanasova, 2024 | Validation of the extended e-health literacy scale: structural validity, construct validity and measurement invariance | ✓ | – | Slovenia | Slovenian | Individuals who at least occasionally used one internet service to gain health-related information | n=1,944 | 6: (1) Awareness of Sources, (2) Validating Information, (3) Recognizing Quality, (4) Perceived Efficiency (5) Smart on the Net, (6) Understanding Information | 0.89, (1) .77, (2) .69, (3) .85, (4) .82, (5) .80, (6) .79 | – |
| TeHLI^f^ | Smoła et al., 2024 | Transactional e-health literacy and its association with e-health services use in Polish adults: a cross-sectional study | – | ✓ | Poland | Polish | Individuals using the internet | n=1,661 | 4: (1) Functional, (2) Communicative, (3) Critical, (4) Translational | (1) .892, (2) .903, (3) .896, (4) .880 | – |

^a^eHEALS: eHealth Literacy Scale.

^b^eHLQ: eHealth Literacy Questionnaire.

^c^HLS_19_-DIGI: Health Literacy Survey 2019–2021 DIGI.

^d^eHLUS: eHealth Literacy and Use Scale.

^e^Revised eHEALS-E: Revised eHealth Literacy Scale-Extended.

^f^TeHLI: Transactional eHealth Literacy Instrument.
